# Microwave-Assisted Atom Transfer Radical Cyclization in the Synthesis of 3,3-Dichloro-γ- and δ-Lactams from *N*-Alkenyl-Tethered Trichloroacetamides Catalyzed by RuCl_2_(PPh_3_)_3_ and Their Cytotoxic Evaluation

**DOI:** 10.3390/molecules29092035

**Published:** 2024-04-28

**Authors:** Faïza Diaba, Alexandra G. Sandor, María del Carmen Morán

**Affiliations:** 1 Laboratori de Química Orgànica, Facultat de Farmàcia i Ciències de l’Alimentació, IBUB, Universitat de Barcelona, Av. Joan XXIII 27-31, 08028 Barcelona, Spain; 2 Departament de Bioquímica i Fisiologia-Secció de Fisiologia, Facultat de Farmàcia i Ciències de l’Alimentació, Universitat de Barcelona, Avda. Joan XXIII 27-31, 08028 Barcelona, Spain; mcmoranb@ub.edu; 3 Institut de Nanociència i Nanotecnologia—IN2UB, Universitat de Barcelona, Avda. Diagonal, 645, 08028 Barcelona, Spain

**Keywords:** γ-lactams, δ-lactams, atom transfer radical cyclization, cytotoxic, tris(triphenylphosphine)ruthenium(II) dichloride, squamous carcinoma cells

## Abstract

An expeditious synthesis of γ- and δ-lactams from tethered alkenyl trichloroacetamides in the presence of 5% of RuCl_2_(PPh_3_)_3_ is reported. In this investigation we have demonstrated that microwave activation significantly enhances reaction rates, leading to the formation of the corresponding lactams in yields ranging from good to excellent. Thus, we have been able to prepare a wide range of lactams, including indole and morphan bicyclic scaffolds, where the corresponding reactions were completely diastereoselective. This process was successfully extended to α,α-dichloroamides without affecting either their yield or their diastereoselectivity. Some of the lactams prepared in this work were evaluated for their hemolytic and cytotoxic responses. All compounds were found to be non-hemolytic at the tested concentration, indicating their safety profile in terms of blood cell integrity. Meanwhile, they exhibited interesting cytotoxicity responses that depend on both their lactam structure and cell line. Among the molecules tested, γ-lactam **2a** exhibited the lowest IC_50_ values (100–250 µg/mL) as a function of its cell line, with promising selectivity against squamous carcinoma cells (A431) in comparison with fibroblasts (3T3 cell line).

## 1. Introduction

Of the radical processes, atom transfer radical additions (ATRAs) and atom transfer radical cyclizations (ATRCs) from halo derivatives are considered important and useful tools for C-C bond formation [1,2,3,4]. In contrast to the radical reductive procedures in these types of reactions, known also as Kharasch additions or cyclizations, the halogen present in the substrate and whose abstraction is part of the propagation step, is retained in the final compound. As a consequence, the generated products possess a potentially useful carbon–halogen bond that allows post-cyclization manipulation if required to access more advanced structures [5,6,7,8]. ATRAs and ATRCs in the presence of transition metal catalysts specially derived from iron, copper, ruthenium or nickel are well documented [9,10,11,12]. Of the ruthenium catalysts, tris(triphenylphosphine)ruthenium(II) dichloride (RuCl_2_(PPh_3_)_3_) was used for the first time by Matsumoto et al. in an intermolecular ATRA of CCl_4_ and CHCl_3_ to terminal olefins [13] and then to cyclohexene [14], and later they reported a radical reaction of dichloro- and trichloroacetic acid esters with 1-olefins [15]. Since then, ATRCs from alkenyl-tethered trichloroacetamides in the presence of RuCl_2_(PPh_3_)_3_ have been reported in a few examples, mainly for the preparation of γ-lactams [16,17,18,19]. The reactions typically required heating to elevated temperatures (110–170 °C) for extended periods to proceed effectively with poor to acceptable regio- and/or diastereoselectivity.

**Figure 1:**
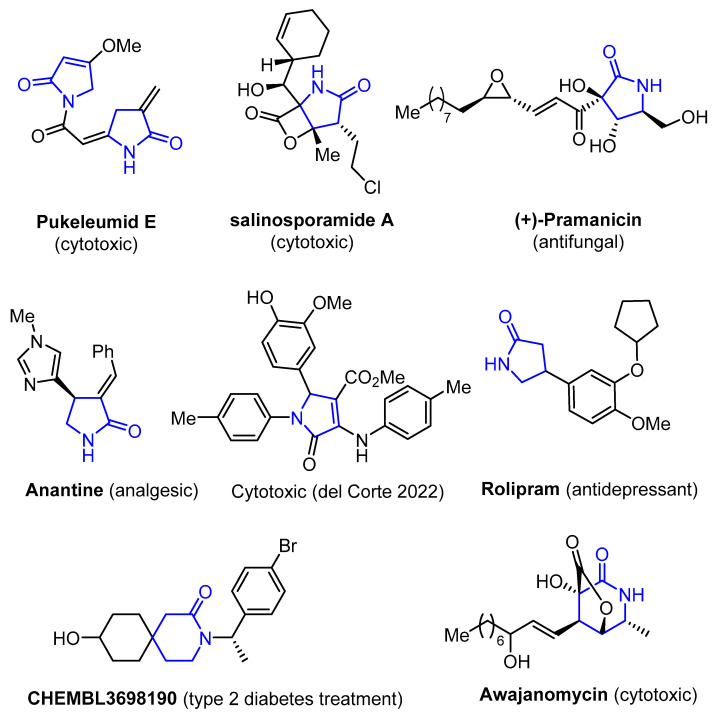
Representative examples of biologically active γ- and δ-lactams [21].

On the other hand, γ- and δ-lactam moieties are found in a long list of natural and non-natural compounds with antibiotic, antifungal, anti-inflammatory and cytotoxic activities, among others [20,21,22,23] (Figure 1).

**Figure 2:**
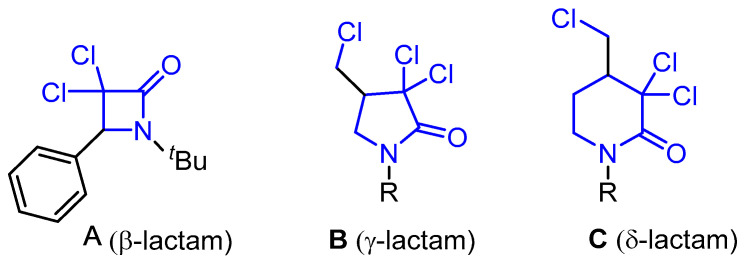
Structure of the γ- and δ-lactams (**B**,**C**) that are the subject of this study and β-lactam **A**.

Recently, we reported the synthesis of a series of 3,3-Dichloro-β-Lactams from benzyl-tethered trichloroacetamides through an unprecedented microwave-assisted benzylic C-H activation catalyzed by RuCl_2_(PPh_3_)_3_ [24]. Six of the lactams prepared in this study were assessed for their hemolytic and cytotoxic characteristics, revealing non-hemolytic behavior and interesting cytotoxic activity. Compound **A** (Figure 2) was found to be the most cytotoxic, showing the lowest IC_50_ values (20–49 g/mL and 30–47 g/mL) for the HaCaT and A431 cell lines, respectively. As a continuation of this work, we decided to investigate the ATRCs from *N*-alkenyl trichloroacetamides in the presence of RuCl_2_(PPh_3_)_3_ and under microwave activation for the preparation of 3,3-dichloro-γ- and δ-lactams (**B**,**C**), with the aim of improving the reaction conditions, the yield and also the stereoselectivity. Additionally, some of the lactams prepared in this study were investigated for their cytotoxic activity.

## 2. Results

### 2.1. Synthesis of γ- and δ-Lactams Using Microwave Activation

To optimize the reaction conditions, we carried out our primary study on trichloroacetamide **1a** with a *t*-butyl group on the nitrogen, since the presence of the latter can accelerate the reaction (Table 1).

The first reaction from **1a** was achieved using the conditions reported in the literature [16] but using microwave activation. Thus, when **1a** was heated to 140 °C in toluene instead of benzene, with 5 mol% of catalyst, no reaction took place even after 1 h of irradiation (entry 1). Nevertheless, microwave irradiation at 160 °C allowed the reaction to take place using the same catalyst loading. Hence, a full conversion was observed after only 15 min of reaction, leading to γ-lactam **2a** with an excellent yield (entry 4). Shortening the reaction time or decreasing the catalyst loading did not bring any improvement to the reaction yield (entries 5–7). It is worth noting that when the reaction was performed in acetonitrile (ACN) and under the optimized conditions, unreacted **1a** was recovered (entry 8). It is important to mention that the usage of conventional heating instead of microwave activation provided a lower conversion and thus a lower yield of **2a** (entry 9). The reaction followed a similar scenario when Grubbs second-generation catalyst was used instead of RuCl_2_(PPh_3_)_3_, since the reaction was also incomplete (entry 10).

The best conditions were then applied to *N*-allyl-2,2,2-trichloroacetamides **1b**–**1f** featuring diverse nitrogen substituents. As is indicated in Table 2, the reaction outcome remains largely unaffected by the nature of the substituent attached to the nitrogen atom, since the corresponding γ-lactams **2b**-**2f** were isolated with very good yields. Nevertheless, an extended reaction time was necessary to attain a better yield in the absence of a substituent (R = H, **1b**) and with the phenyl derivative **1d** (entries 2 and 5).

**Scheme 1:**
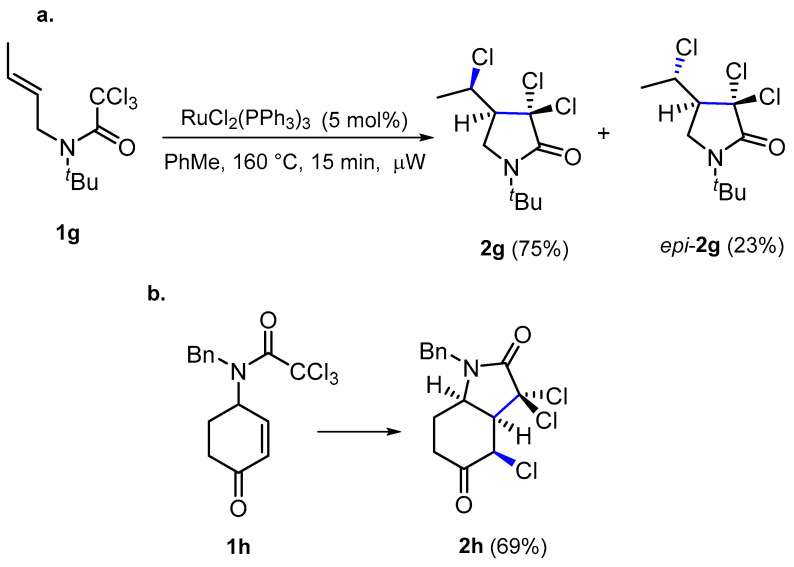
ATRC from trichloroacetamide **1g** (**a**) and **1h** (**b**).

Next, we studied the reaction of trichloroacetamides **1g** and **1h** with a substituted allyl chain. In the case of **1g**, the reaction proceeded with an excellent yield and good stereoselectivity (Scheme 1a). Meanwhile, for trichloroacetamide **1h** under the same reaction conditions, a completely diastereoselective ATRC took place to provide indole derivative **2h** alone, with a good yield (Scheme 1b).

**Scheme 2:**
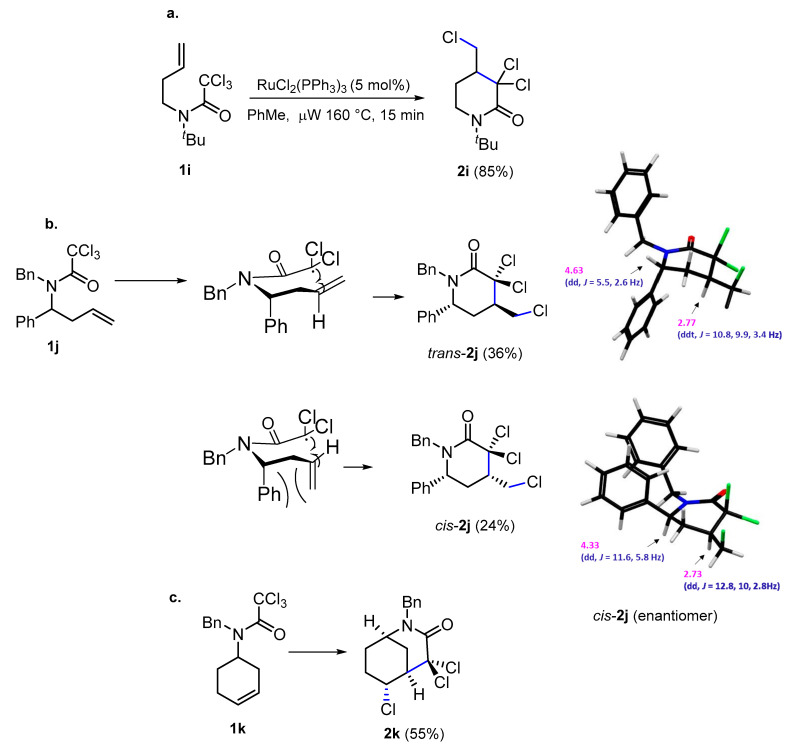
ATRCs from trichloroacetamides **1i** (**a**)**, 1j** (**b**) and **1k** (**c**) in the synthesis of δ-lactams.

The set of the optimized conditions was successfully extended to butenyl derivative **1i**, providing the corresponding δ-lactam **2i** with an excellent yield (Scheme 2a). The presence of a phenyl at the homoallylic position did not affect the course of the reaction, since trichloroacetamide **1j** followed the same scenario, affording δ-lactam **2j** as a *trans*/*cis* mixture of diastereomers (1.5:1). The latter reflects the stability of the radical intermediates preceding the cyclization, as is indicated in Scheme 2b. This result is in accordance with what was observed previously in the radical cyclization of *N*-(but-3-en-1-yl)-*N*-(tertbutyl)-2-iodoalkanamides, where the 4,6-*trans* configuration is also favored [25]. The identification of *trans*-**2j** and *cis*-**2j** was possible thanks especially to the ^1^H NMR of H-6, as is indicated in Scheme 2b. Finally, from trichloroacetamide **1k**, we were able to access the morphan derivative **2k [26]** as a single diastereomer with an acceptable yield (Scheme 2c).

Additionally, to assess the necessity of the three chloro atoms to facilitate radical formation and hence promote cyclization, we conducted the reaction under the optimized conditions from **1l**, **1m** and **1n,** wherein one of the chloro atoms was replaced by a hydrogen, a methyl and an ester group, respectively. In all instances, the reaction progresses successfully, providing the corresponding γ-lactams **2l**, **2m** and **2n**, with good yields, as mixtures of *trans*/*cis* isomers (Table 3).

**Figure 3:**
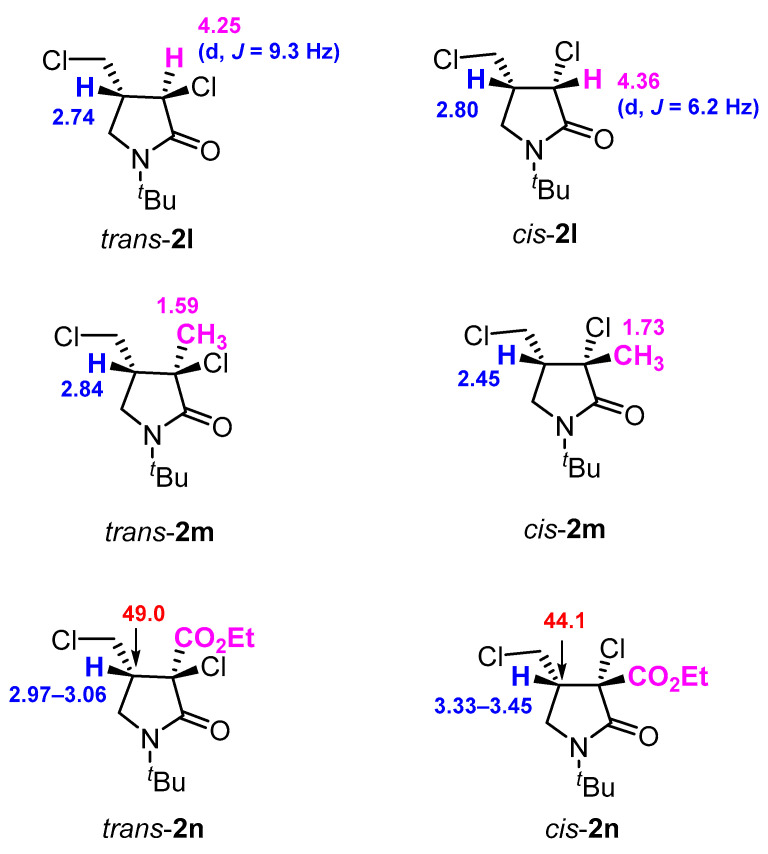
Selected NMR data for the identification of the *trans* and the *cis* isomers in **2l**, **2m** and **2n** γ-lactams.

The diastereomers formed in each case were identified by NMR spectroscopy (Figure 3). For lactam **2l**, the determination of its *trans*/*cis* isomers at C-3, C-4 is set by the ^1^H NMR coupling constants *J*_3,4_. While the *trans*-isomer exhibits *J*_3,4_ of ca. 9.3 Hz, the *cis*-isomer displays a *J*_3,4_ of ca. 6.2 Hz. Moreover, the H-3 and H-4 in *trans*-**2l** are shielded (δ 4.25 and 2.74) with regard to those of *cis*-**2l** (δ 4.36 and 2.80). Regarding *trans*-**2m** and *cis*-**2m**, their assignation was possible thanks mainly to the revealing chemical shift of the proton at C-4, which appears at 2.84 ppm in *trans*-**2m** and at 2.45 in *cis*-**2m [27]**. Finally, *cis*-**2n** and *trans*-**2n** were assigned through the chemical shift of C-4, which was at δ 49.0 ppm in *trans*-**2n** and 44.1 ppm in *cis*-**2n [28]**.

**Scheme 3:**
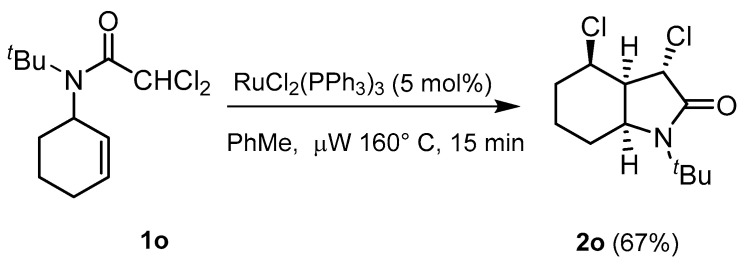
ATRC from trichloroacetamide **1o**.

Finally, from dichloroacetamide **1o**, a diastereoselective cyclization takes place providing, after 15 min of irradiation under the same conditions reported previously, indole derivative **2o** alone, with a good yield (Scheme 3). For NMR spectra of the products involved in this study see Supplementary Materials.

**Figure 4:**
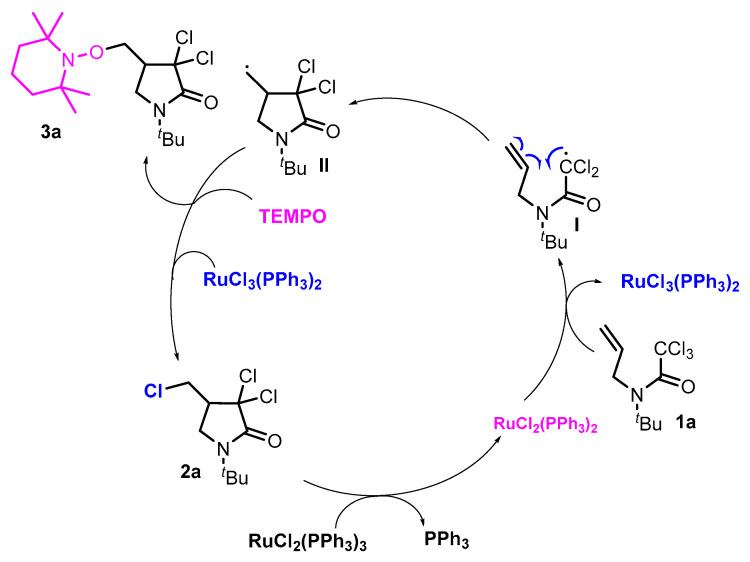
Proposed mechanism for the formation of lactam **2a** from **1a**.

Eventually, the mechanism involved in the formation of these lactams was ascertained by a control experiment of **1a**, using the optimized conditions but in the presence of 1 equiv. of TEMPO as the radical scavenger. Hence, after 15 min of irradiation only **3a**, identified easily by NMR spectroscopy, was isolated in addition to unreacted **1a**. This result, as expected, confirms the radical mechanism involved in the formation of lactams **2**. As was reported previously [29,30], at the temperature of the reaction, the ruthenium catalyst loses a triphenylphosphine ligand, generating RuCl_2_(PPh_3_)_2_. The latter abstracts a chloro atom from trichloroacetamide **1** to give RuCl_3_(PPh_3_)_2_ complex and (carbamoyl)dichloromethyl radical **I** which evolves into radical **II** through cyclization. Finally, a chlorine atom transfer from RuCl_3_(PPh_3_)_2_ to radical **II** generates lactam **2** and regenerates RuCl_2_(PPh_3_)_2_ complex (Figure 4).

### 2.2. Hemocompatibility Studies and Cell Viability Assays of Selected Lactams

**Figure 5:**
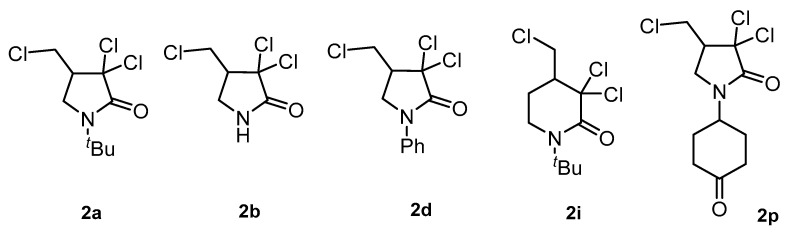
Selected γ- and δ-lactams for hemocompatibility and cytotoxicity studies.

As was mentioned in the introduction, in our previous work [24] we have synthesized a series of β-lactams and found that they have non-hemolytic properties and that their cytotoxic response is highly dependent on their structure and concentration. As a continuation of this investigation, we decided to explore some of the molecules prepared herein for their hemocompatibility and cytotoxic activity. Specifically, our focus will be on investigating the impact of three critical factors: the size of the lactam ring, the nature of the substituent on the nitrogen and the presence of a chloromethyl substituent in the structure. Hence, we selected γ-lactams **2a, 2b**, **2d** and **2p** [31] and δ-lactam **2i** (Figure 5).

#### 2.2.1. Hemocompatibility Studies

**Figure 6:**
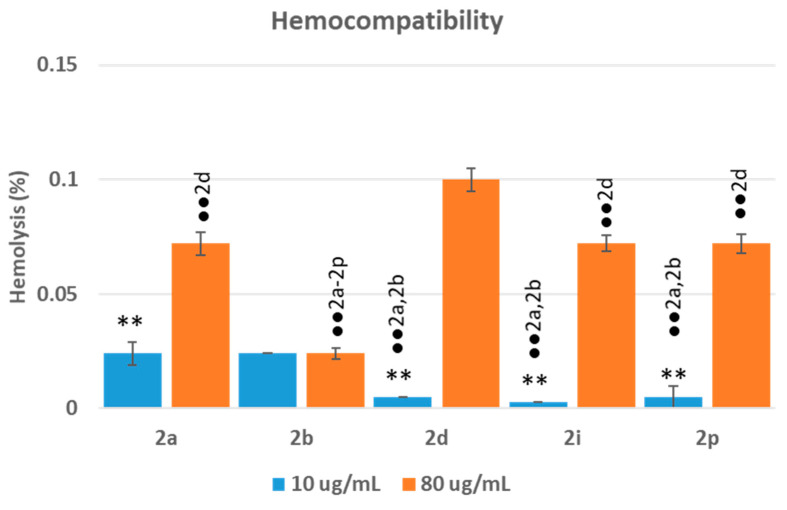
Percentage of hemolysis induced by the selected compounds **2a**, **2b**, **2d**, **2i** and **2p** as a function of concentration. The data correspond to the average of three independent experiments ± standard deviation. ** (*p* < 0.001) indicates significant differences between concentrations for the same compound. ●● (*p* < 0.001) indicates significant differences between compounds for the same concentration.

The assessment of the biological compatibility of new compounds that may come into contact with blood necessitates in vitro hemocompatibility evaluations in accordance with EU regulations (ISO 10993-4) [32]. In this work, the degree of hemolysis produced by the different compounds through their incubation with an erythrocyte suspension was determined. The hemolytic response of compounds **2a**, **2b**, **2d**, **2i** and **2p** was evaluated at two different concentrations (10 and 80 µg/mL). The results obtained with the selected molecules are displayed in Figure 6. The degree of hemolysis fluctuated slightly, with values ranging between 0.003% (compound **2i**), 0.005 (compounds **2d** and **2p**) and 0.02% (compounds **2a** and **2b**) at concentrations equal to 10 µg/mL. At this dose, compounds **2d**, **2i** and **2p** demonstrated significant differences (*p* < 0.001) with regard to compounds **2a** and **2b**. By increasing the concentration up to 80 µg/mL, the hemolytic response remained stable for compound **2b** and increased up to 0.1% for compound **2d**. Compounds **2a**, **2i** and **2p** demonstrated hemolytic responses close to 0.07%. Compound **2d** demonstrated significant differences (*p* < 0.001) with regard to the rest of the compounds. When comparing the effect of varying concentrations, significant differences (*p* < 0.001) between the hemolytic responses at the two different concentrations were found for all the compounds, except compound **2b**. Thus, based on the obtained results, it could be concluded that the proposed compounds showed non-hemolytic properties, considering the criteria used to classify compounds as non-hemolytic, which typically involves values < 2% [33].

#### 2.2.2. Cell Viability Assays

The interaction between new materials and biological systems is crucial for establishing the potential applications of these materials. In this work, we assessed the impact of varying lactam ring sizes (γ or δ), the presence of a chloromethyl substituent in the structure and the nature of the group on the nitrogen through in vitro cell viability assays. In view of the potential antitumoral activity of the proposed compounds, commercially available cell lines with tumoral characteristics were chosen. This work includes keratinocytes with tumoral characteristics, resembling those found in squamous cell carcinoma (SCC), namely the A431 cell line. Squamous cell carcinoma (SCC) stands as the most prevalent form of skin cancer, surpassing all other types in frequency [34]. HeLa is the oldest and most used human cell line, derived from cervical cancer cells. Since they were put into mass production, HeLa cells have been used for research into cancer, AIDS, the effects of radiation and toxic substances, gene mapping and countless other scientific pursuits [35]. Moreover, breast cancer is the most frequent malignancy in females. Due to its major impact on the population, this disease represents a critical public health problem that requires further research at the molecular level to define its prognosis and specific treatment. MCF-7 is a commonly used breast cancer cell line [36]. For comparative purposes, and to assess potential selective toxicity, the murine Swiss albino fibroblast (3T3) cell line was also included in these screening assays. 3T3 fibroblasts are readily available and are closely representative of a physiological model cell line [37].

**Figure 7:**
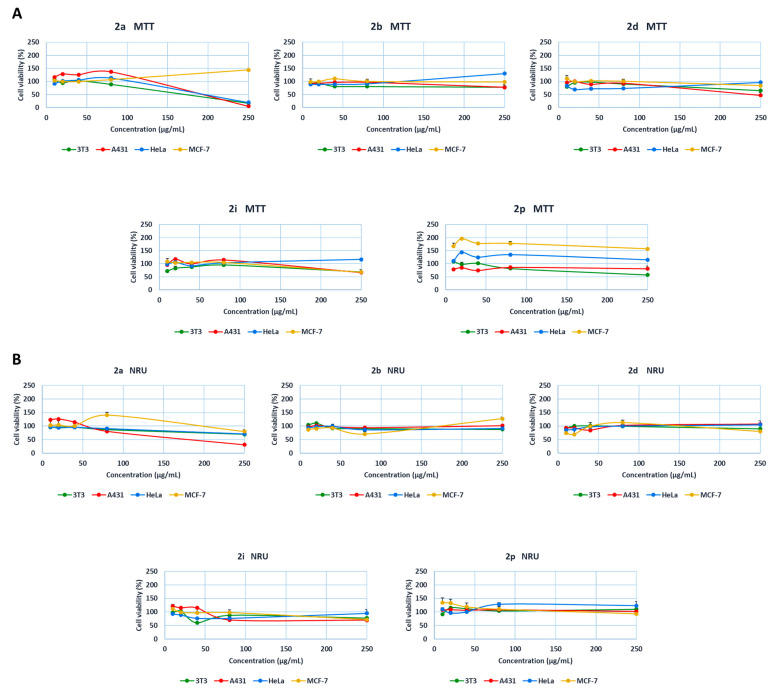
Concentration-dependent viability on the 4 cell lines incubated with the selected γ- and δ-lactam derivatives for 24 h, determined by MTT (**A**) and NRU (**B**) assays. The data correspond to the average of three independent experiments ± standard deviation.

Two different endpoints, 3-(4,5-dimethyl-2-thiazolyl)-2,5-diphenyltetrazolium bromide (MTT) and neutral red uptake (NRU), were used to assess differences in cell-induced cytotoxicity. MTT offers details about the modification of the metabolic activity of the mitochondria inside the cells upon incubation with drugs. In addition, NRU evaluates the interaction of these drugs with the plasmatic membrane. Dose–response curves were determined from the MTT [38] and NRU [39] assays using the proposed four cell lines. Cytotoxicity assays were performed at concentrations ranging between 10 and 250 µg/mL. Figure 7 shows representative results under the assayed conditions.

The obtained results suggest that the compounds are mainly biocompatible in the concentration range 10–80 µg/mL, with cell viability values comparable to those promoted by the control cells. However, by increasing the concentration to 250 µg/mL, the obtained response seems to be a function of both the endpoint method and the lactam structure. The MTT method was demonstrated to be more sensitive at assessing the deleterious effect of the assayed compounds (Figure 7A). Using this method, compounds **2b**, **2i** and **2p** exhibited minimum cell viability values of 60–70% under discrete conditions. Compound **2d** demonstrated selective toxicity against A431 cells, decreasing viability up to 50% at the highest assayed concentration. Moreover, upon incubation with compound **2a**, cell viability decreased by 5% (A431cell line) and 15% (3T3 and HeLa cell lines). It is noteworthy that compound **2p** could promote the proliferation of tumor cells derived from HeLa and MCF-7 under the assayed conditions.

Considering the NRU method (Figure 7B), cell viability values were demonstrated to be a function of the lactam structure, with minimum values ranging between 30% (compound **2a**), 70% (compound **2i**), 80–85% (compound **2b** and **2d**) and 100% (compound **2p**).

As a general trend, these results suggest poor interaction with the plasma membrane and lysosomal accumulation (NRU method) compared to the modification of the metabolic activity of the mitochondria inside the cells (MTT method). In all cases, these compounds can reach the mitochondrial compartment without substantially altering the plasma membrane. These results differed from those found previously in our lab concerning β-lactams’ derivatives [24], for which no differences in the mode of action against the plasmatic membrane (NRU) and metabolic response (MTT) were determined.

The corresponding half-maximal inhibitory concentration (IC_50_) was determined from the fitting of concentration-dependent viability curves. The results obtained are summarized in Table 4. The structural characteristics of γ-lactam derivatives seem to be the main factor influencing the cytotoxic response of the tested compounds. Through the MTT method, γ-lactams **2a** and **2d** were the most cytotoxic products, showing the lowest IC_50_ values (compound **2d** A41: 240 µg/mL and compound **2a** 3T3: 172 µg/mL, A431: 192 µg/mL and HeLa: 195 µg/mL). In the other cases, these compounds’ IC_50_ values must be defined as higher than 250 µg/mL, as this value was the highest assayed concentration. Using the NRU method, only compound **2a,** for the A431 cell line, showed an IC_50_ value different from 250 µg/mL (186 µg/mL).

Moreover, Table 4 also displays their selective toxicity in the form of a selectivity index (SI) against the tumoral cells. Based on the IC_50_ values, mostly defined as >250 µg/mL, the SI values were only properly determined in a few cases. Using the MTT method, positive selective toxicity (SI > 1) was obtained for compound **2d**. When considering compound **2a**, negative selective toxicity values (SI < 1) were found for the three tumoral cell lines. When considering the NRU method, compound **2a** demonstrated positive selective toxicity, with values > 1.35.

**Figure 8:**
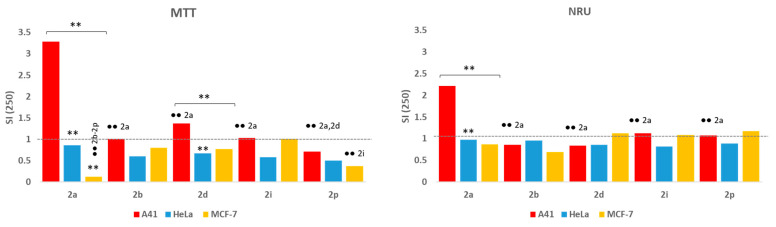
Selective toxicity at the concentration 250 µg/mL of the three tumoral cell lines compared to the 3T3 fibroblast cell line when incubated with lactam derivatives **2a**, **2b**, **2d**, **2i** and **2p** for 24 h, determined by MTT (left) and NRU (right) assays. ** (*p* < 0.001) indicates significant differences between cell lines for the same compound. ●● (*p* < 0.001) indicates significant differences between compounds for the same cell line.

Further insight in the mode of action of these compounds could be found, considering the cellular response at the highest assayed concentration. Figure 8 shows representative results of the SIs at this concentration (SI250).

The results obtained demonstrated a significant dependence on both the cell line and the structure of the γ- or δ-lactam. Thus, in the case of HeLa cell line, none of the conditions tested promote positive selective toxicity, with the SI250 always lower than one, independent of the cytotoxicity endpoint. No significant differences between compounds for this cell line, using both endpoint methods, were found.

When considering the MCF-7 cell line, the SI250 values increased up to one for compound **2i**, using both endpoints, and compounds **2d** and **2p,** when considering their interaction with the cell membrane (NRU method). Significant differences (*p* < 0.001) were found between compound **2i** and **2p** and, in the case of compound **2a**, with all the other compounds when using the MTT method. No significant differences between compounds for this cell line, when using the NRU endpoint, were found.

The tumoral cell line A431 is associated with squamous cell carcinoma and was demonstrated to be the most sensitive cell line, with positive selective toxicity for the five compounds. Using the MTT method, its SI250 values ranged between 1.1 (compounds **2b** and **2i**) and 1.4 (compound **2d**), with a maximum value of 3.3 for compound **2a**. For the NRU method, SI250 values of 1.1 were achieved for compounds **2i** and **2p**, with a maximum value of 2.2 for compound **2a**. Compound **2a** demonstrated significant differences (*p* < 0.001) from all the compounds when using both endpoint methods. In the case of the MTT method, significant differences (*p* < 0.001) were also found between compounds **2d** and **2p**.

When considering significant differences between cell lines, only compound **2d** (MTT method) and compound **2a** (MTT and NRU methods) demonstrated significant differences (*p* < 0.001). Using the metabolic marker (MTT) method, compound **2d** demonstrated significant differences between the A431 and HeLa and MCF-7 cell lines. Similar results were obtained in the case of compound **2a**. In this case, in addition, significant differences were found between the three cell lines. Its interaction with the cell membrane (NRU method) promoted significant differences for compound **2a** between the A431 and HeLa and MCF-7 cell lines.

From the results reported above, it can be rationalized that the cytotoxic response and selective toxicity, in addition to the cell line, depend on the nature of the substituent on the nitrogen atom in the lactam moiety. When evaluating γ-lactams **2a**, **2b**, **2d** and **2p**, optimal outcomes in terms of selective toxicity were observed with the *t*-butyl derivative **2a**. These findings are consistent with our previous research on β-lactam derivatives [24], where the best results regarding the selectivity index against the A431 cell line were achieved from β-lactam **A,** also with a *t*-butyl group on its nitrogen. Although the presence of this group in δ-lactam **2i** conferred significant differences to the promoted response of γ-lactam, its behavior is similar (SI values > 1) when compared to that of the observed β-lactam derivative. It is worth noting that the presence of a phenyl group on the nitrogen (compound **2d**), seems to provide even better results. From this study, it can be inferred that the synthesized γ-lactams, particularly compounds **2a** and **2d**, emerge as new molecules possessing notable antitumoral properties against the A431 squamous carcinoma cell line.

## 3. Materials and Methods

### 3.1. Materials and Methods for the Synthesis of Lactams

#### 3.1.1. General Experimental Procedures

NMR spectra were recorded on Bruker 400 and Bruker 500 spectrometers (Bruker BioSpin GmbH, Ettlingen, Germany) in CDCl_3_. Chemical shifts are reported as δ values (ppm) relative to internal Me_4_Si, and ^13^C NMR spectra are referenced in terms of the deuterated solvent signal (CDCl_3_: 77.00 ppm). All NMR data assignments are supported by COSY and HSQC experiments. The following abbreviations (or combinations) were used to describe ^1^H-NMR multiplicities: s = singlet, d = doublet, t = triplet, q = quartet, m = multiplet and b = broad. Infrared spectra were recorded on a Nicolet 320 FT-IR spectrophotometer. Melting points were recorded on a Gallenkamp melting point apparatus. High-resolution ESI mass spectra were obtained from an Agilent LC/MSD-TOF mass spectrometer. Analytical thin-layer chromatography was performed on SiO_2_ (Merck silica gel 60 F254, Darmstadt, Germany) with a fluorescent indicator (λ = 254 nm), and the spots were located by UV light and/or with 1% aqueous KMnO_4_ solution or anisaldehyde. Chromatography refers to flash chromatography and was carried out on SiO_2_ (Carlo Erba silica gel 60A, 35–70 μm). The drying of organic extracts during the workup of reactions was performed over anhydrous Na_2_SO_4_. Solvent evaporation was accomplished using a rotatory evaporator. All yields refer to chromatographically and spectroscopically (NMR) pure material. Microwave irradiation experiments were performed using a single-mode Discover System from the CEM Corporation (Matthews, NC, USA) using a standard Pyrex vessel (capacity 10 mL).

#### 3.1.2. Synthesis of Trichloro and Dichloroacetamides **1**

The synthesis and characterization of trichloroacetamides **1a**–**1i** and **1l**–**1m** were reported in our previous work [31]. Compound **1j** was prepared from its corresponding secondary amine [40] and trichloroacetyl chloride using the conditions outlined in our previous work [31]. The preparation and characterization of **1k** were reported previously [41].

*N*-Benzyl-2,2,2-trichloro-*N*-(1-phenylbut-3-en-1-yl)acetamide (**1j**).

Physical State: colorless oil.

^1^H NMR (400 MHz, CDCl_3_) δ 7.61–6.94 (m, 10H), 5.91 and 5.68 (2 br s, 1H), 4.12 and 5.12–4.64 (m, 5H), 3.02–2.64 (m, 2H); ^13^C NMR (101 MHz, CDCl_3_) δ 160.8 (C=O), 137.8, 137.0, 135.6 (C), 134.6, 133.7 (CH), 128.7, 128.4, 128.2, 127.9, 127.2 (Ar-CH), 117.9 (CH_2_), 93.9 (CCl_3_), 63.8 and 60.9 (CH), 53.1 and 49.5 (CH_2_), 36.1 (CH_2_). HRMS (ESI-TOF) calcd. for C_19_H_19_Cl_3_NO [M+H]^+^ 382.0527, found 382.0530.

#### 3.1.3. General Procedure for the ATRC Reactions of Trichloro- and Dichloroacetamides **1** in the Presence of RuCl_2_(PPh_3_)_3_

In a 10 mL vessel was placed trichloro or dichloroacetamide **1** (0.386 mmol) and RuCl_2_(PPh_3_)_3_ (18.5 mg, 0.019 mmol, 5 mol%) in toluene (1 mL). The mixture was heated while stirring using microwave irradiation for 15–45 min at 160 °C. After chromatography, (hexane-CH_2_Cl_2_, 9:1 to CH_2_Cl_2_) lactams **2** were isolated.

1-(*tert-*Butyl)-3,3-dichloro-4-(chloromethyl)pyrrolidin-2-one (**2a**).

Physical state: white solid, m.p. 70–73 °C.

^1^H NMR (400 MHz, CDCl_3_) δ 3.99 (dd, *J* = 11.3, 4.2 Hz, 1H, CH_2_Cl), 3.73 (br d, *J* = 10.1, Hz, 1H, H-5), 3.71 (dd, *J* = 10.2, 5.2 Hz, 1H, CH_2_Cl), 3.22 (dd, *J* = 10.1, 8.2 Hz, 1H, H-5), 2.99 (dddd, *J* = 10.2, 8.2, 6.9, 4.2 Hz, 1H, H-4), 1.44 (s, 9H, *t-*Bu); ^13^C NMR (101 MHz, CDCl_3_) δ 165.5 (C-2), 85.0 (C-3), 55.6 (C), 51.0 (C-4), 46.0 (C-5), 41.2 (CH_2_Cl), 27.2 (CH_3,_ *t-*Bu)); IR (NaCl) 3054, 2983, 2935, 1724 cm^−1^. HRMS (ESI-TOF) calcd. for C_9_H_15_Cl_3_NO [M+H]^+^ 258.0214, found 258.0205; calcd. for C_9_H_14_Cl_3_NNaO [M+Na]^+^ 280.0033, found 280.0019.

3,3-Dichloro-4-(chloromethyl)pyrrolidin-2-one (**2b**).

Physical state: white solid, m.p. 101–103 °C.

^1^H NMR (CDCl_3_, 400 MHz) δ 7.18 (br s, 1H, NH), 4.00 (dd, *J* = 11.3, 4.4 Hz, 1H, CH_2_Cl), 3.75 (dd, *J* = 11.3, 10.0 Hz, 1H, CH_2_Cl), 3.71 (ddd, *J* = 10.1, 6.8, 3.3 Hz, 1H, H-5), 3.28 (dd, *J* = 10.1, 8.3 Hz, 1H, H-5), 3.19 (dddd, *J* = 10.1, 8.3, 6.8, 4.4 Hz, 1H, H-4); ^13^C NMR (CDCl_3_, 101 MHz) δ 168.9 (CO), 82.9 (C-3), 53.6 (C-4), 43.5 (C-5), 40.9 (CH_2_Cl); IR (NaCl, neat) 3261, 3006, 2961, 2924, 2854, 1734 cm^−1^. HRMS (ESI-TOF) *m*/*z*: calcd for C_5_H_7_Cl_3_NO [M+H]^+^ 201.9588, found 201.9590; calcd for C_5_H_6_Cl_3_NNaO [M+Na]^+^ 223.9407, found 223.9407.

1-Butyl-3,3-dichloro-4-(chloromethyl)pyrrolidin-2-one (**2c**).

Physical state: amorphous colorless solid.

^1^H NMR (CDCl_3_, 400 MHz) δ 4.01 (dd, *J* = 11.2, 4.2 Hz, 1H, CH_2_Cl), 3.74 (dd, *J* = 11.2, 10.3 Hz, 1H, CH_2_Cl), 3.60 (dd, *J* = 10.1, 6.9 Hz, 1H, H-5), 3.47 (dt, *J* = 13.7, 7.5 Hz, 1H, CH_2_N), 3.30 (dd, *J* = 13.7, 6.9 Hz, 1H, CH_2_N), 3.26 (dd, *J* = 10.1, 8.2 Hz, 1H, H-5), 3.14–305 (m, 1H, H-4), 1.61–1.51 (m, 2H), 1.39–1.27 (m, 2H), 0.95 (t, *J* = 7.3 Hz, 3H, CH_3_); ^13^C NMR (CDCl_3_, 101 MHz) δ 165.9 (CO), 83.8 (C-3), 51.7 (C-4), 47.8 (C-5), 43.7 (CH_2_), 41.2 (CH_2_Cl), 28.9 (CH_2_), 19.8 (CH_2_), 13.6 (CH_3_); IR (NaCl, neat) 2960, 2932, 2872, 1727 cm^−1^. HRMS (ESI-TOF) *m*/*z*: calcd for C_9_H_15_Cl_3_NO [M+H]^+^ 258.0214, found 258.0214; calcd for C_9_H_14_Cl_3_NNaO [M+Na]^+^ 280.0033, found 280.0034.

3,3-Dichloro-4-(chloromethyl)-1-phenylpyrrolidin-2-one (**2d**).

Physical state: white solid, m.p. 127–130 °C.

^1^H NMR (CDCl_3_, 400 MHz) δ 7.63 (m, 2H, ArH), 7.43 (m, 2H, ArH), 7.26 (m, 1H, ArH), 4.12–4.06 (m, 2H), 3.84 (dd, *J* = 11.3, 10.3 Hz, 1H), 3.74 (dd, *J* = 10.0, 8.4 Hz, 1H), 3.30–3.21 (m, 1H); ^13^C NMR (CDCl_3_, 101 MHz) δ 164.4 (CO), 137.9 (*ipso*-C), 129.2, 126.2, 120.2 (ArCH), 84.0 (C-3), 51.1 (CH), 49.1 (CH_2_), 41.0 (CH_2_); IR (NaCl, neat) 3061, 2925, 2855, 1708, 1594 cm^−1^. HRMS (ESI-TOF) *m*/*z*: calcd for C_11_H_11_Cl_3_NO [M+H]^+^ 277.9901, found 277.9899; calcd for C_11_H_10_Cl_3_NNaO [M+Na]^+^ 299.9720; found 299.9720.

1-Benzyl-3,3-dichloro-4-(chloromethyl)pyrrolidin-2-one (**2e**).

Physical state: amorphous pale solid.

^1^H NMR (CDCl_3_, 400 MHz) δ 7.40–7.32 (m, 3H, ArH), 7.26–7.21 (m, 2H, ArH), 4.63 (d, *J* = 14.7 Hz, 1H, CH_2_Ar), 4.45 (d, *J* = 14.7 Hz, 1H, CH_2_Ar), 3.97 (dd, *J* = 11.3, 3.9 Hz, 1H), 3.67 (dd, *J* = 11.3, 9.7 Hz,1H), 3.51–3.41 (m, 1H), 3.12–3.02 (m, 2H); ^13^C NMR (CDCl_3_, 101 MHz) δ 166.0 (CO), 134.5 (*ipso*-C), 129.0, 128.3, 128.2 (ArCH), 51.6 (CH), 47.8 (CH_2_), 47.2 (CH_2_), 41.0 (CH_2_); IR (NaCl, neat) 3063, 3030, 2926, 2878, 1731, 1604, 1585 cm^−1^. HRMS (ESI-TOF) *m*/*z*: calcd for C_12_H_13_Cl_3_NO [M+H]^+^ 292.0057, found 292.0062; calcd for C_12_H_12_Cl_3_NNaO [M+Na]^+^ 313.9877, found 313.9877.

1-Allyl-3,3-dichloro-4-(chloromethyl)pyrrolidin-2-one (**2f**).

Physical state: amorphous white solid.

^1^H NMR (CDCl_3_, 400 MHz) δ 5.74 (ddt, *J* = 16.9, 10.2, 6.2 Hz, 1H, CH=), 5.32–5.24 (m, 2H, =CH_2_), 4.05–3.91 (m, 3H), 3.74 (dd, *J* = 11.2, 10.1 Hz, 1H), 3.59 (dd, *J* = 10.2, 7.0 Hz, 1H), 3.23 (dd, *J* = 10.2, 8.2 Hz, 1H), 3.15–3.06 (m, 1H); ^13^C NMR (CDCl_3_, 101 MHz) δ 165.7 (CO), 130.4 (CH), 119.7 (CH_2_), 83.6 (C-3), 51.7 (CH), 47.4 (CH_2_), 46.4 (CH_2_), 41.1 (CH_2_); IR (NaCl, neat) 3085, 3015, 2961, 2923, 1731, 1644 cm^−1^. HRMS (ESI-TOF) *m*/*z*: calcd for C_8_H_11_Cl_3_NO [M+H]^+^ 241.9901, found 241.9898; calcd for C_8_H_14_Cl_3_N_2_O [M+NH_4_]^+^ 259.0166, found 259.0164.

(*RS*)-1-(*tert*-Butyl)-3,3-dichloro-4-((*SR*)-1-chloroethyl)pyrrolidin-2-one (**2g**).

Physical state: amorphous white solid.

^1^H NMR (CDCl_3_, 400 MHz) δ 4.33 (dq, *J* = 9.9, 6.5 Hz, 1H, CHCl); 3.74 (dd, *J* = 10.3, 7.2 Hz, 1H, H-5), 3.17 (dd, *J* = 10.3, 9.0, 1H, H-5), 2.81 (ddd, *J* = 9.9, 9.0, 7.2 Hz, 1H, H-4), 1.84 (d, *J* = 6.5 Hz, 3H, CH_3_), 1.44 (s, 9H, *t*-Bu); ^13^C NMR (CDCl_3_, 101 MHz) δ 165.7 (CO), 84.8 (C-3), 56.9 (CHCl), 55.6 (C), 55.5 (C-4), 47.0 (C-5), 27.2 (CH_3_, *t*-Bu), 23.8 (CH_3_); IR (NaCl, neat) 2979, 2935, 2913, 2873, 1724 cm^−1^. HRMS (ESI-TOF) *m*/*z*: calcd for C_10_H_17_Cl_3_NO [M+H]^+^ 272.0370, found 272.0363; calcd for C_10_H_20_Cl_3_N_2_O [M+NH_4_]^+^ 289.0636, found 289.0627.

(*RS*)-1-(*tert*-Butyl)-3,3-dichloro-4-((*RS*)-1-chloroethyl)pyrrolidin-2-one (*epi-***2g**).

Physical state: white solid m.p. 113–114 °C.

^1^H NMR (CDCl_3_, 400 MHz) δ 4.34 (dq, *J* = 7.4, 6.7 Hz, 1H, CHCl); 3.55 (dd, *J* = 10.0, 7.0 Hz, 1H, H-5), 3.21 (dd, *J* = 10.0, 6.9, 1H, H-5), 2.98 (q, *J* = 7.1 Hz, 1H, H-4), 1.53 (d, *J* = 6.7 Hz, 3H, CH_3_), 1.44 (s, 9H, *t*-Bu); ^13^C NMR (CDCl_3_, 101 MHz) δ 165.9 (CO), 85.4 (C-3), 55.6 (C), 54.7 (C-4), 54.2 (CHCl), 44.4 (C-5), 27.2 (CH_3_, *t*-Bu), 21.9 (CH_3_); IR (NaCl, neat) 2981, 2934, 2914, 1715 cm^−1^. HRMS (ESI-TOF) *m*/*z*: calcd for C_10_H_17_Cl_3_NO [M+H]^+^ 272.0370, found 272.0366; calcd for C_10_H_20_Cl_3_N_2_O [M+NH_4_]^+^ 289.0636, found 289.0630.

(3a*RS*,4*RS*,7a*RS*)-1-Benzyl-3,3,4-trichlorohexahydro-*2H*-indole-2,5(*3H*)-dione (**2h**).

Physical state: colorless oil.

^1^H NMR (CDCl_3_, 400 MHz) δ 7.41–7.32 (m, 3H, ArH), 7.29–7.23 (m, 2H, ArH), 4.92 (d, *J* = 15.0 Hz, 1H, CH_2_Ar), 4.90 (d, *J* = 5.0 Hz, 1H, H-4), 4.26 (d, *J* = 15.0 Hz, 1H, CH_2_Ar), 3.82 (ddd, *J* = 11.3, 8.2, 5.1 Hz, 1H, H-7a), 3.46 (dd, *J* = 8.2, 5.0 Hz, 1H, H-3a), 2.70 (ddd, *J* = 17.0, 13.0, 6.0 Hz, H-6ax), 2.46 (dt, *J* = 17.0, 4.2 Hz, H-6eq), 2.27–2.18 (m, 1H, H-7eq), 2.15–2.02 (m, 1H, H-7ax); ^13^C NMR (CDCl_3_, 101 MHz) δ 199.2 (CO), 165.4 (CO), 134.4 (*ipso*-C), 129.2, 128.5, 128.1 (ArCH), 82.3 (C-3), 57.3 (C-3a), 57.2 (C-4), 52.2 (C-7a), 46.2 (CH_2_Ar), 32.8 (C-7), 24.6 (C-6); IR (NaCl, neat) 3064, 3029, 2949, 2923, 1737, 1715 cm^−1^. HRMS (ESI-TOF) *m*/*z*: calcd for C_15_H_15_Cl_3_NO_2_ [M+H]^+^ 346.0163, found 346.0158; calcd for C_15_H_18_Cl_3_N_2_O_2_ [M+NH_4_]^+^ 363.0428, found 363.0419.

1-(*tert*-Butyl)-3,3-dichloro-4-(chloromethyl)piperidin-2-one (**2i**).

Physical state: amorphous white solid.

^1^H NMR (CDCl_3_, 400 MHz) δ 4.13 (dd, *J* = 11.1, 2.9 Hz, 1H, CH_2_Cl), 3.57 (t, *J* = 11.1 Hz, 1H, CH_2_Cl), 3.58-3.51 (m, 1H, H-6), 3.31 (td, *J* = 12.1, 4.8 Hz, 1H, H-6), 2.66 (ddt, *J* = 12.1, 10.2, 3.0 Hz, 1H, H-4), 2.38 (ddt, *J* = 14.2, 4.9, 2.44 Hz, H-5), 1.88 (dtd, *J* = 14.2, 12.1, 5.7 Hz, H-5), 1.46 (s, 9H, *t*-Bu); ^13^C NMR (CDCl_3_, 101 MHz) δ 163.2 (CO), 87.2 (C-3), 59.0 (C), 51.8 (C-4), 44.0 (CH_2_Cl), 43.0 (C-6), 27.6 (CH_3_), 23.4 (C-5); IR (NaCl, neat): 3008, 2967, 2931, 2870, 1672 cm^−1^. HRMS (ESI-TOF) *m*/*z*: calcd for C_10_H_17_Cl_3_NO [M+H]^+^ 272.0370, found 272.0363.

(4*RS*,6*RS*)-1-Benzyl-3,3-dichloro-4-(chloromethyl)-6-phenylpiperidin-2-one (*trans-***2j**, more polar).

^1^H NMR (CDCl_3_, 400 MHz) δ 7.46–7.27 (m, 6H, ArH), 7.21–7.09 (m, 4H, ArH), 5.59 (d, *J* = 14.8 Hz, 1H, CH_2_Ar), 4.63 (dd, *J* = 5.5, 2.6 Hz, 1H, H-6), 4.04 (dd, *J* = 11.1, 3.0 Hz, 1H, CH_2_Cl), 3.51 (d, *J* = 14.8 Hz, 1H, CH_2_Ar), 3.51 (dd, *J* = 11.1, 9.9 Hz, 1H, CH_2_Cl), 2.82–2.72 (ddt, *J* = 10.8, 9.9, 3.4 Hz, 1H, H-4), 2.33 (ddd, *J* = 14.2, 3.6, 2.6 Hz, 1H, H-5), 2.27 (ddd, *J* = 14.2, 11.8, 5.5 Hz, 1H, H-5); ^13^C NMR (CDCl_3_, 101 MHz) δ 164.2 (CO), 138.6 and 135.8 (ArC), 129.4, 128.9, 128.4, 128.0, 126.1 (ArCH), 85.4 (C-3), 58.1 (C-6), 49.7 (CH_2_Ar), 47.2 (C-4), 43.5 (CH_2_Cl), 31.0 (C-5); IR (NaCl, neat) 3086, 3063, 3029, 2966, 2931, 1681, 1603, 1585 cm^−1^. HRMS (ESI-TOF) *m*/*z*: calcd for C_19_H_19_Cl_3_NO [M+H]^+^ 382.0527, found 382.0515.

(4*RS*,6*SR*)-1-Benzyl-3,3-dichloro-4-(chloromethyl)-6-phenylpiperidin-2-one (*cis***-2j,** less polar).

^1^H NMR (CDCl_3_, 400 MHz) δ 7.46–7.26 (m, 6H, ArH), 7.17 (m, 2H), 7.01 (m, 2H), 5.35 (d, *J* = 14.6 Hz, 1H, CH_2_Ar), 4.33 (dd, *J* = 11.6, 5.8 Hz, 1H, H-6), 4.20 (dd, *J* = 11.2, 2.9 Hz, 1H, CH_2_Cl), 3.57 (d, *J* = 14.6 Hz, 1H, CH_2_Ar), 3.55 (t, *J* = 11.2 Hz, 1H, CH_2_Cl), 2.73 (ddt, *J* = 12.8, 10.0, 2.8 Hz, 1H, H-4), 2.60 (ddd, *J* = 14.6, 5.8, 2.9 Hz, 1H, H-5), 2.06 (ddd, *J* = 14.6, 12.8, 11.6 Hz, 1H, H-5); ^13^C NMR (CDCl_3_, 101 MHz) δ 164.1 (CO), 139.8 and 135.8 (ArC), 129.3, 128.8, 128.7, 128.4, 127.8, 127.1 (ArCH), 85.7 (C-3), 60.1 (C-6), 50.5 (C-4), 48.3 (CH_2_Ar), 43.9 (CH_2_Cl), 32.9 (C-5); IR (NaCl, neat) 3086, 3063, 3029, 2926, 2849, 1676, 1603 cm^−1^. HRMS (ESI-TOF) *m*/*z*: calcd for C_19_H_19_Cl_3_NO [M+H]^+^ 382.0527, found 382.0514.

(1*RS*,5*RS*,6*RS*)-2-Benzyl-4,4,6-trichloro-2-azabicyclo[3.3.1] nonan-3-one (**2k**).

Physical state: white solid, mp 116–118 °C.

^1^H NMR (CDCl_3_, 400 MHz) δ 7.24–7.40 (m, 5H, ArH), 5.31 (d, 1H, *J* = 15.2 Hz, CH_2_Ar), 4.96 (br s, 1H, H-6), 3.91 (d, 1H, *J* = 15.2 Hz, CH_2_Ar), 3.54 (br d, 1H, H-1), 3.04 (br d, 1H, *J* = 3.2 Hz, H-5), 2.41 (m, 2H, CH_2_-9), 1.84–1.97 (m, 3H, CH_2_-7 and H-8ax), 1.70 (m, 1H, H-8eq); ^13^C NMR (CDCl_3_, 101 MHz) δ 164.2 (C-3), 136.1 (ipso-C), 127.8, 127.9, 128.9 (Ar-CH), 85.4 (C-4), 57.6 (C-6), 51.8 (C-5), 51.5 (C-1), 49.3 (CH2Ar), 24.5 (C-9), 24.4 (C-7), 22.5 (C-8); IR (NaCl, neat) 3109, 3089, 3063, 3032, 2960, 2946, 2933, 2859, 1659 cm^−1^. HRMS (ESI-TOF) Calcd for C_15_H_17_Cl_3_NO [M+H]^+^ 332.0370, found 332.0371.

Lactams *trans*-**2l** and *cis-***2l.**

*trans*-**2l** (major isomer) ^1^H NMR (CDCl_3_, 400 MHz) δ 4.25 (d, *J* = 8.2 Hz, 1H, H-3), 3.76 (dd, *J* = 11.3, 4.5 Hz, 1H), 3.68 (dd, *J* = 11.3, 6.7 Hz, 1H), 3.70-3.62 (m, 2H), 3.31 (dd, *J* = 10.0, 7.3 Hz, 1H), 2.74 (m, 1H, H-4), 1.43 (s, 9H, *t-*Bu); ^13^C NMR (CDCl_3_, 101 MHz) δ 168.4 (CO), 58.1 (C-3), 55.1 (C), 45.9 (CH_2_), 44.3 (C-4), 43.5 (CH_2_), 27.4 (CH_3_).

*cis*-**2l** (minor isomer) δ 4.36 (d, *J* = 6.2 Hz, 1H, H-3), 3.79 (dd, *J* = 11.2, 6.7 Hz, 1H), 3.65–3.57 (m, 2H), 3.31 (dd, *J* = 10.1, 8.2 Hz, 1H), 2.80 (m, 1H, H-4), 1.42 (s, 9H, *t-*Bu); ^13^C NMR (CDCl_3_, 101 MHz) δ 169.2 (CO), 59.6 (C-3), 54.9 (C), 46.9 (CH_2_), 42.2 (CH_2_), 40.3 (C-4), 27.4 (CH_3_); IR (NaCl, neat) 2976, 2935, 2910, 2875, 1704, 1698 cm^−1^. HRMS (ESI-TOF) *m*/*z*: calcd for C_9_H_16_Cl_2_NO [M+H]^+^ 224.0603, found 224.0602.

(3*RS*,4*SR*)-1-(*tert*-Butyl)-3-chloro-4-(chloromethyl)-3-methylpyrrolidin-2-one *(cis-***2m,** major isomer, less polar).

^1^H NMR (CDCl_3_, 400 MHz) δ 3.82 (dd, *J* = 11.2, 5.1 Hz, 1H, CH_2_Cl), 3.69 (dd, *J* = 11.2, 9.3 Hz, CH_2_Cl), 3.63 (dd, *J* = 10.0, 6.9 Hz, 1H, H-5), 3.15 (dd, *J* = 10.0, 9.2 Hz, 1H, H-5), 2.45 (tdd, *J* = 9.2, 6.9, 5.1 Hz, 1H, H-4), 1.73 (s, 3H, CH_3_), 1.42 (s, 9H, *t-*Bu); ^13^C NMR (CDCl_3_, 101 MHz) δ 170.9 (CO), 70.6 (C-3), 54.8 (C), 47.2 (C-4), 46.5 (C-5), 42.4 (CH_2_Cl), 27.4 (CH_3_), 24.7 (CH_3_). IR (NaCl, neat) 2974, 2930, 2872, 1686 cm^−1^. HRMS (ESI-TOF) *m*/*z*: calcd for C_10_H_17_Cl_2_NO [M+H]^+^ 238.0760, found 238.0762.

(3*RS*,4*RS*)-1-(*tert*-Butyl)-3-chloro-4-(chloromethyl)-3-methylpyrrolidin-2-one (*trans*-**2m**, minor isomer, less polar).

^1^H NMR (CDCl_3_, 400 MHz) δ 3.76–3.71 (m, 2H, H-5 and CH_2_Cl), 3.41 (dd, *J* = 11.1, 9.9 Hz, CH_2_Cl), 3.31 (dd, *J* = 10.3, 4.4 Hz, 1H, H-5), 2.84 (m, 1H, H-4), 1.59 (s, 3H, CH_3_), 1.43 (s, 9H, *t-*Bu); ^13^C NMR (CDCl_3_, 101 MHz) δ 170.9 (CO), 69.3 (C-3), 54.8 (C), 47.5 (C-4), 45.8 (C-5), 42.5 (CH_2_Cl), 27.3 (CH_3_), 21.2 (CH_3_); IR (NaCl, neat) 2976, 2929, 1698 cm^−1^. HRMS (ESI-TOF) *m*/*z*: calcd for C_10_H_17_Cl_2_NO [M+H]^+^ 238.0760, found 238.0767.

(3*RS*,4*SR*)-1-(*tert*-Butyl)-3-chloro-4-(chloromethyl)-3-(ethoxycarbonyl)-pyrrolidin-2-one (*cis*-**2n**, less polar).

^1^H NMR (CDCl_3_, 400 MHz) δ 4.41–4.27 (m, 2H, CH_2_O), 3.74 (dd, *J* = 11.2, 7.0 Hz, 1H, CH_2_Cl), 3.68 (dd, *J* = 9.7, 7.1 Hz, 1H, H-5), 3.61 (dd, *J* = 11.2, 7.8 Hz, CH_2_Cl), 3.45–3.33 (m, 1H, H-4), 3.18 (t, *J* = 9.3 Hz, 1H, H-5), 1.42 (s, 9H, *t-*Bu), 1.35 (t, *J* = 7.1 Hz, 3H, CH_3_); ^13^C NMR (CDCl_3_, 101 MHz) δ 167.0 (CO), 166.2 (CO), 72.3 (C-3), 63.5 (CH_2_O), 55.4 (C), 46.3 (C-5), 44.1 (C-4), 41.5 (CH_2_Cl), 27.3 (CH_3_), 14.0 (CH_3_); IR (NaCl, neat) 2979, 1760, 1698 cm^−1^. HRMS (ESI-TOF) *m*/*z*: calcd for C_12_H_20_Cl_2_NO_3_ [M+H]^+^ 296.0815, found 296.070814.

(3*RS*,4*RS*)-1-(*tert*-Butyl)-3-chloro-4-(chloromethyl)-3-(ethoxycarbonyl)-pyrrolidin-2-one (*trans*-**2n**, more polar).

^1^H NMR (CDCl_3_, 400 MHz): δ 4.32–4.21 (m, 2H, CH_2_O), 3.84 (dd, *J* = 11.0, 4.5 Hz, 1H, CH_2_Cl), 3.75 (dd, *J* = 9.5, 7.7 Hz, 1H, H-5), 3.41 (t, *J* = 11.0 Hz, CH_2_Cl), 3.32 (t, *J* = 9.5 Hz, H-5), 3.06–2.97 (m, 1H, H-4) 1.45 (s, 9H, *t-*Bu), 1.32 (t, *J* = 7.2 Hz, 3H, CH_3_); ^13^C NMR (CDCl_3_, 101 MHz) δ 166.8 (CO), 165.4 (CO), 72.0 (C-3), 63.4 (CH_2_O), 55.6 (C), 49.0 (C-4), 47.2 (C-5), 41.2 (CH_2_Cl), 27.3 (CH_3_), 14.0 (CH_3_); IR (NaCl, neat) 2973, 2835, 2908, 2872, 1758, 1705 cm^−1^. HRMS (ESI-TOF) *m*/*z*: calcd for C_12_H_20_Cl_2_NO_3_ [M+H]^+^ 296.0815, found 296.070816.

(3*RS*,3a*RS*,4*SR*,7a*RS*)-1-(*tert*-Butyl)-3,4-dichlorooctahydro-2H-indol-2-one (**2o**).

Physical state: white solid m.p. 117–120 °C.

^1^H NMR (CDCl_3_, 400 MHz) δ 4.65 (br s, 1H, H-4), 4.27 (d, *J* = 12.2 Hz, 1H, H-3), 3.94 (dt, *J* = 11.9, 6.1 Hz, 1H, H-7a), 2.71 (dd, *J* = 12.2, 6.7 Hz, H-3a), 2.25–2.16 (m, 1H), 2.04–1.96 (m, 1H), 1.89–1.76 (m, 2H), 1.67–1.58 (m, 1H), 1.45 (s, 9H, *t*-Bu), 1.22–1.10 (m, 1H); ^13^C NMR (CDCl_3_, 101 MHz): δ 167.8 (CO), 58.2 (C-3), 56.7 (C-4), 55.2 (C), 52.6 (C-7a), 51.7 (C-3a), 31.7 (CH_2_), 28.5 (CH_2_), 28.0 (CH_3_), 17.1 (CH_2_). IR (NaCl, neat) 2949, 2869, 1704 cm^−1^. HRMS (ESI-TOF) *m*/*z*: calcd for C_12_H_20_Cl_2_NO [M+H]^+^ 264.0916, found 264.0918.

#### 3.1.4. Radical Trapping Experiment

In a 10 mL vessel were placed trichloroacetamide **1a** (100 mg, 0.386 mmol), RuCl_2_(PPh_3_)_3_ (18.5 mg, 0.019 mmol, 5 mol%) and TEMPO (60.4 mg, 0.386 mmol) in toluene (1 mL). The mixture was heated while stirring, using microwave irradiation, for 15 min at 160 °C. The mixture was then concentrated and purified by chromatography using a mixture of Hexane/EtOAc (1:0 to 1:1) as eluent to provide **3a** (20 mg, mg, 14%) and **1a** (50 mg, 50%). **3a**: ^1^H NMR (400 MHz, CDCl_3_) δ 4.21 (dd, *J* = 9.3, 5.1 Hz, 1H), 3.92 (dd, *J* = 9.3, 8.2 Hz, 1H), 3.58 (dd, *J* = 9.8, 6.8 Hz, 1H, H-5), 3.25 (dd, *J* = 9.8, 7.8 Hz, 1H, H-5), 2.92 (qd, *J* = 7.7, 5.1 Hz, 1H, H-4), 1.50-1.41 (m, 5H), 1.43 (s, 9H, *t*-Bu), 1.38–1.29 (m, 2H), 1.22 (s, 3H, CH_3_), 1.16 (s, 3H, CH_3_), 1.11 (s, 3H, CH_3_), 1.10 (s, 3H, CH_3_); ^13^C NMR (100 MHz, CDCl_3_) δ 166.2 (C-2), 85.4 (C-3), 73.9 (CH_2_O), 59.9 (C), 55.3 (C), 48.4 (C-4), 45.4 (C-5), 39.4 (CH_2_), 33.2 (CH_3_), 33.0 (CH_3_), 27.2 (CH_3_), 20.1 (CH_3_), 17.0 (CH_2_) ; IR (NaCl) 3054, 2977, 2934, 1720 cm^−1^; HRMS (ESI-TOF) calcd. for C_18_H_33_Cl_2_N_2_O_2_ 379.1914 [M+H]^+^, found 379.1907. Calcd. for C_18_H_32_Cl_2_N_2_NaO_2_ 401.1733 [M+Na]^+^, found 401.1729.

### 3.2. Materials and Methods for the Biological Assays

#### 3.2.1. Materials

Dulbecco’s modified Eagle’s medium (DMEM), fetal bovine serum (FBS), L-glutamine solution (200 mM), trypsin–EDTA solution (170,000 U/L trypsin and 0.2 g/L EDTA), penicillin–streptomycin solution (10,000 U/mL penicillin and 10 mg/mL streptomycin) and phosphate-buffered saline (PBS) were obtained from Lonza (Verviers, Belgium). 3-(4,5-Dimethyl-2-thiazolyl)-2,5-diphenyltetrazolium bromide (MTT) and neutral red dye (NR) were obtained from Sigma–Aldrich (St. Louis, MO, USA). The 75 cm^2^ flasks and 96-well plates were obtained from TPP (Trasadingen, Switzerland). All other reagents were of analytical grade.

#### 3.2.2. Methods

##### *In Vitro Assay Using Human Erythrocytes* 

Acquisition and Extraction of the Erythrocytes

Human blood samples were obtained from the Banc de Sang i Teixits de Barcelona (Spain) from the Catalan Department of Health. Blood was deposited in tubes with the anticoagulant EDTA-K3. Blood samples were centrifuged at 3000 rpm and 4 °C for 10 min (Megafuge 2.0 R. Heraeus Instruments, Hanau, Germany) to induce sedimentation. Plasma was extracted with a Pasteur pipette. Next, the residual pellet was washed with PBS at pH 7.4. This procedure was repeated three times to remove residual leukocytes and platelets and to concentrate the erythrocytes. Following the last wash, the erythrocyte suspension was diluted (1:1) in PBS at pH 7.4 to obtain a suitable erythrocyte suspension (cell density of 8 × 10^9^ cell/mL).

Hemolysis Assay

The hemolysis assay determined the capability of different compounds to induce the hemolysis of the erythrocyte membrane. Stock solutions of each compound at 1 mg/mL in PBS at pH 7.4 were prepared. Different volumes (10–80 μL) were placed in polystyrene tubes, and an aliquot of 25 µL of the erythrocyte suspensions was added to each tube. The final volume was 1 mL. The tubes were incubated at room temperature under rotatory conditions. Then, the tubes were centrifuged at 10,000 rpm for 5 min. The supernatants’ absorbance at 540 nm (Shimadzu UV-160A, Shimadzu, Duisburg, Germany) was compared with that of the positive (erythrocytes hemolyzed with distilled water) and negative (erythrocyte suspension in PBS at pH 7.4.) controls.

The degree of hemolysis was determined using the following equation:

Hemolysis (%) = 100 × (Abs − Abs_0_)/(Abs_100_ − Abs_0_)

where Abs, Abs_0_ and Abs_100_ are the absorbance of the test samples, of the suspension treated with isotonic phosphate–buffered saline (PBS) and of the suspension of complete hemolysis treated with distilled water, respectively.

##### *Cell Cultures* 

The murine Swiss albino fibroblasts (3T3), the human breast adenocarcinoma (MCF-7), the human epithelial carcinoma (HeLa) and the squamous cell carcinoma (A431) cell lines were obtained from Celltec UB. Cells were grown in DMEM medium (4.5 g/L glucose) supplemented with 10% (*v*/*v*) FBS, 2 mM L-glutamine, 100 U/mL penicillin and 100 µg/mL streptomycin at 37 °C, 5% CO_2_. Cells were routinely cultured in 75 cm^2^ culture flasks and were trypsinized using trypsin–EDTA when the cells reached approximately 80% confluence. The trypan blue assay, which allows for a direct identification and enumeration of the live (unstained) and dead (blue) cells in the cell population, was used to evaluate the viability of the cells in the cell suspension obtained.

##### *Cell Viability Assays* 

3T3 (1 × 10^5^ cells/mL), A431, HeLa and MCF-7 cells (5 × 10^4^ cells/mL) were grown at defined densities in the 60 central wells of a 96-well plate. Cells were incubated for 24 h in 5% CO_2_ at 37 °C. Then, the spent medium was removed, and cells were incubated for 24 h with their corresponding compound solutions (1 mg/mL) previously diluted in a minimum amount of DMF (dimethylformamide) and then in DMEM medium supplemented with 5% FBS (100 µL) at the required concentration range (10–250 µg/mL). The viability of the cells upon incubation with the lactam derivatives was assayed using 2 different endpoints: NRU and MTT.

NRU Assay

The neutral red uptake (NRU) assay is based on the accumulation of dye in the lysosomes of viable cells. After the cells were incubated for 24 h with the corresponding systems, the medium was removed, and the solutions were incubated with the NR dye (Sigma-Aldrich, St. Louis, MO, USA) solution (50 µg/mL) dissolved in the medium, without FBS and without phenol red (Lonza, Verviers, Belgium), for 3 h. Cells were then washed with sterile PBS, followed by the addition of 100 µL of a solution containing 50% absolute ethanol and 1% acetic acid in distilled water to extract the dye. To promote the total dissolution of the dye, plates were placed in a microtiter-plate shaker for 5 min at room temperature. The absorbance of the resulting solutions was measured at 550 nm (Bio-Rad 550 microplate reader, Bio-Rad California, Hercules, CA, USA). Finally, the effect of each treatment was calculated as the percentage of dye uptake by viable cells relative to the control cells (cells without any treatment).

MTT Assay

Only living cells can reduce the yellow tetrazolium salt 3-(4,5-dimethyl-2-thiazolyl)-2,5-diphenyltetrazolium bromide (MTT) to insoluble purple formazan crystals. After a 24 h incubation of the cells with their corresponding NPs, the medium was removed and 100 µL of MTT (Sigma-Aldrich, St. Louis, USA) in PBS (5 mg/mL) diluted 1:10 in culture medium without phenol red and without FBS (Lonza, Verviers, Belgium) was added to the cells. After 3 h of incubation, the medium was removed. Thereafter, 100 µL of DMSO (Sigma-Aldrich, St. Louis, MO, USA) was added to each well to dissolve the purple formazan crystals. Agitation and determination of the absorbance of the extracted solution were performed under the same conditions as described in NRU Assay section. Finally, the effect of each treatment was calculated as the percentage of tetrazolium salt’s reduction by viable cells relative to control cells (cells without any treatment).

##### *Selectivity towards Cancer Cells* 

The corresponding half-maximal inhibitory concentration (IC_50_) values for the different formulations as a function of the cell line and endpoint were determined from the fitting of concentration-dependent viability curves. The corresponding selectivity indexes toward tumoral cells were calculated using the following ratio:

SI = IC50 (non-tumoral cell line)/IC50 (tumoral cell line)

where 3T3 fibroblasts were used as close representative cells under non-tumoral conditions. Moreover, pseudo selectivity indexes toward tumoral cells were calculated considering the discrete cellular responses at the maximum tested concentration (250 µg/mL).

##### *Statistical Analyses* 

Experiments were performed three times on independent occasions unless otherwise stated. The results are expressed as means ± standard deviation. A one-way analysis of variance (ANOVA) was used to determine the statistical differences between data sets, followed by Scheffé post hoc tests for multiple comparisons. IBM SPSS Statistics software version 29.0 (New York, NY, USA) was used to execute statistical analyses. Differences were considered statistically significant at *p* < 0.001. Significant differences were illustrated in the figures using an asterisk or other superscript symbols.

## 4. Conclusions

In conclusion, even if ATRCs in the presence of RuCl_2_(PPh_3_)_3_ have already been reported in the literature from alkenyl-tethered trichloroacetamides, in this investigation we have showed that these reactions can be achieved under microwave activation. Hence, we were able to access several γ- and δ-lactams with good yields and within remarkably short reaction times. The process was successfully used to access the indole and the morphan scaffolds where a completely diastereoselective reaction takes place, generating three stereogenic centers. Moreover, the optimized conditions were efficiently extended to α,α-dichloroamides. Finally, four γ-lactams and one δ-lactam were evaluated for their hemolytic and cytotoxic properties. The results showed that these compounds have non-hemolytic properties and that their cytotoxic response is highly dependent on their structure and concentration. The γ-Lactam **2a** with a *t*-butyl group on its nitrogen was found to be the most cytotoxic, showing the lowest IC_50_ values (100–250 µg/mL) as a function of the cell line. Under the assayed conditions, **2a** revealed promising selectivity against squamous cell carcinoma (A431 cell line) in comparison with fibroblasts (3T3 cell line).

## Figures and Tables

**Table 1:** Screening of the ATRC reaction conditions for **1a** ^a^.

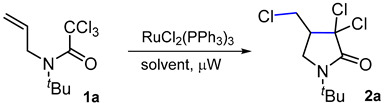						
Entry	RuCl_2_(PPh_3_)_3_ (mol%)	Solvent	Temp (°C)	Time (min)	2a (%)	1a (%)
1	5	PhMe	140	75	0	100
2	10	PhMe	160	30	79	traces
3	5	PhMe	160	30	82	Traces
4	5	PhMe	160	15	83	Traces
5	5	PhMe	160	10	79	Traces
6	2.5	PhMe	160	20	61	12
7	3	PhMe	160	60	71	5
8	5	ACN	160	15	0	100
9	5	PhMe	160 ^b^	15	30	60
10	5 ^c^	PhMe	160	15	35	42

^a^ Unless otherwise noted, the reactions were carried out with 0.386 mmol of **1a** in 1 mL of solvent under microwave activation. All products were separated through column chromatography and analyzed by NMR spectroscopy. ^b^ Reaction achieved under conventional heating. ^c^ In the presence of second-generation Grubbs catalyst.

**Table 2:** ATRC from trichloroacetamides **1b**–**1f** ^a^.

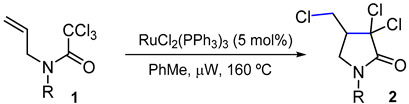						
Entry	R	1	Time (min)	2	Yield%	1 (Recovered %)
1	H	**1b**	15	**2b**	62	8
2	H	**1b**	30	**2b**	68	7
3	*n*Bu	**1c**	15	**2c**	90	0
4	Ph	**1d**	15	**2d**	48	45
5	Ph	**1d**	45	**2d**	84	13
6	Bn	**1e**	15	**2e**	78	traces
7	Allyl	**1f**	15	**2f**	78	traces

^a^ Unless otherwise noted, the reactions were carried out with 0.386 mmol of **1** in 1 mL of toluene under microwave irradiation. All products were separated through column chromatography and analyzed by NMR spectroscopy.

**Table 3:** Screening of the ATRC reaction conditions from **1l**, **1m** and **1n** ^a^.

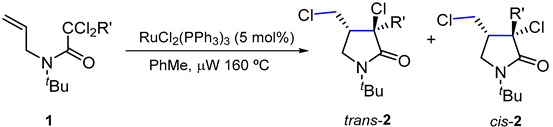						
Entry	R′	1	Time (min)	2	Yield %	*trans*/*cis*
1	H	**1l**	15	**2l**	75	*trans*-**2l**/*cis***-2l**: 1.8/1
2	Me	**1m**	30	**2m**	80	*trans***-2m**/*cis*-**2m**: 1/1.9
3	CO_2_Et	**1n**	15	**2n**	70	*trans*-**2n**/*cis***-2n**: 1/1

^a^ Unless otherwise noted, the reactions were carried out with 0.386 mmol of **1l**, **1m** or **1n** in 1 mL of toluene under microwave irradiation. All products were separated through column chromatography and analyzed by NMR spectroscopy.

**Table 4:** Cell viability assessment: half-maximal inhibitory concentration (IC_50_) values of the corresponding lactam derivatives as a function of their cell line and endpoint method. Selectivity index (SI) against tumoral cell lines in comparison with a non-tumoral cell line (3T3) was also included.

Compound	MTT IC_50_ (µg/mL)				NRU IC_50_ (µg/mL)				MTT SI			NRU SI		
	3T3	A431	Hela	MCF7	3T3	A431	HeLa	MCF7	A431	HeLa	MCF7	A431	HeLa	MCF7
**2a**	172	126	109	>250	>250	82	>250	>250	1.4	1.68	<0.69	>3.1	n.d.^a^	n.d.
**2b**	>250	>250	>250	>250	>250	>250	>250	>250	n.d.	n.d.	n.d.	n.d.	n.d.	n.d.
**2d**	>250	93	>250	>250	>250	>250	>250	>250	>2.7	n.d.	n.d.	n.d.	n.d.	n.d.
**2i**	>250	>250	>250	>250	>250	>250	>250	>250	n.d.	n.d.	n.d.	n.d.	n.d.	n.d.
**2p**	>250	>250	>250	>250	>250	>250	>250	>250	n.d.	n.d.	n.d.	n.d.	n.d.	n.d.

^a^ n.d. Not determined.

## Data Availability

Data are contained within the article and Supplementary Materials.

## Supplementary Materials

The following supporting information can be downloaded at https://www.mdpi.com/article/10.3390/molecules29092035/s1, Copies of ^1^H and ^13^C spectra of **1j**, **2a**-**2o** and **3a**.
