# Supplementary material for: Microwave-Assisted Atom Transfer Radical Cyclization in the Synthesis of 3,3-Dichloro-γ- and δ-Lactams from N-Alkenyl-Tethered Trichloroacetamides Catalyzed by RuCl2(PPh3)3 and Their Cytotoxic Evaluation
Source: Molecules. 2024 Apr 28;29(9):2035. doi: 10.3390/molecules29092035 (PMC11085086; doi:10.3390/molecules29092035)

Supplementary Material for

Microwave-Assisted Atom Transfer Radical Cyclization in the Synthesis of 3,3-Dichloro-γ- and δ-Lactams from *N*-Alkenyl-Tethered Trichloroacetamides Catalyzed by $RuCl_2(PPh_3)_3$ and their Cytotoxic Evaluation

Faïza Diaba[1,*], Alexandra G. Sandor[1], María del Carmen Morán[2,3]

[1] Laboratori de Química Orgànica, Facultat de Farmàcia i Ciències de l'Alimentació, IBUB, Universitat de Barcelona, Av. Joan XXIII 27-31, 08028 Barcelona, Spain
[2] Departament de Bioquímica i Fisiologia-Secció de Fisiologia, Facultat de Farmàcia i Ciències de l'Alimentació, Universitat de Barcelona, Avda. Joan XXIII 27-31, 08028 Barcelona, Spain
[3] Institut de Nanociència i Nanotecnologia—IN2UB, Universitat de Barcelona, Avda. Diagonal, 645, 08028 Barcelona, Spain

***** Correspondence: faiza.diaba@ub.edu; Tel.: 34934035849

- Copies of ^{1}H NMR and ^{13}C NMR spectra of **1j** S2
- Copies of ^{1}H NMR and ^{13}C NMR spectra of lactams **2a-2o** S3-S21
- Copies of ^{1}H NMR and ^{13}C NMR spectra of lactam **3a** S22

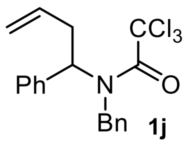

1j

VNMRS400A_17072020_XGT041-6-H1
VNMRS400F / Num.Inv. 205984
cdcl3 / Temp: 25C / N.Reg: XXXXXXXXXX
Usuari: san / Mostra: XGT041-6
Nom: FAIZA DIABA
Data: 17/07/20 / Ope.: F.DIABA

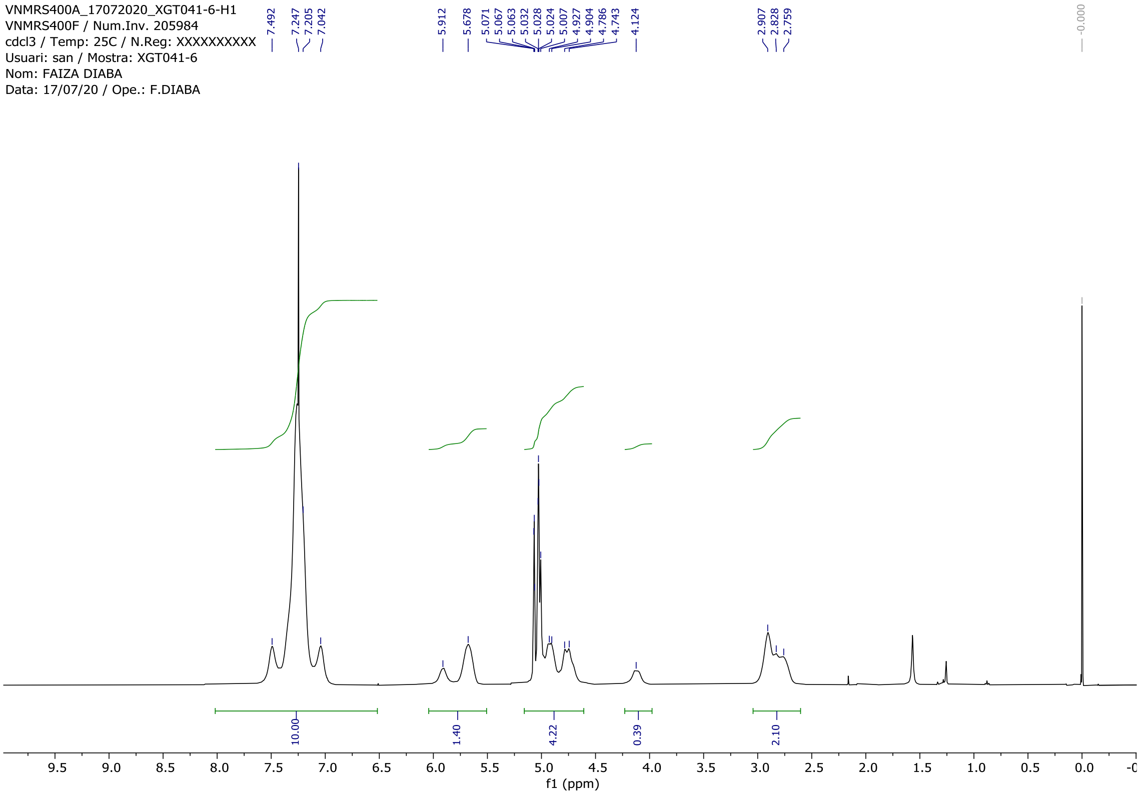

VNMRS400A_17072020_XGT041-6-C13
VNMRS400F / Num.Inv. 205984
cdcl3 / Temp: 25C / N.Reg: XXXXXXXXXX
Usuari: san / Mostra: XGT041-6
Nom: FAIZA DIABA
Data: 17/07/20 / Ope.: F.DIABA

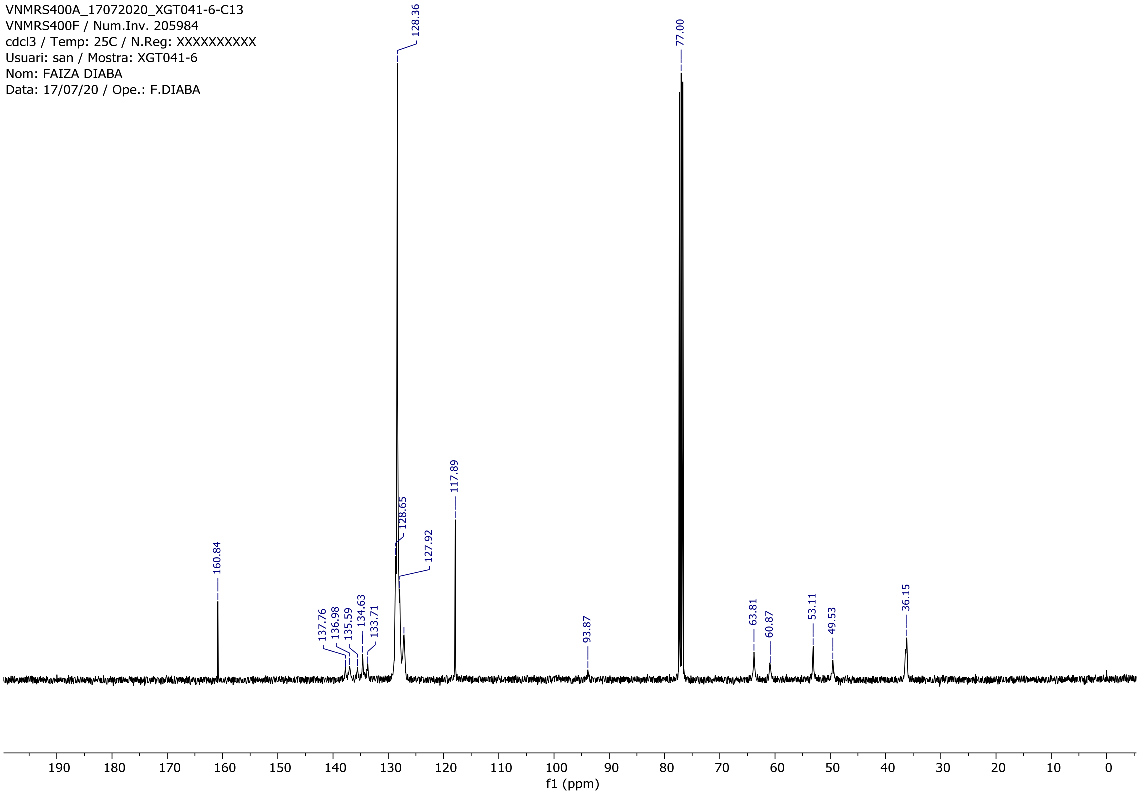

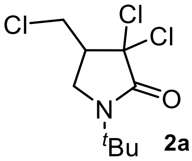
Cl
Cl
Cl
O
N
tBu

2a

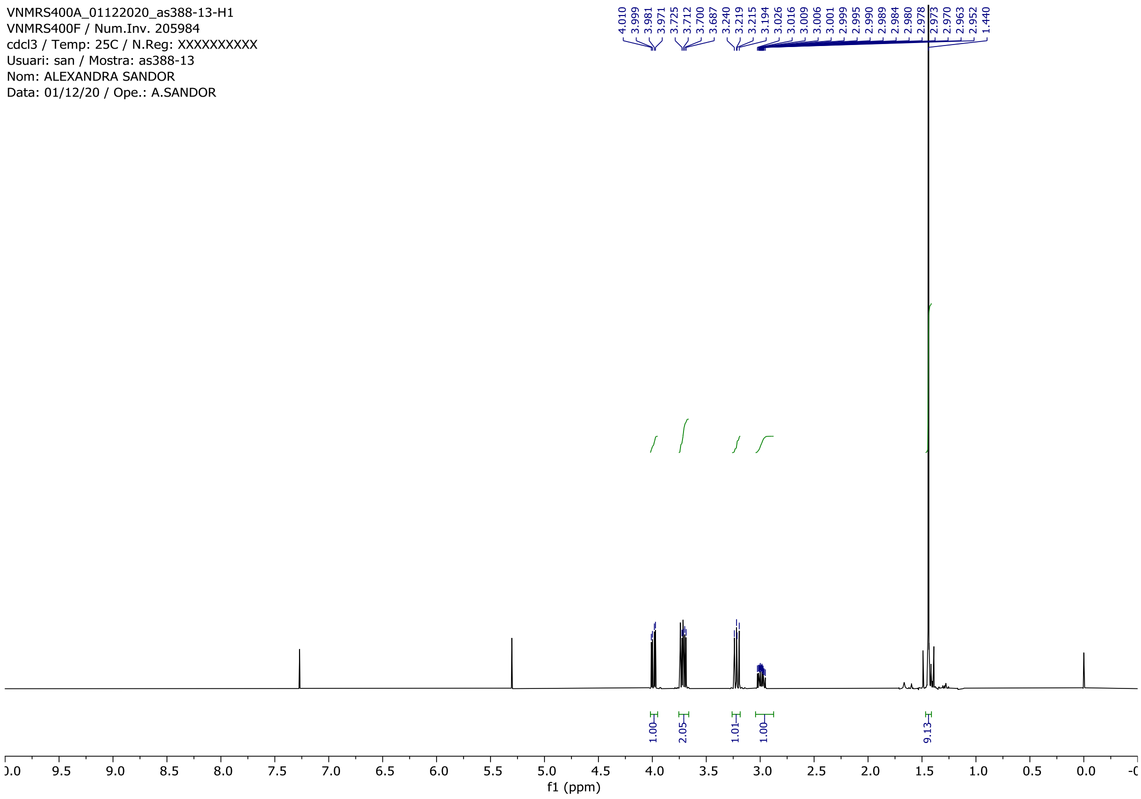
VNMRS400A_01122020_as388-13-H1
VNMRS400F / Num.Inv. 205984
cdcl3 / Temp: 25C / N.Reg: XXXXXXXXXX
Usuari: san / Mostra: as388-13
Nom: ALEXANDRA SANDOR
Data: 01/12/20 / Ope.: A.SANDOR
4.010
3.999
3.981
3.971
3.725
3.712
3.700
3.687
3.240
3.219
3.215
3.194
3.026
3.016
3.009
3.006
3.001
2.999
2.995
2.990
2.989
2.984
2.980
2.978
2.973
2.970
2.963
2.952
1.440
1.00
2.05
1.01
1.00
9.13
f1 (ppm)

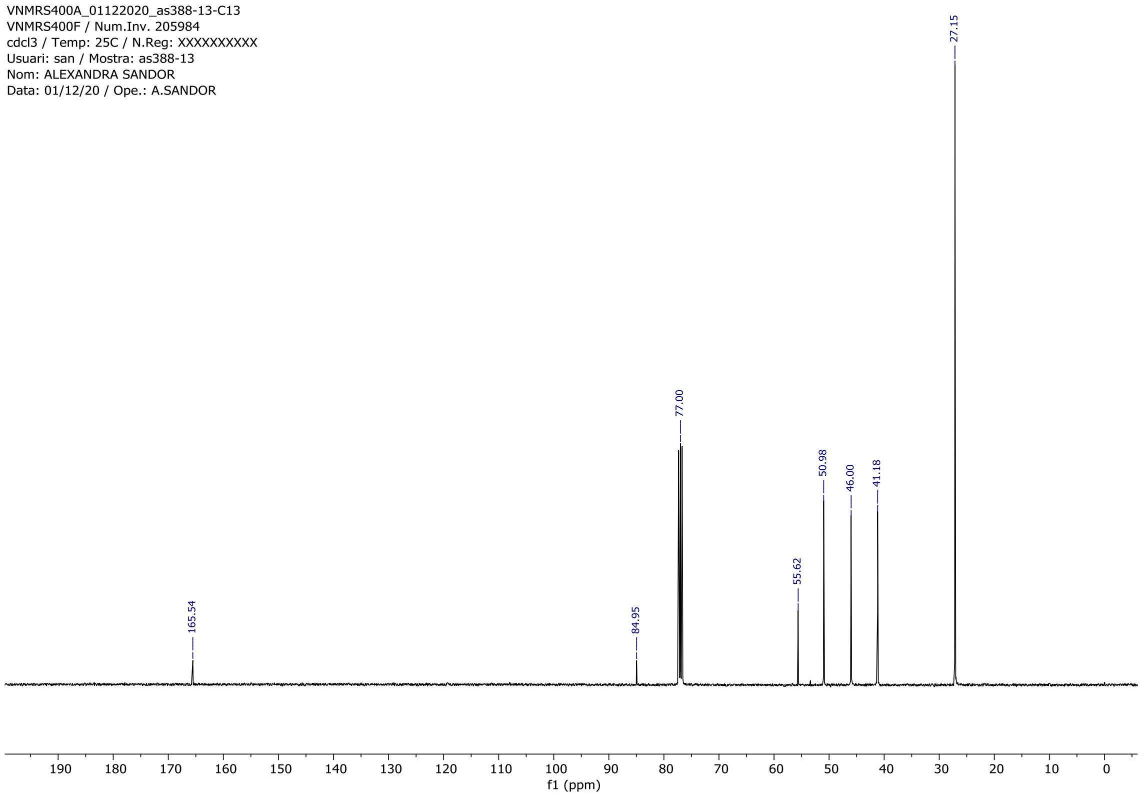
VNMRS400A_01122020_as388-13-C13
VNMRS400F / Num.Inv. 205984
cdcl3 / Temp: 25C / N.Reg: XXXXXXXXXX
Usuari: san / Mostra: as388-13
Nom: ALEXANDRA SANDOR
Data: 01/12/20 / Ope.: A.SANDOR
165.54
84.95
77.00
55.62
50.98
46.00
41.18
27.15
f1 (ppm)

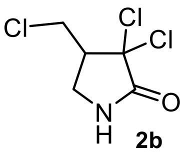

2b

23100167_B400FA_21102023_XAS416.1.fid
Equip: B400F / N.Inv: 1037597
N.Reg: 23100167
Usuari: san / Mostra: XAS416
Nom: FAIZA DIABA NOUARA
Data: 21/10/2023 09:25:31 h./ Ope.: AUTOSERVEI
Experiment: A-H1-zg30 Solvent: CDCl3

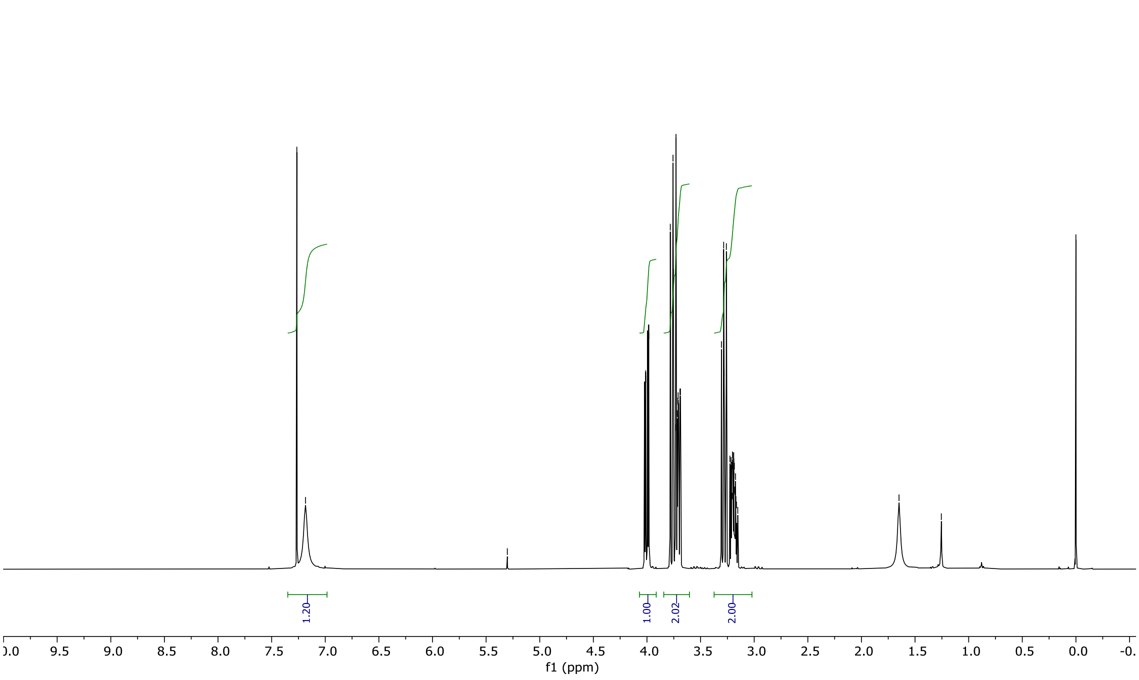

23100167_B400FA_21102023_XAS416.2.fid
Equip: B400F / N.Inv: 1037597
N.Reg: 23100167
Usuari: san / Mostra: XAS416
Nom: FAIZA DIABA NOUARA
Data: 21/10/2023 09:25:31 h./ Ope.: AUTOSERVEI
Experiment: A-C13-zgpg30 Solvent: CDCl3

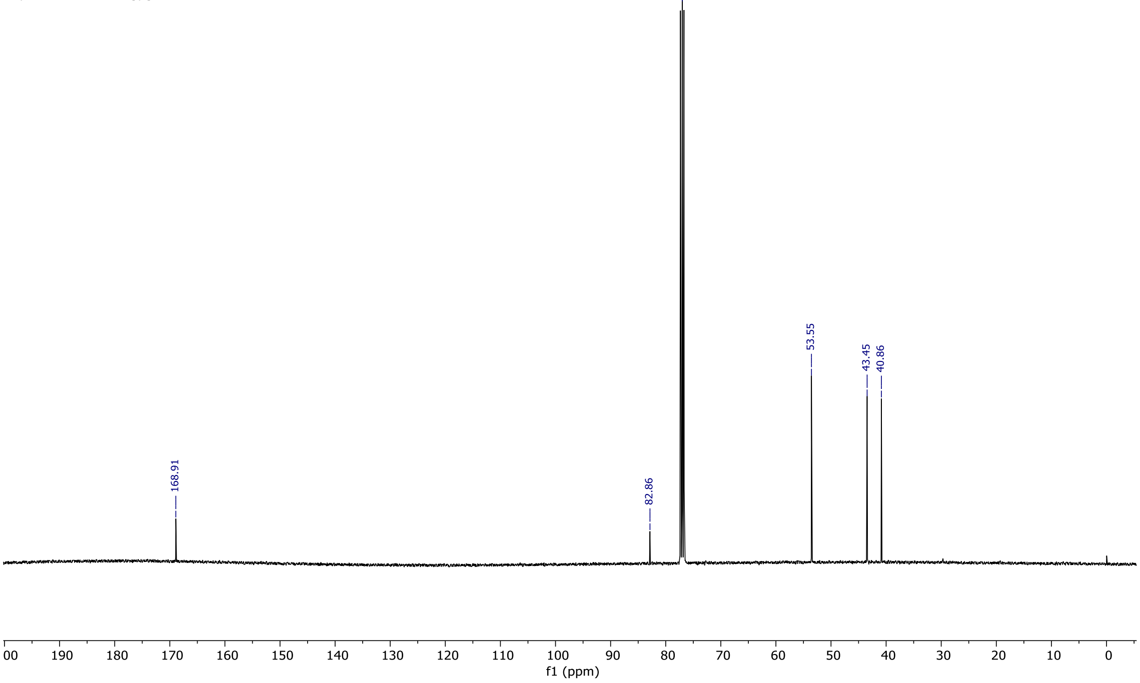

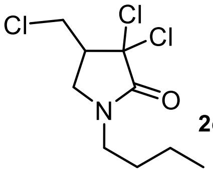

2c

VNMRS400A_27012021_AS414-14-H1
VNMRS400F / Num.Inv. 205984
cdcl3 / Temp: 25C / N.Reg: XXXXXXXXXX
Usuari: san / Mostra: AS414-14
Nom: ALEXANDRA SANDOR
Data: 27/01/21 / Ope.: A.SANDOR

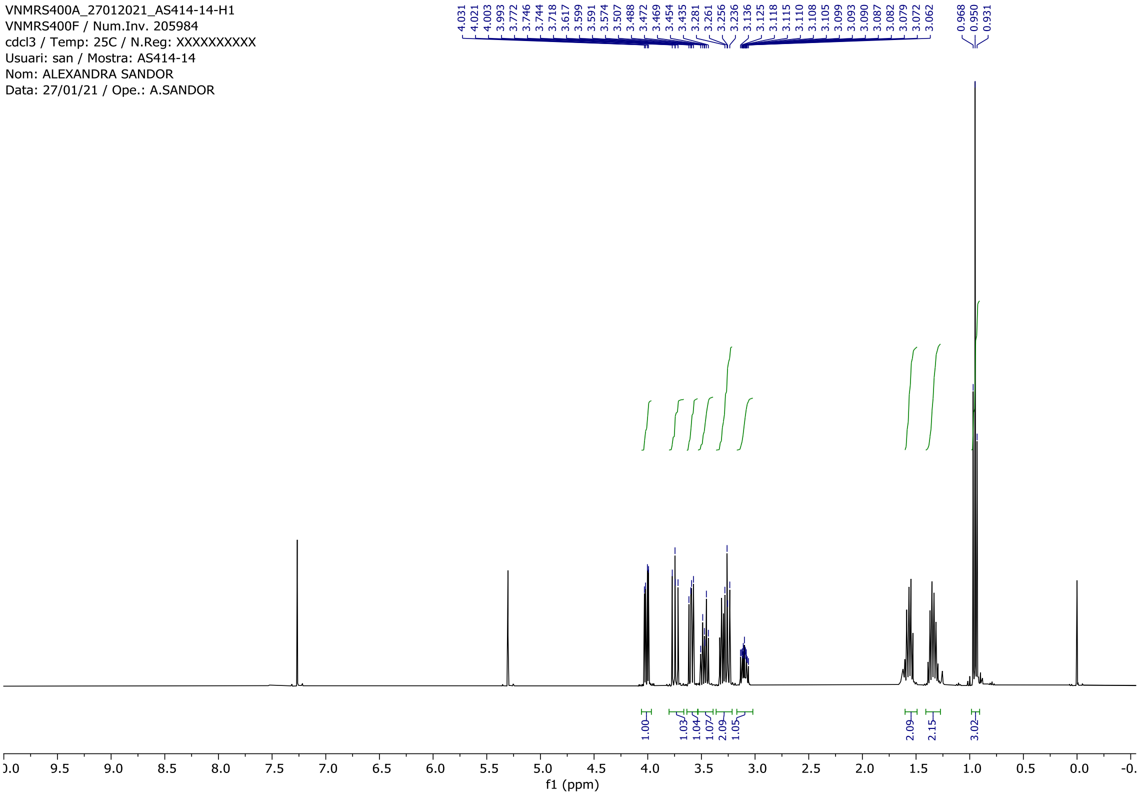

VNMRS400A_27012021_AS414-14-C13
VNMRS400F / Num.Inv. 205984
cdcl3 / Temp: 25C / N.Reg: XXXXXXXXXX
Usuari: san / Mostra: AS414-14
Nom: ALEXANDRA SANDOR
Data: 27/01/21 / Ope.: A.SANDOR

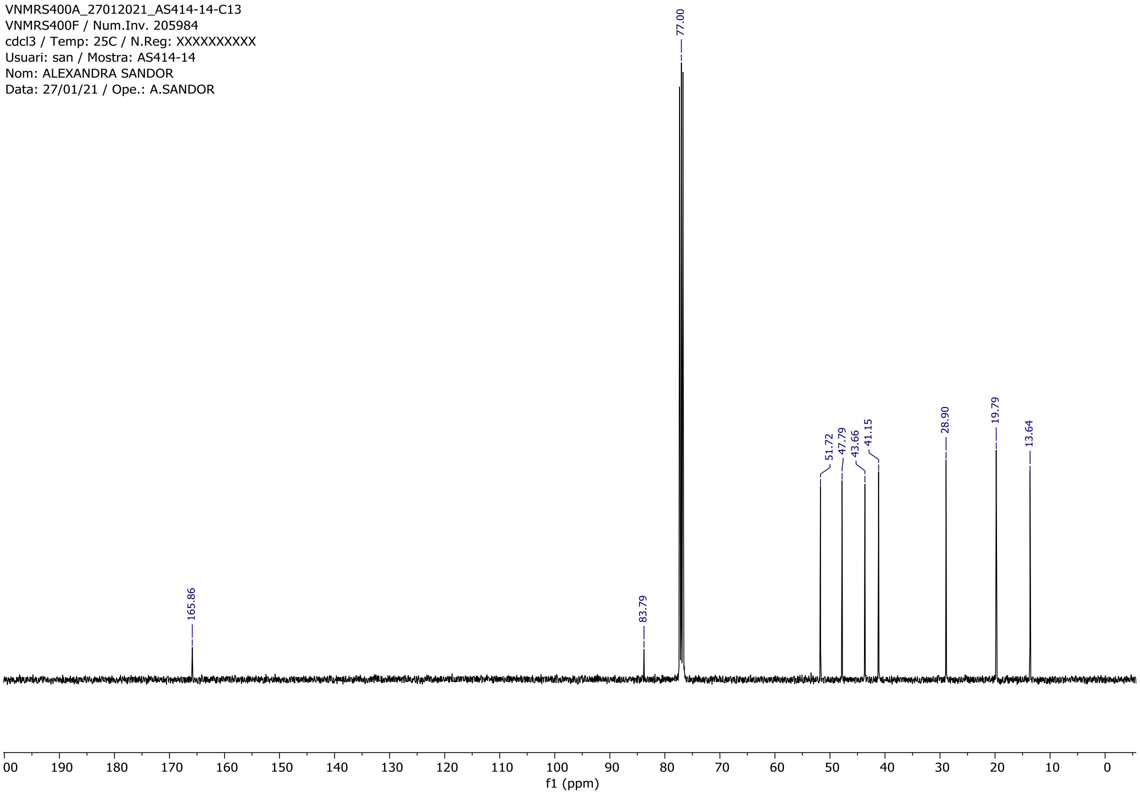

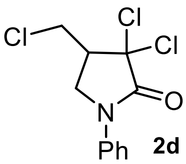

2d

VNMRS400A_26012021_AS413-28-H1
VNMRS400F / Num.Inv. 205984
cdcl3 / Temp: 25C / N.Reg: XXXXXXXXXX
Usuari: san / Mostra: AS413-28
Nom: ALEXANDRA SANDOR
Data: 26/01/21 / Ope.: A.SANDOR

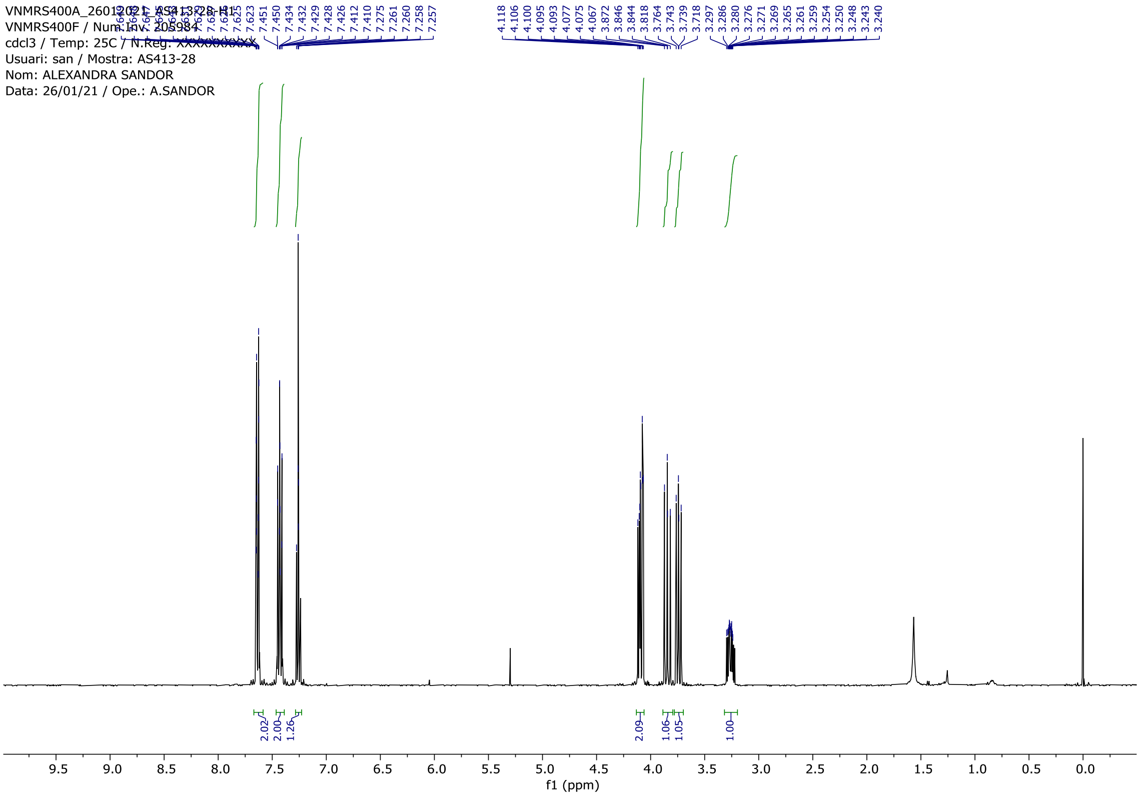

VNMRS400A_26012021_AS413-28-C13
VNMRS400F / Num.Inv. 205984
cdcl3 / Temp: 25C / N.Reg: XXXXXXXXXX
Usuari: san / Mostra: AS413-28
Nom: ALEXANDRA SANDOR
Data: 26/01/21 / Ope.: A.SANDOR

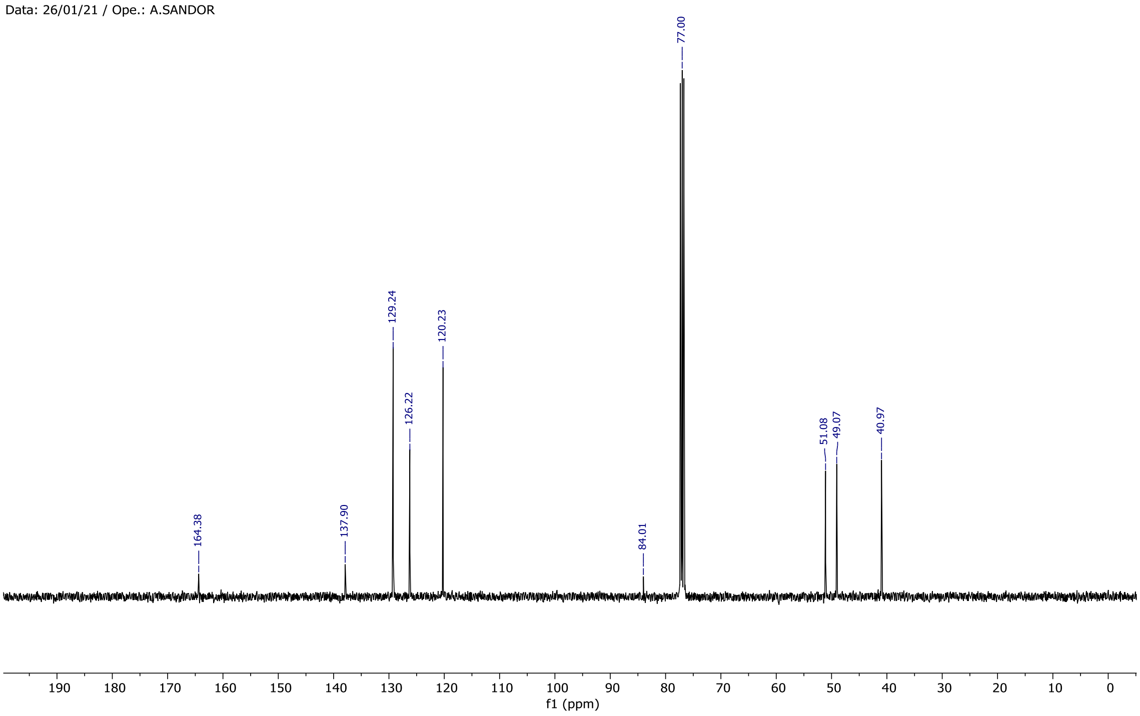

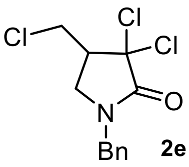

2e

VNMRS400A_04022021_as417-8-H1
VNMRS400F / Num.Inv. 205984
cdcl3 / Temp: 25C / N.Reg: XXXXXXXXXX
Usuari: san / Mostra: as417-8
Nom: ALEXANDRA SANDOR
Data: 04/02/21 / Ope.: A.SANDOR

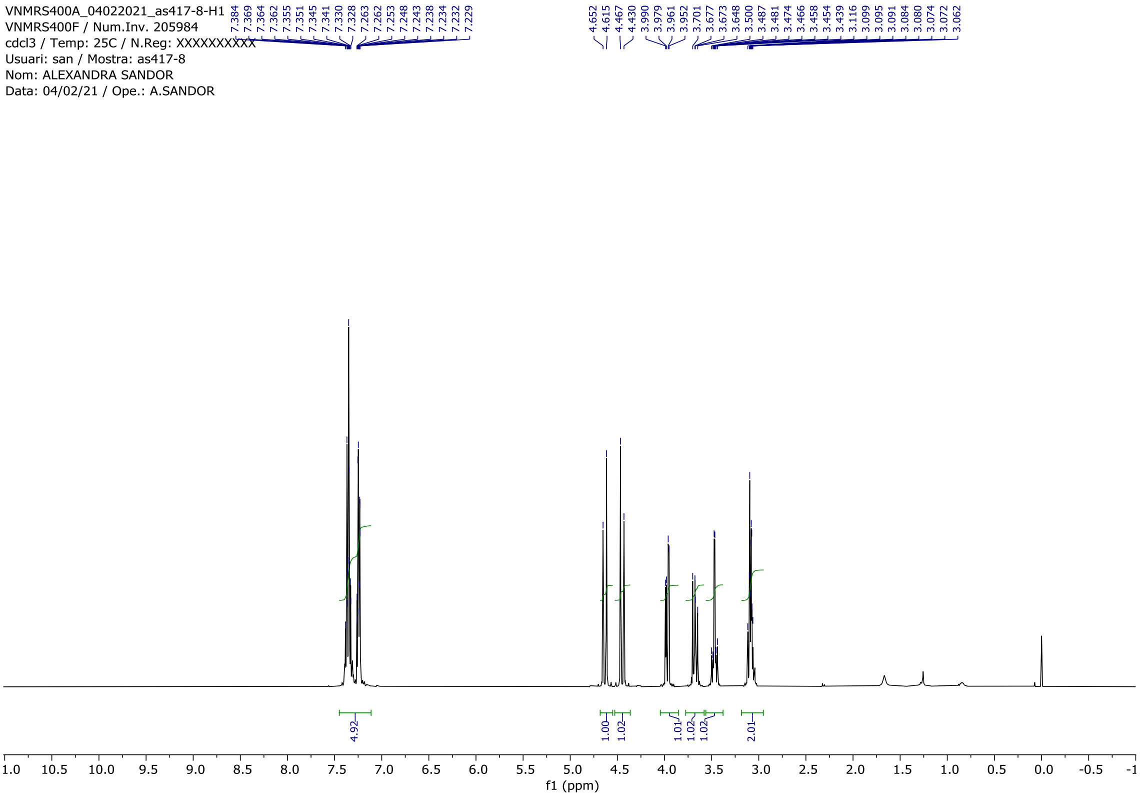

VNMRS400A_04022021_as417-8-C13
VNMRS400F / Num.Inv. 205984
cdcl3 / Temp: 25C / N.Reg: XXXXXXXXXX
Usuari: san / Mostra: as417-8
Nom: ALEXANDRA SANDOR
Data: 04/02/21 / Ope.: A.SANDOR

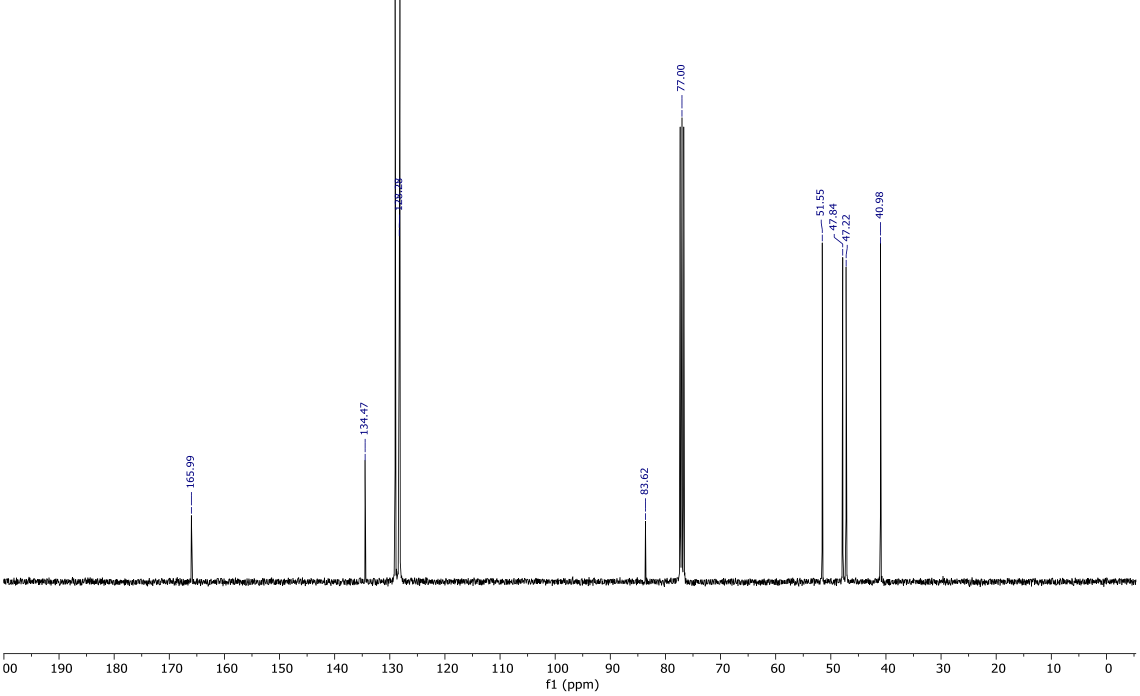

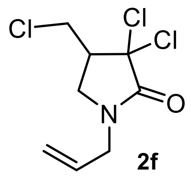

2f

VNMRS400A_18122020_as404-2-20-H1
VNMRS400F / Num.Inv. 205984
cdcl3 / Temp: 25C / N.Reg: XXXXXXXXXX
Usuari: san / Mostra: as404-2-20
Nom: ALEXANDRA SANDOR
Data: 18/12/20 / Ope.: A.SANDOR

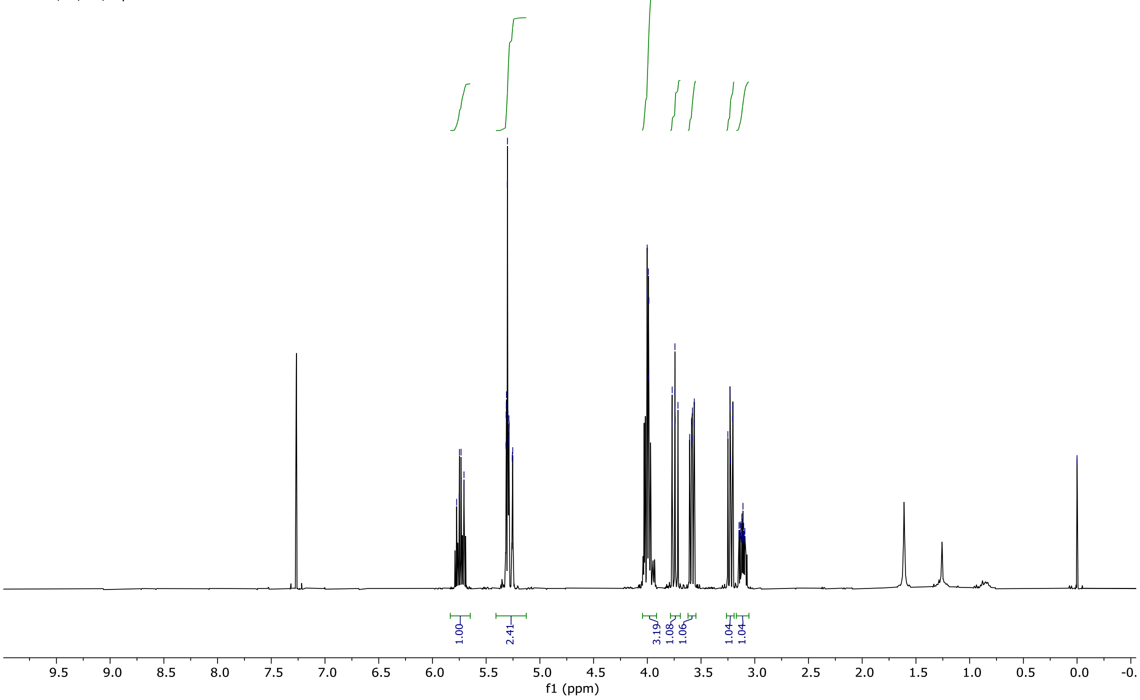

VNMRS400A_18122020_as404-2-20-C13
VNMRS400F / Num.Inv. 205984
cdcl3 / Temp: 25C / N.Reg: XXXXXXXXXX
Usuari: san / Mostra: as404-2-20
Nom: ALEXANDRA SANDOR
Data: 18/12/20 / Ope.: A.SANDOR

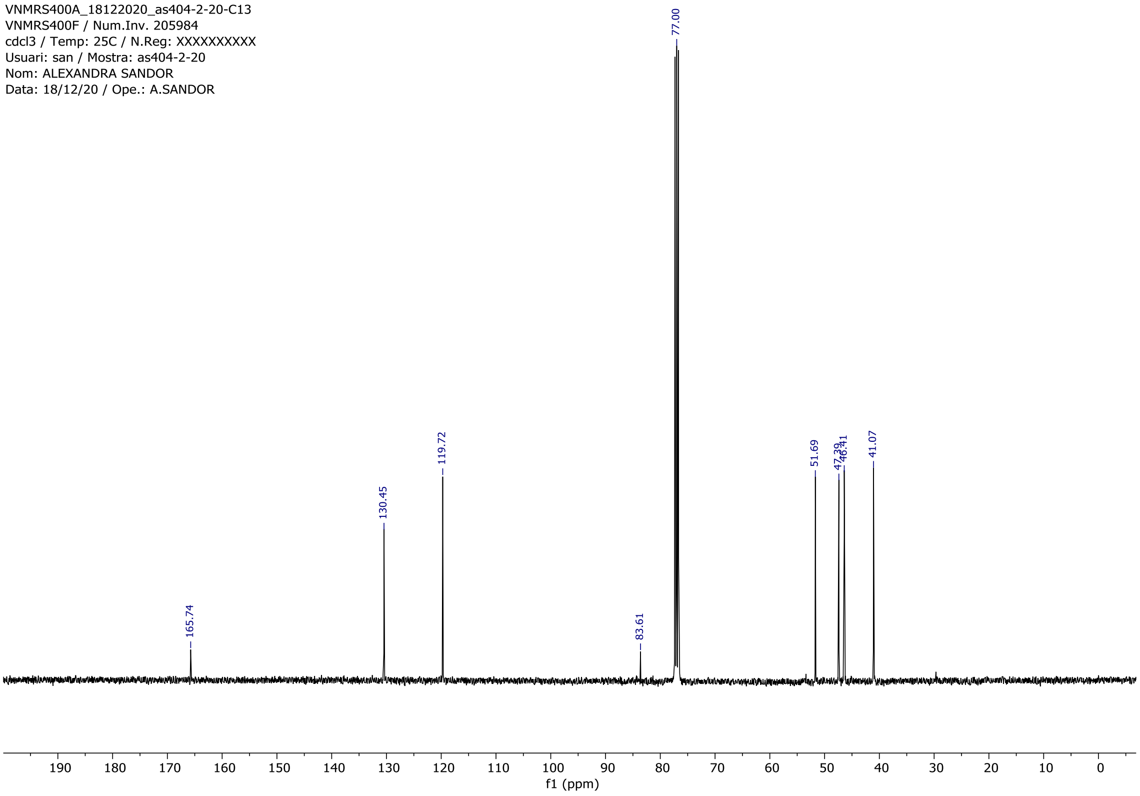

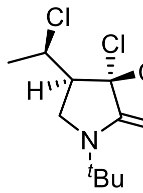

2g

VNMRS400A_07122020_as395-13-H1
VNMRS400F / Num.Inv. 205984
cdcl3 / Temp: 25C / N.Reg: XXXXXXXXXX
Usuari: san / Mostra: as395-13
Nom: ALEXANDRA SANDOR
Data: 07/12/20 / Ope.: A.SANDOR

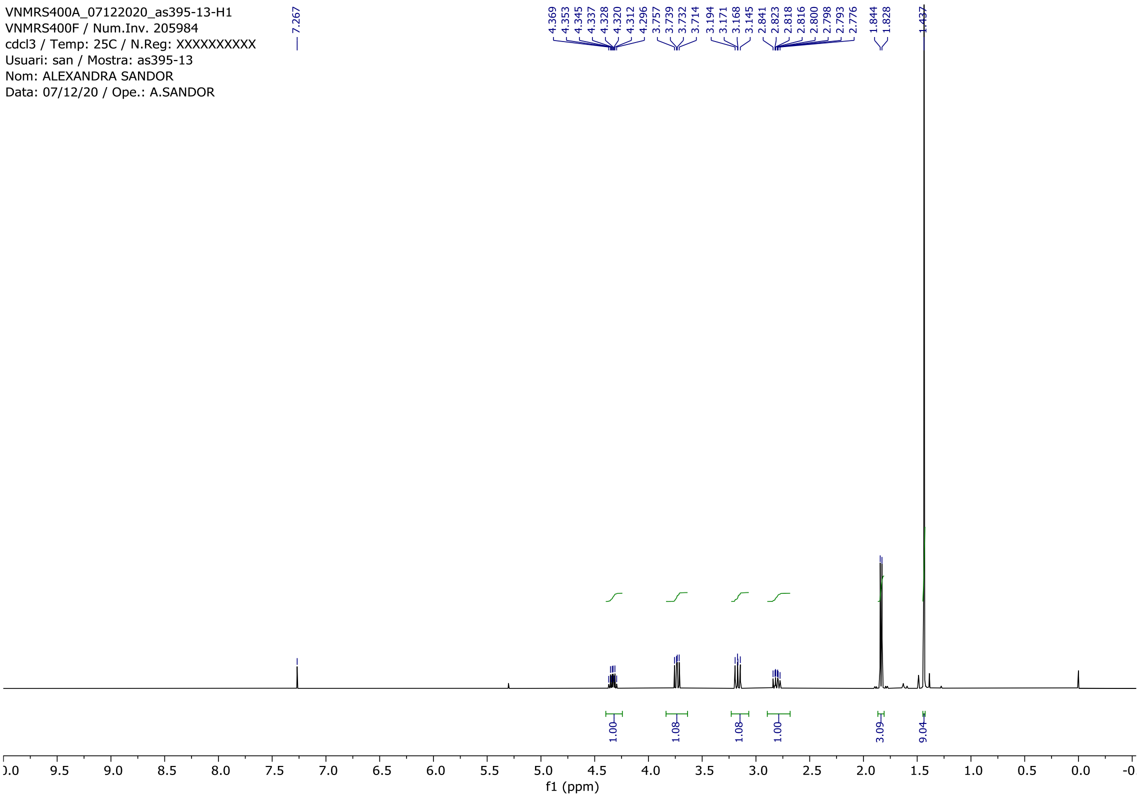

VNMRS400A_08122020_as395-13-C13
VNMRS400F / Num.Inv. 205984
cdcl3 / Temp: 25C / N.Reg: XXXXXXXXXX
Usuari: san / Mostra: as395-13
Nom: ALEXANDRA SANDOR
Data: 08/12/20 / Ope.: A.SANDOR

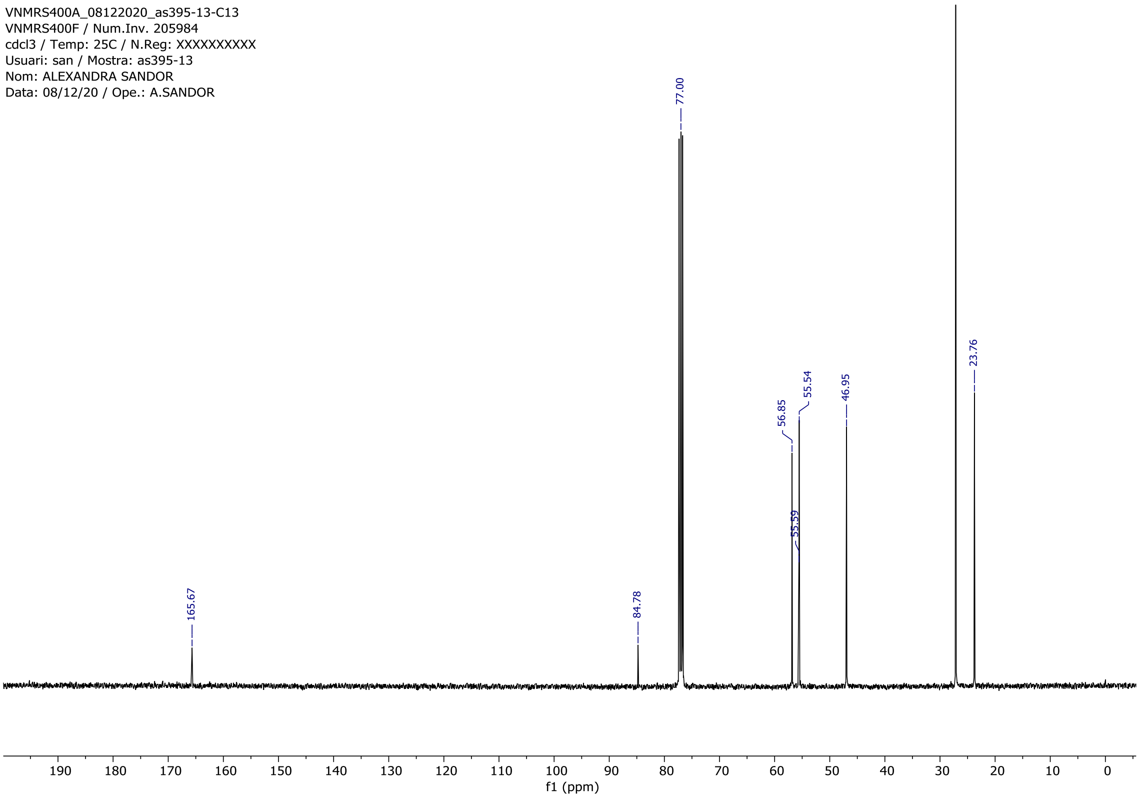

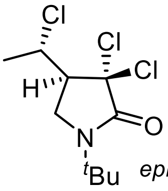

epi-**2g**

M400AFF_07122020_as395-18-H1
M400F / Num.Inv. 1009191
cdcl3 / Temp: 25C / N.Reg: XXXXXXXXXX
Usuari: san / Mostra: as395-18
Nom: ALEXANDRA SANDOR
Data: 07/12/20 / Ope.: A.SANDOR

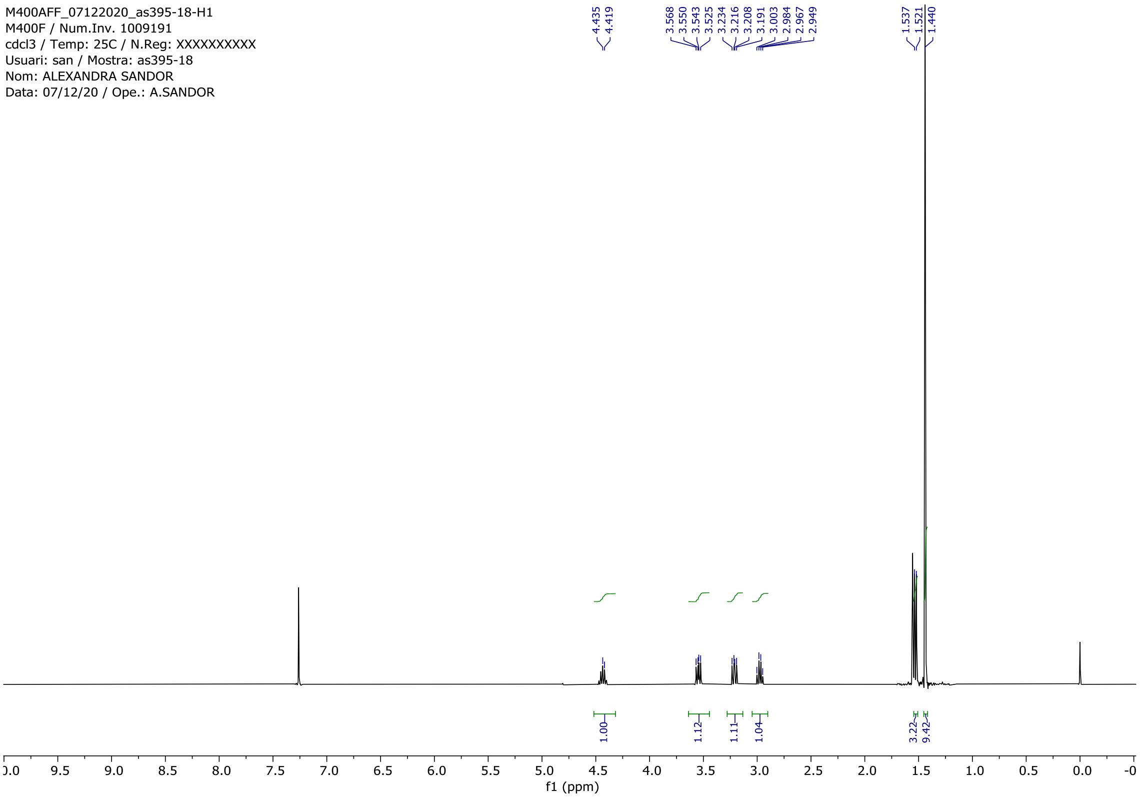

VNMRS400A_08122020_as395-18-C13
VNMRS400F / Num.Inv. 205984
cdcl3 / Temp: 25C / N.Reg: XXXXXXXXXX
Usuari: san / Mostra: as395-18
Nom: ALEXANDRA SANDOR
Data: 08/12/20 / Ope.: A.SANDOR

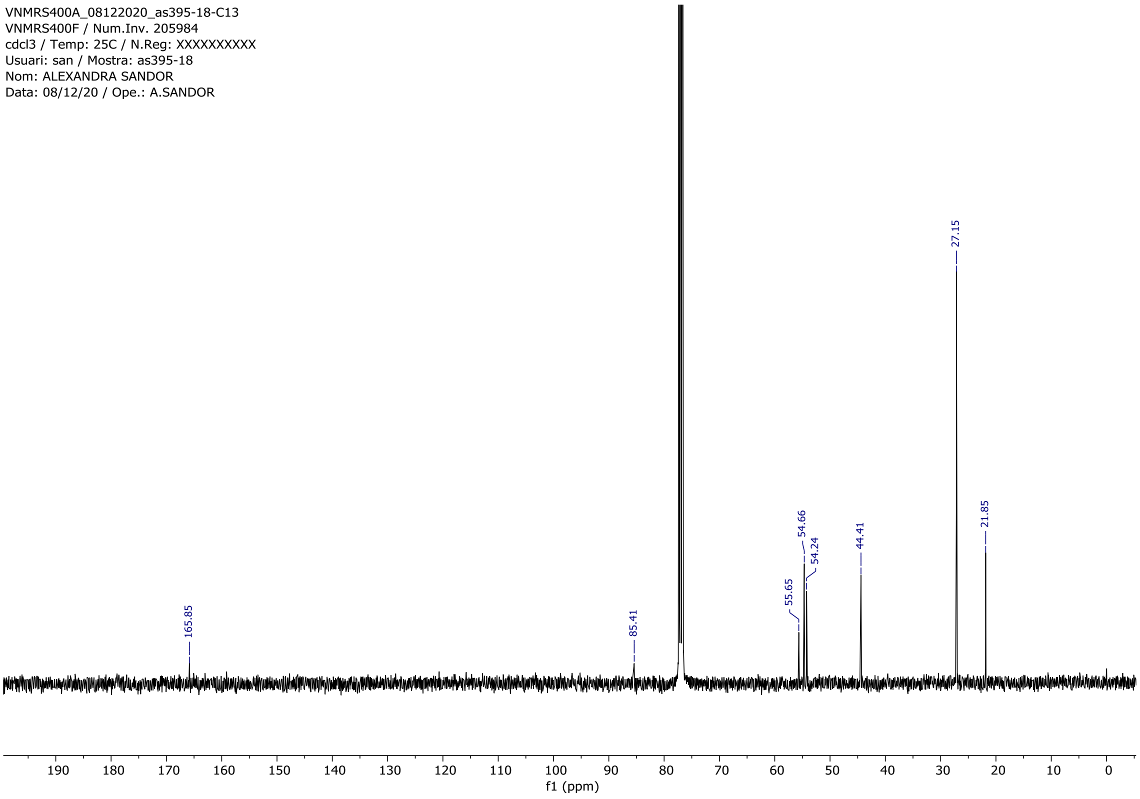

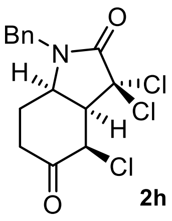

2h

M400AFF_07122020_as392-2-all-H1
M400F / Num.Inv. 1009191
cdcl3 / Temp: 25C / N.Reg: XXXXXXXXXX
Usuari: san / Mostra: as392-2-all
Nom: ALEXANDRA SANDOR
Data: 07/12/20 / Ope.: A.SANDOR

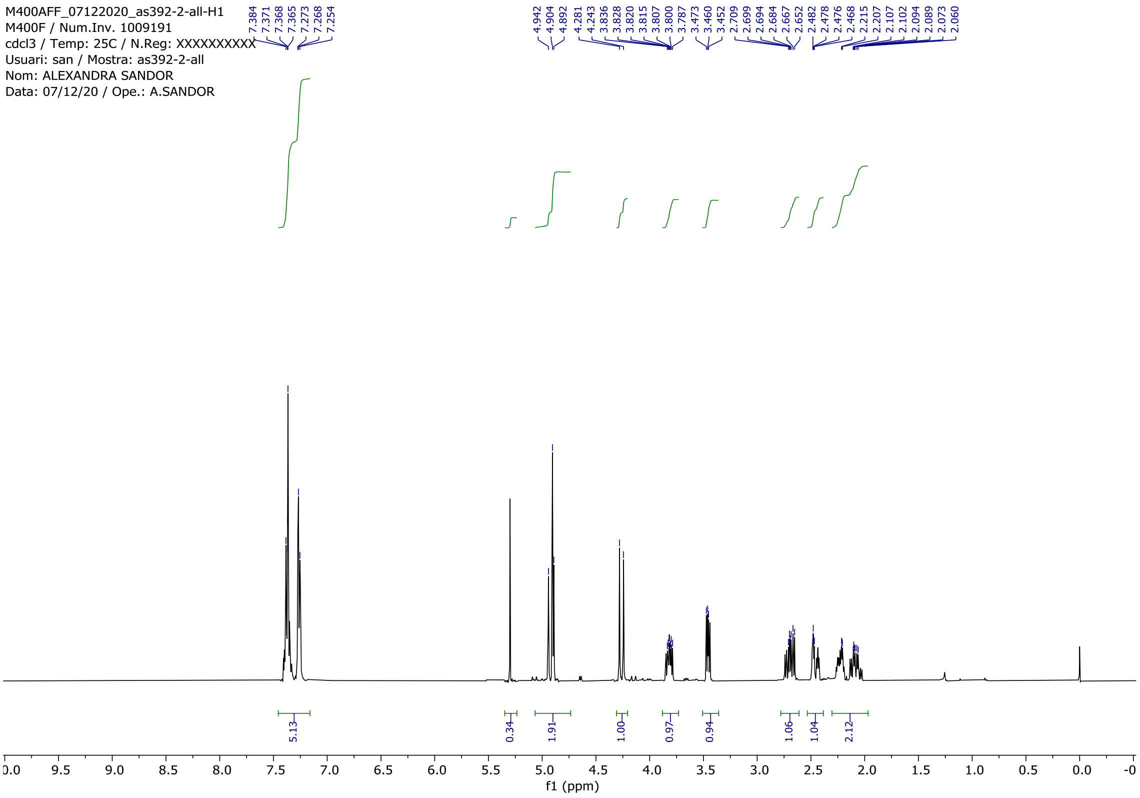

VNMRS400A_04122020_as392-2-all-C13
VNMRS400F / Num.Inv. 205984
cdcl3 / Temp: 25C / N.Reg: XXXXXXXXXX
Usuari: san / Mostra: as392-2-all
Nom: ALEXANDRA SANDOR
Data: 04/12/20 / Ope.: A.SANDOR

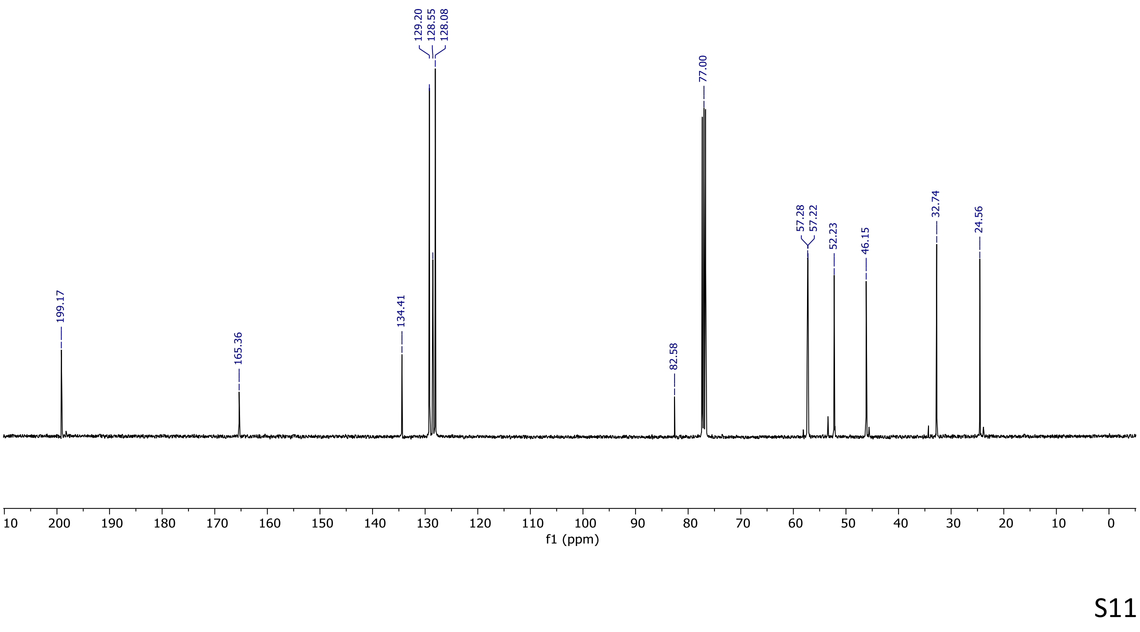

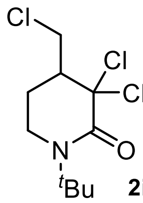

2i

M400AFF_13122020_AS400-7-H1_rep_20_18_01
M400F / Num.Inv. 1009191
cdcl3 / Temp: 25C / N.Reg: XXXXXXXXXX
Usuari: san / Mostra: AS400-7
Nom: ALEXANDRA SANDOR
Data: 13/12/20 / Ope.: A.SANDOR

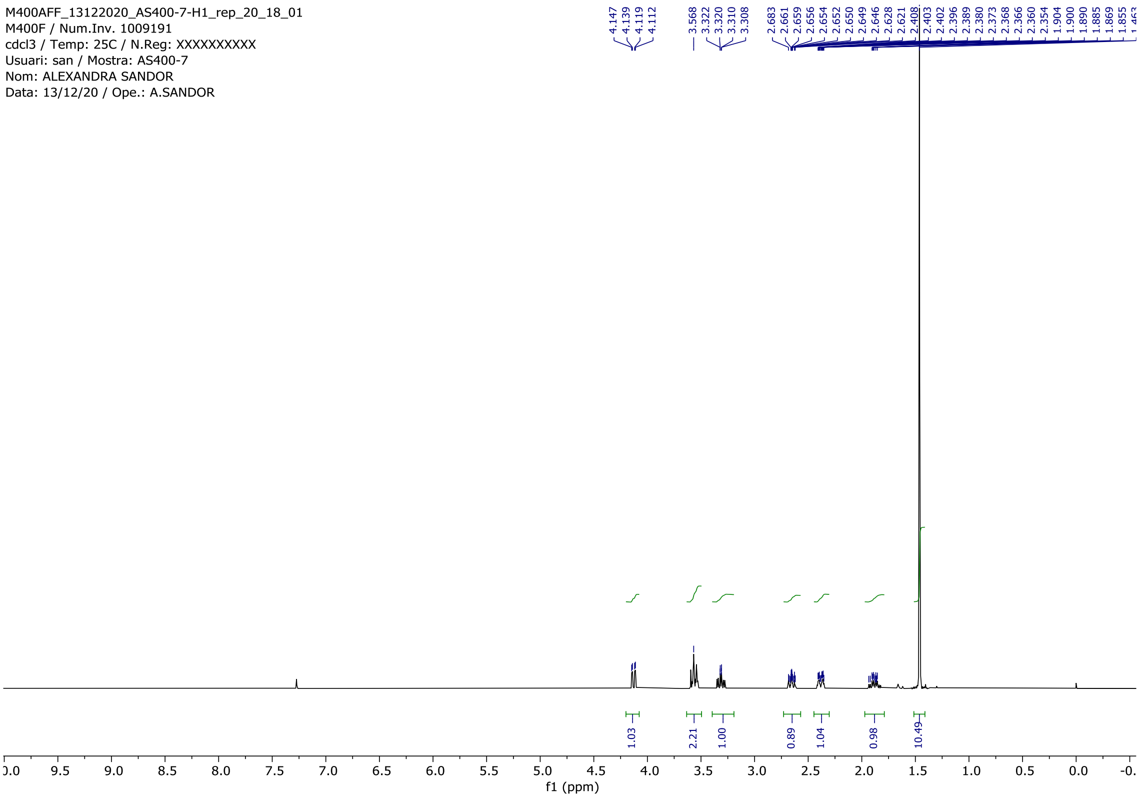

VNMRS400A_14122020_as400-7-C13
VNMRS400F / Num.Inv. 205984
cdcl3 / Temp: 25C / N.Reg: XXXXXXXXXX
Usuari: san / Mostra: as400-7
Nom: ALEXANDRA SANDOR
Data: 14/12/20 / Ope.: A.SANDOR

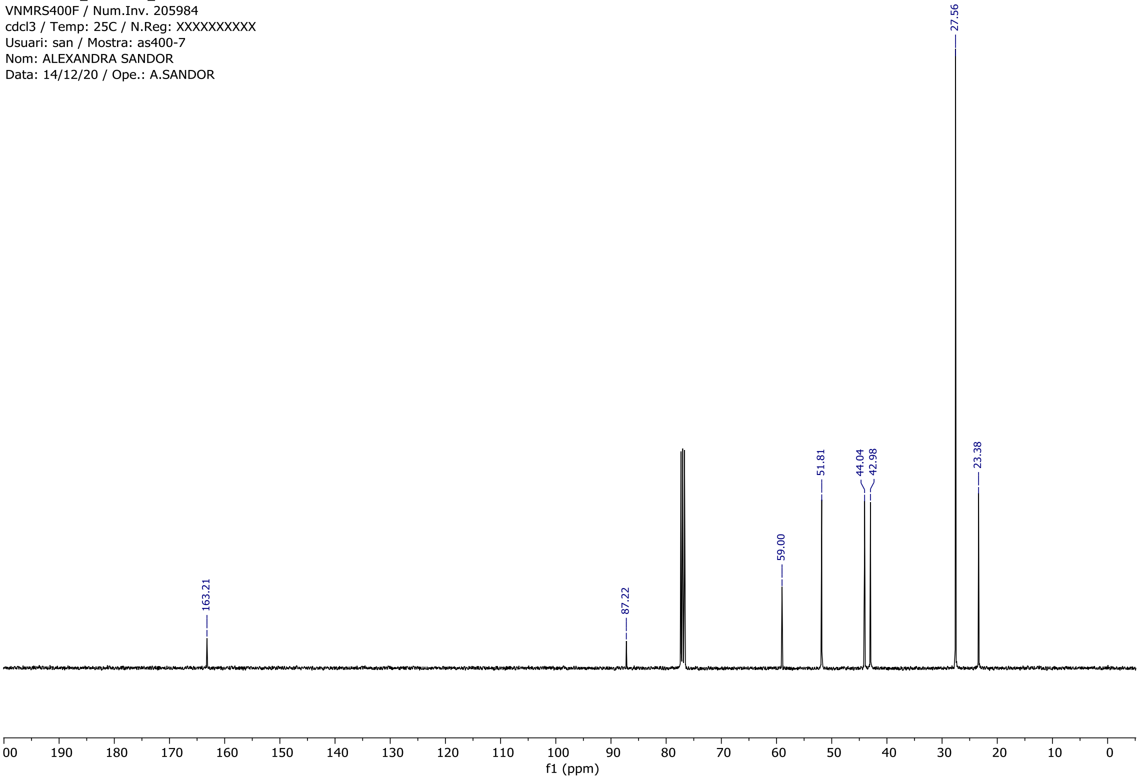

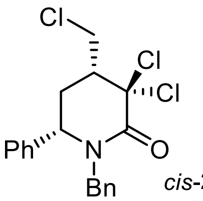

cis-**2j**

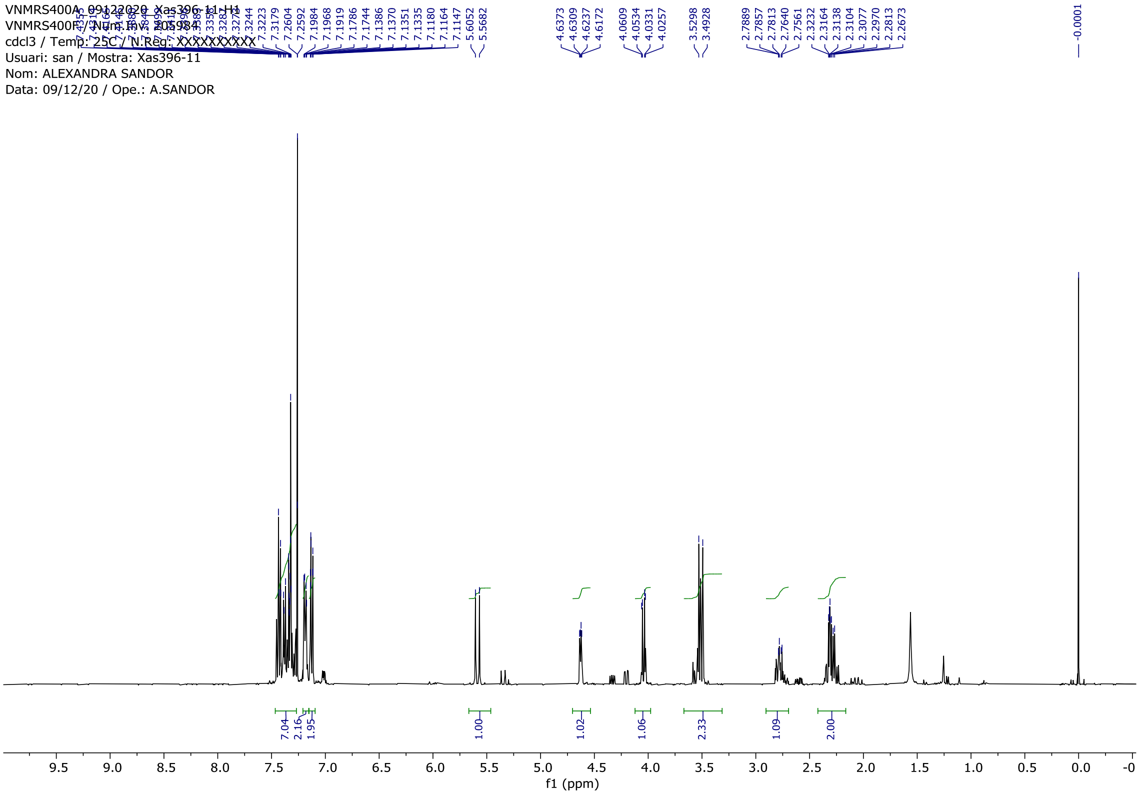
VNMRS400A_09122020_Xas396-11-H1
VNMRS400F / Num.Inv. 205984
cdcl3 / Temp: 25C / N.Reg: XXXXXXXXXX
Usuari: san / Mostra: Xas396-11
Nom: ALEXANDRA SANDOR
Data: 09/12/20 / Ope.: A.SANDOR
f1 (ppm)

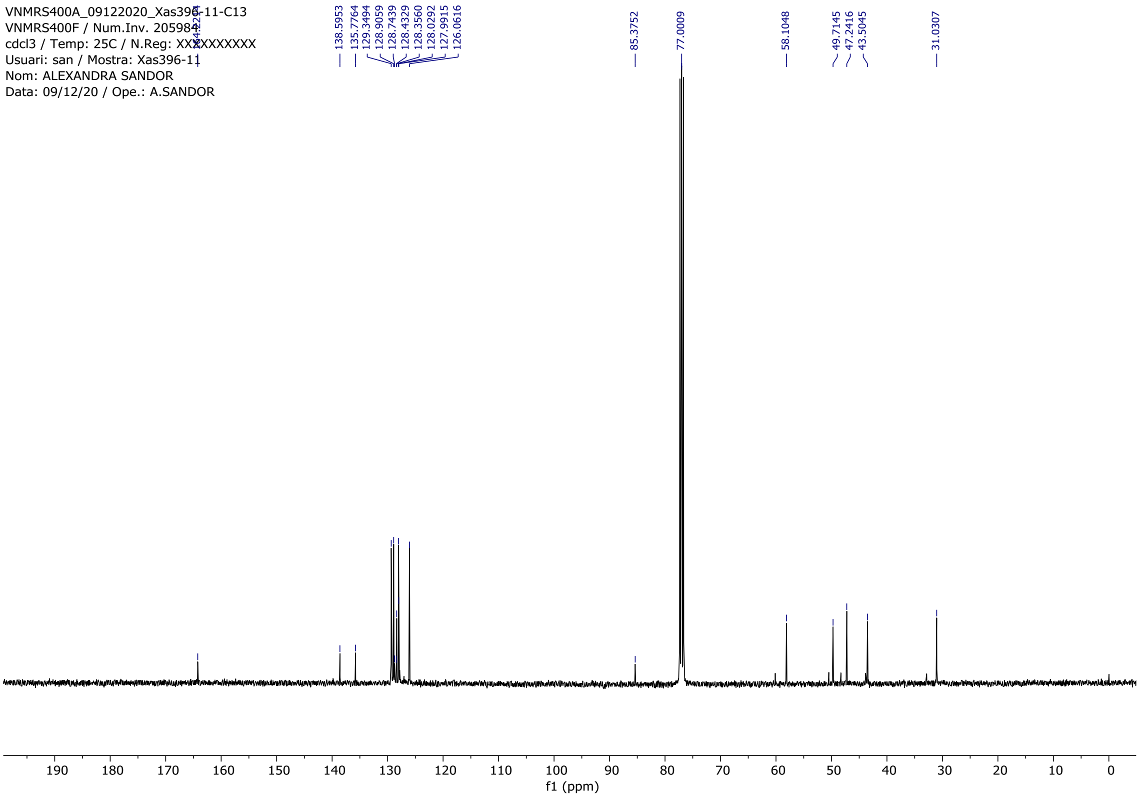
VNMRS400A_09122020_Xas396-11-C13
VNMRS400F / Num.Inv. 205984
cdcl3 / Temp: 25C / N.Reg: XXXXXXXXXX
Usuari: san / Mostra: Xas396-11
Nom: ALEXANDRA SANDOR
Data: 09/12/20 / Ope.: A.SANDOR
138.5953
135.7764
129.3494
128.9059
128.7439
128.4329
128.3560
128.0292
127.9915
126.0616
85.3752
77.0009
58.1048
49.7145
47.2416
43.5045
31.0307
f1 (ppm)

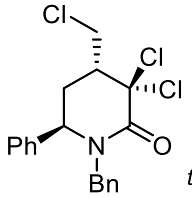
Cl
Cl
Cl
Ph
N
O
Bn

trans-**2j**

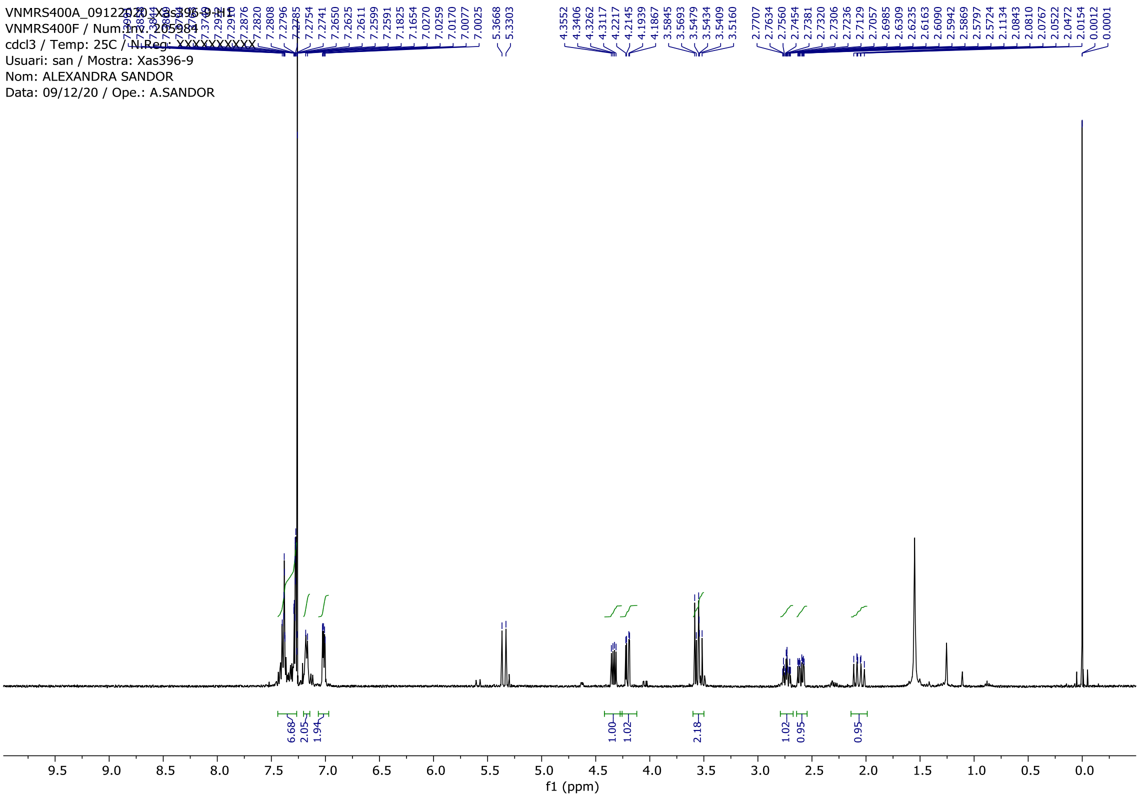
VNMRS400A_09122020_Xas396-9-H1
VNMRS400F / Num.Inv. 205984
cdcl3 / Temp: 25C / N.Reg: XXXXXXXXXX
Usuari: san / Mostra: Xas396-9
Nom: ALEXANDRA SANDOR
Data: 09/12/20 / Ope.: A.SANDOR
7.2876
7.2820
7.2808
7.2796
7.2785
7.2754
7.2741
7.2650
7.2625
7.2611
7.2599
7.2591
7.1825
7.1654
7.0270
7.0259
7.0170
7.0077
7.0025
5.3668
5.3303
4.3552
4.3406
4.3262
4.3117
4.2217
4.2145
4.1939
4.1867
3.5845
3.5693
3.5479
3.5434
3.5409
3.5160
2.7707
2.7634
2.7560
2.7454
2.7381
2.7320
2.7306
2.7236
2.7129
2.7057
2.6985
2.6309
2.6235
2.6163
2.6090
2.5942
2.5869
2.5797
2.5724
2.1134
2.0843
2.0810
2.0767
2.0522
2.0472
2.0154
0.0012
0.0001
6.68
2.05
1.94
1.00
1.02
2.18
1.02
0.95
0.95
9.5
9.0
8.5
8.0
7.5
7.0
6.5
6.0
5.5
5.0
4.5
4.0
3.5
3.0
2.5
2.0
1.5
1.0
0.5
0.0
f1 (ppm)

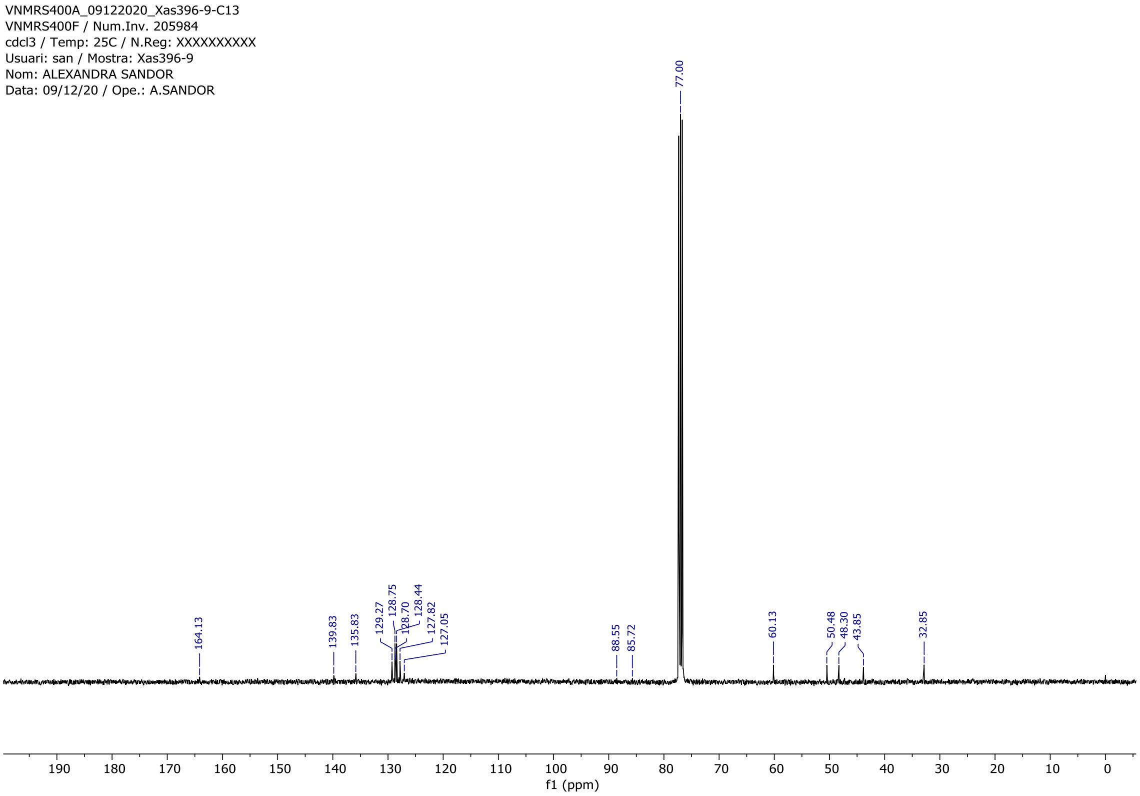
VNMRS400A_09122020_Xas396-9-C13
VNMRS400F / Num.Inv. 205984
cdcl3 / Temp: 25C / N.Reg: XXXXXXXXXX
Usuari: san / Mostra: Xas396-9
Nom: ALEXANDRA SANDOR
Data: 09/12/20 / Ope.: A.SANDOR
77.00
164.13
139.83
135.83
129.27
128.75
128.70
128.44
127.82
127.05
88.55
85.72
60.13
50.48
48.30
43.85
32.85
190
180
170
160
150
140
130
120
110
100
90
80
70
60
50
40
30
20
10
0
f1 (ppm)

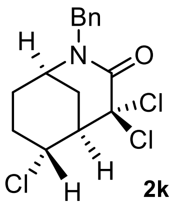

2k

vnmrs400_0503-R47-9CRYSTALS-H1
H1 / Mercury-400F
cdcl3 / Temp: Ambient / N.Reg: XXXXXXXXXX
Usuari: san / Mostra: R47-9CRYSTALS
Nom: FAIZA DIABA
Data: 03/05/11 / Ope.: F.DIABA
Experiment: s2pul

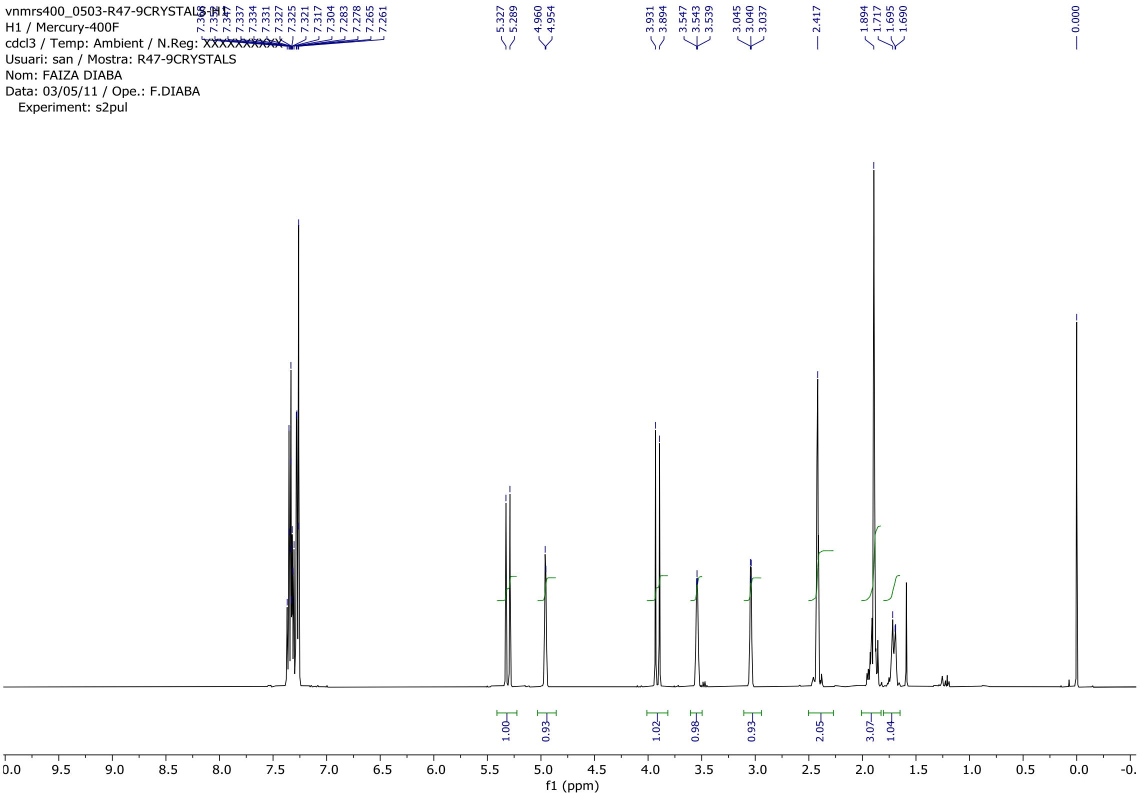

vnmrs400_0503-R47-9CRYSTALS-C13
H1 / Mercury-400F
cdcl3 / Temp: Ambient / N.Reg: XXXXXXXXXX
Usuari: san / Mostra: R47-9CRYSTALS
Nom: FAIZA DIABA
Data: 03/05/11 / Ope.: F.DIABA
Experiment: s2pul

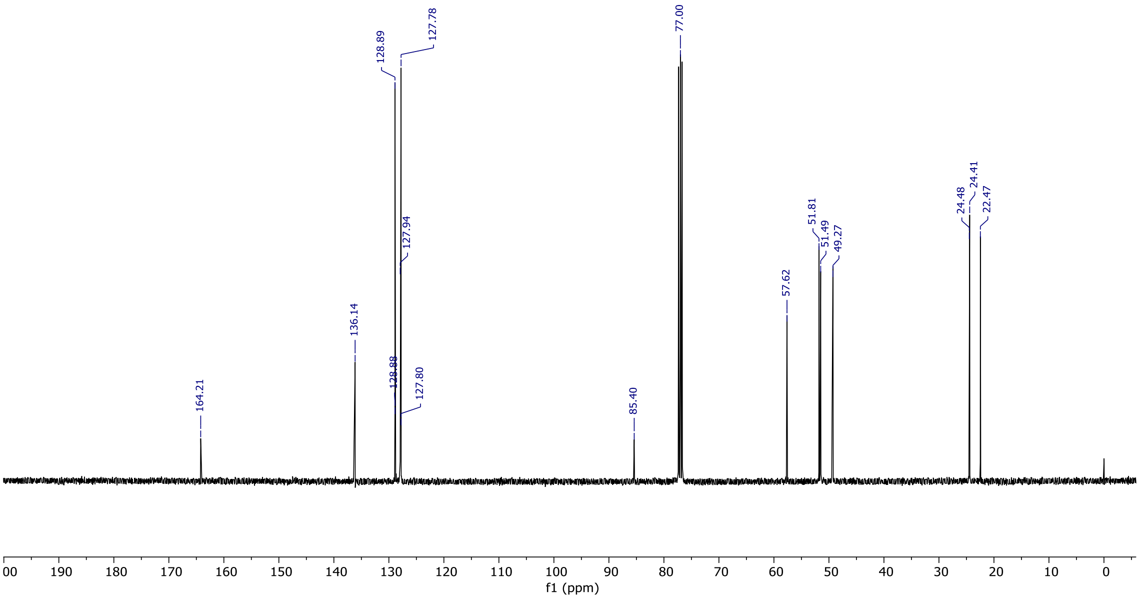

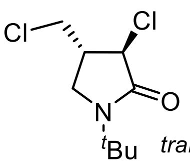

trans-**2l** (major isomer)

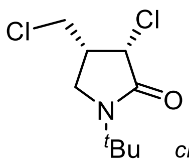

cis-**2l** (minor isomer)

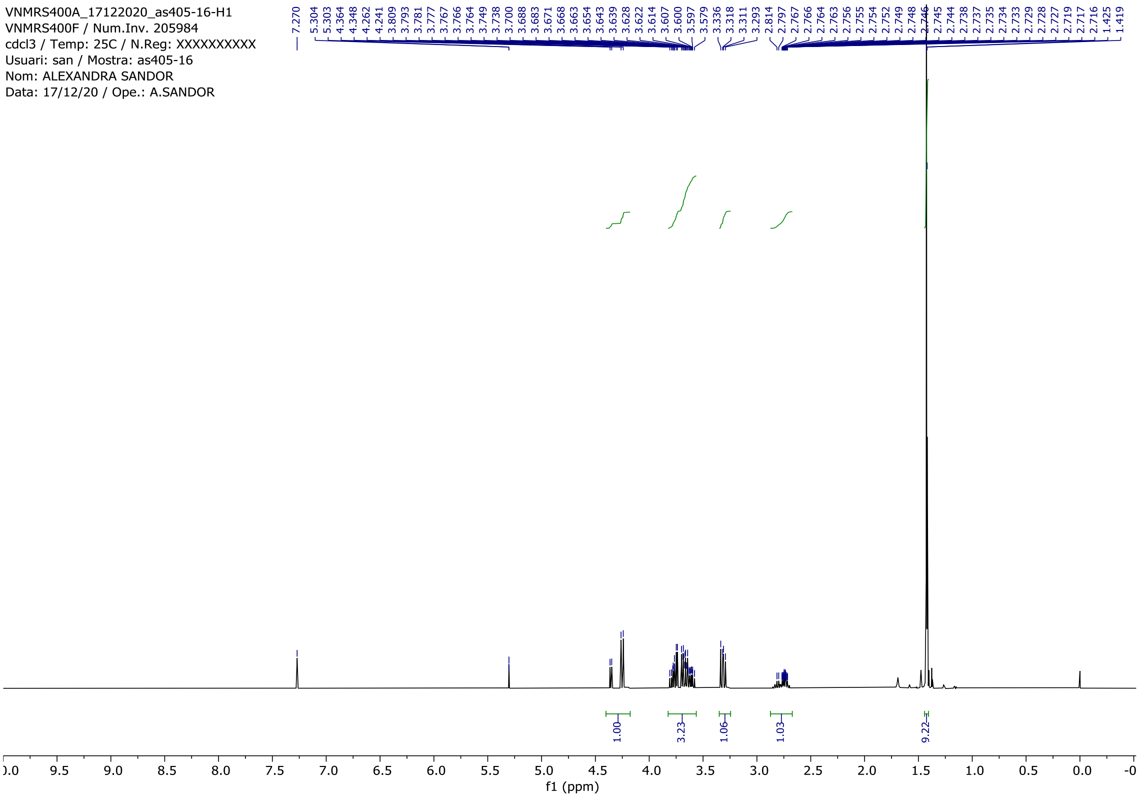

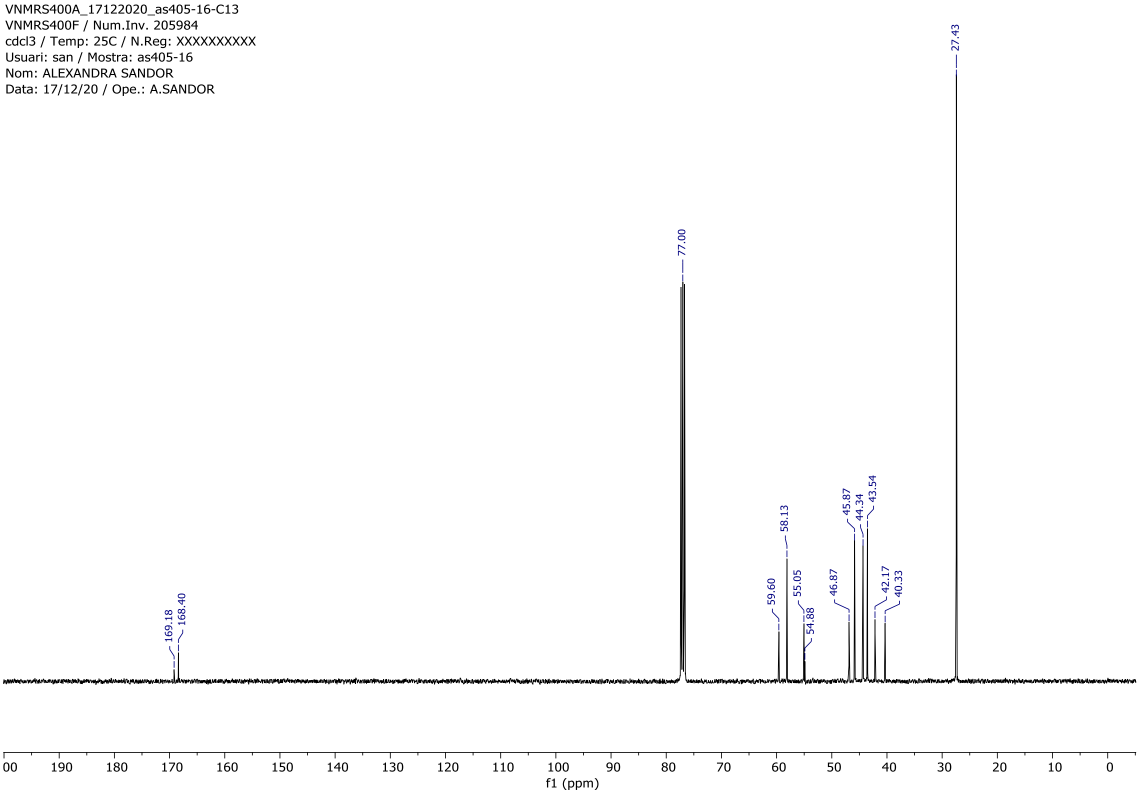

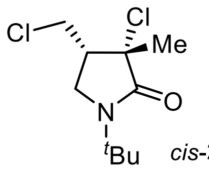

cis-**2m** (major isomer)

VNMRS400A_25012021_yAS411-8-H1_rep_15_56_10
VNMRS400F / Num.Inv. 205984
cdcl3 / Temp: 25C / N.Reg: XXXXXXXXXX
Usuari: san / Mostra: yAS411-8
Nom: FAIZA DIABA
Data: 25/01/21 / Ope.: F.DIABA

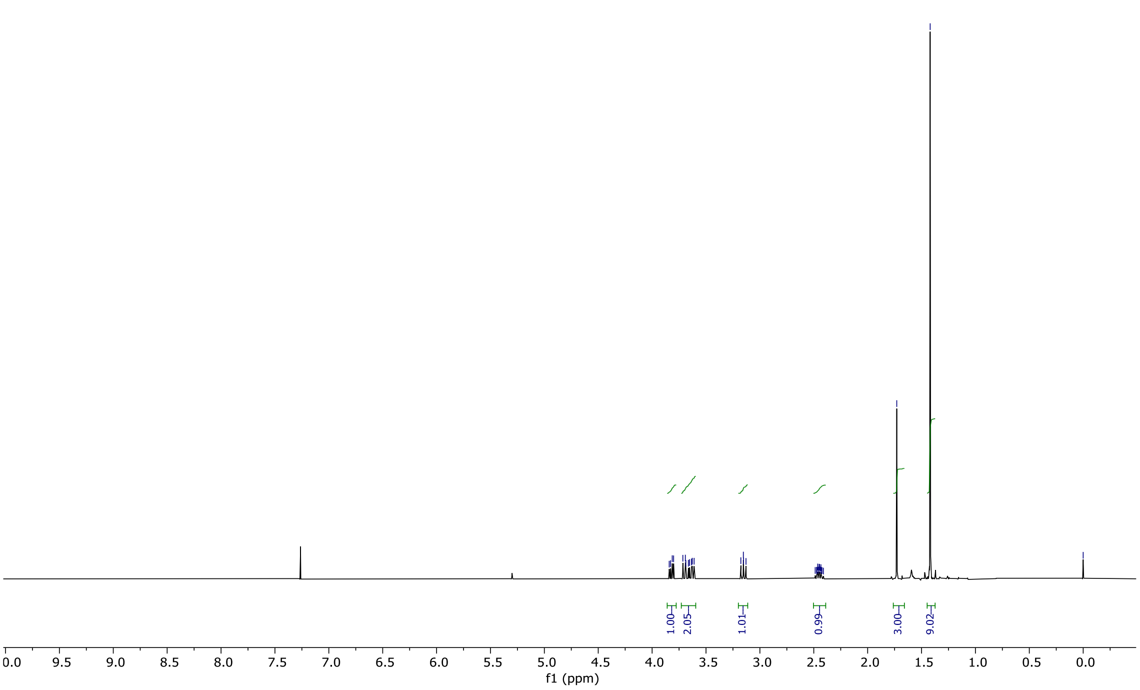

VNMRS400A_25012021_XAS411-8-C13
VNMRS400F / Num.Inv. 205984
cdcl3 / Temp: 25C / N.Reg: XXXXXXXXXX
Usuari: san / Mostra: XAS411-8
Nom: FAIZA DIABA
Data: 25/01/21 / Ope.: F.DIABA

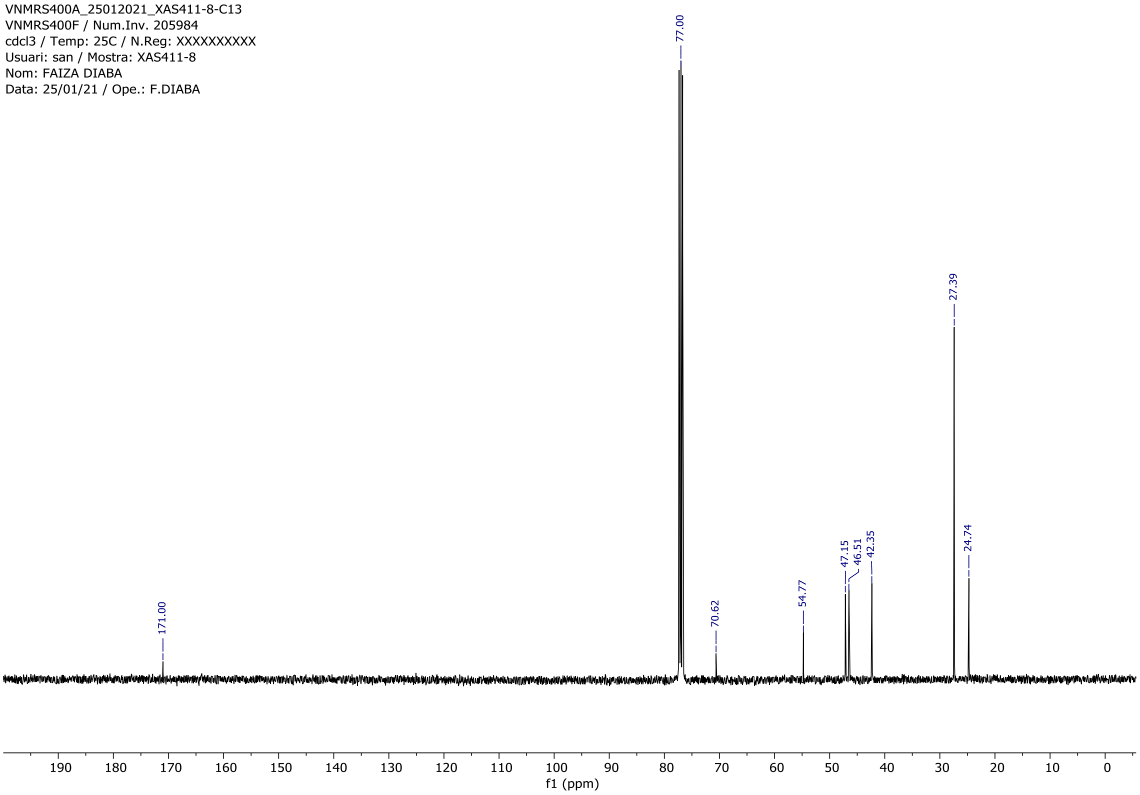

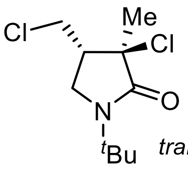

trans-**2m** (minor isomer)

VNMRS400A_26012021_AS411-2-19-H1_rep_22_54_43
VNMRS400F / Num.Inv. 205984
cdcl3 / Temp: 25C / N.Reg: XXXXXXXXXX
Usuari: san / Mostra: AS411-2-19
Nom: ALEXANDRA SANDOR
Data: 26/01/21 / Ope.: A.SANDOR

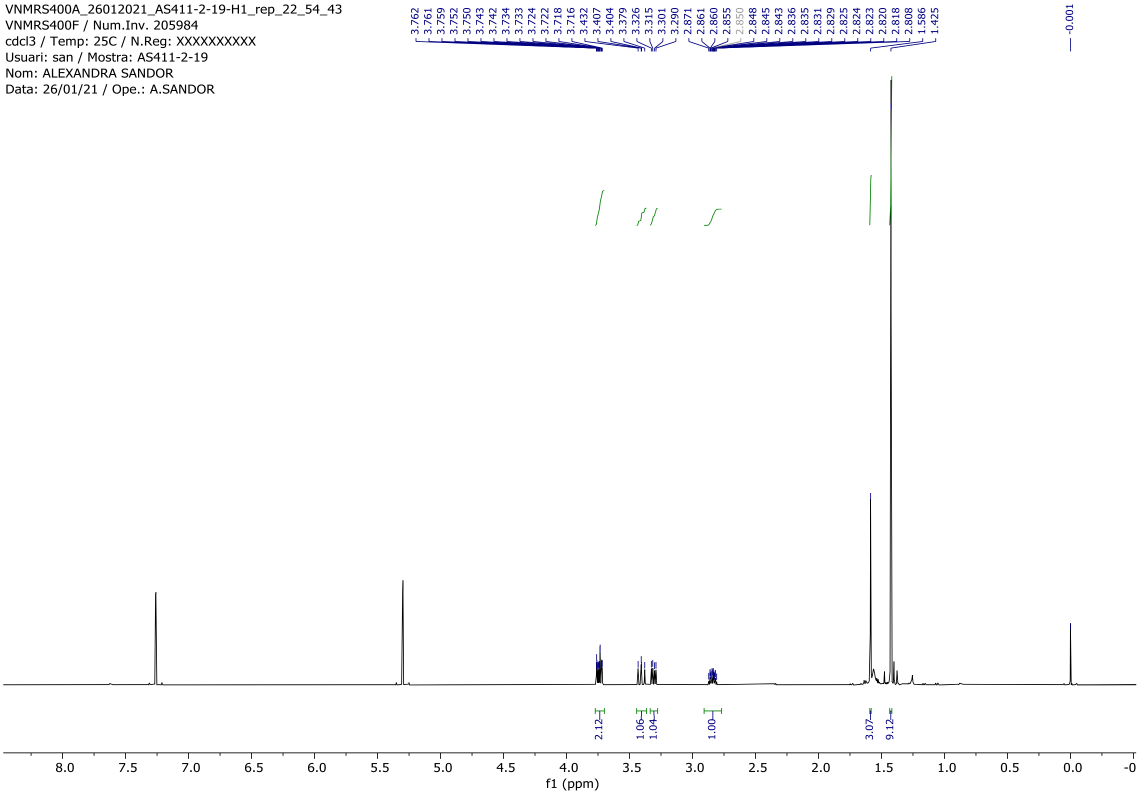

VNMRS400A_26012021_AS411-2-19-C13
VNMRS400F / Num.Inv. 205984
cdcl3 / Temp: 25C / N.Reg: XXXXXXXXXX
Usuari: san / Mostra: AS411-2-19
Nom: ALEXANDRA SANDOR
Data: 26/01/21 / Ope.: A.SANDOR

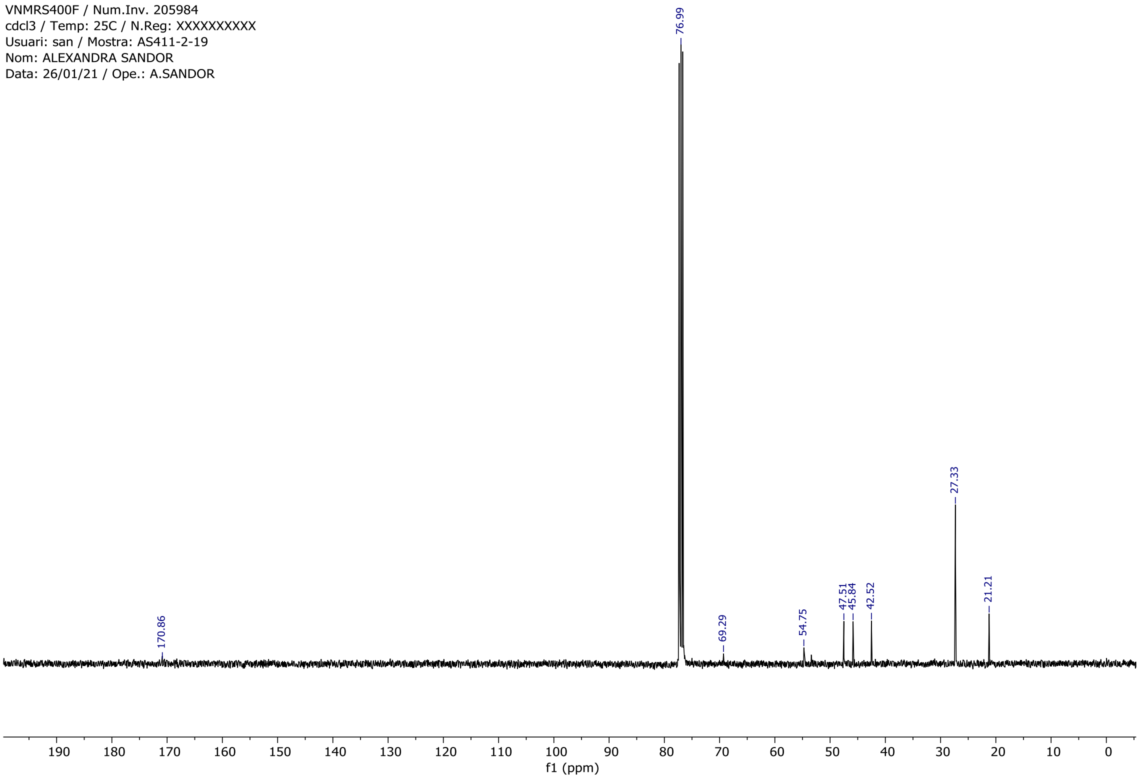

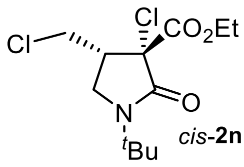

cis-**2n** (less polar)

M400AFF_15122020_AS401-2-8-H1
M400F / Num.Inv. 1009191
cdcl3 / Temp: 25C / N.Reg: XXXXXXXXXX
Usuari: san / Mostra: AS401-2-8
Nom: ALEXANDRA SANDOR
Data: 15/12/20 / Ope.: A.SANDOR

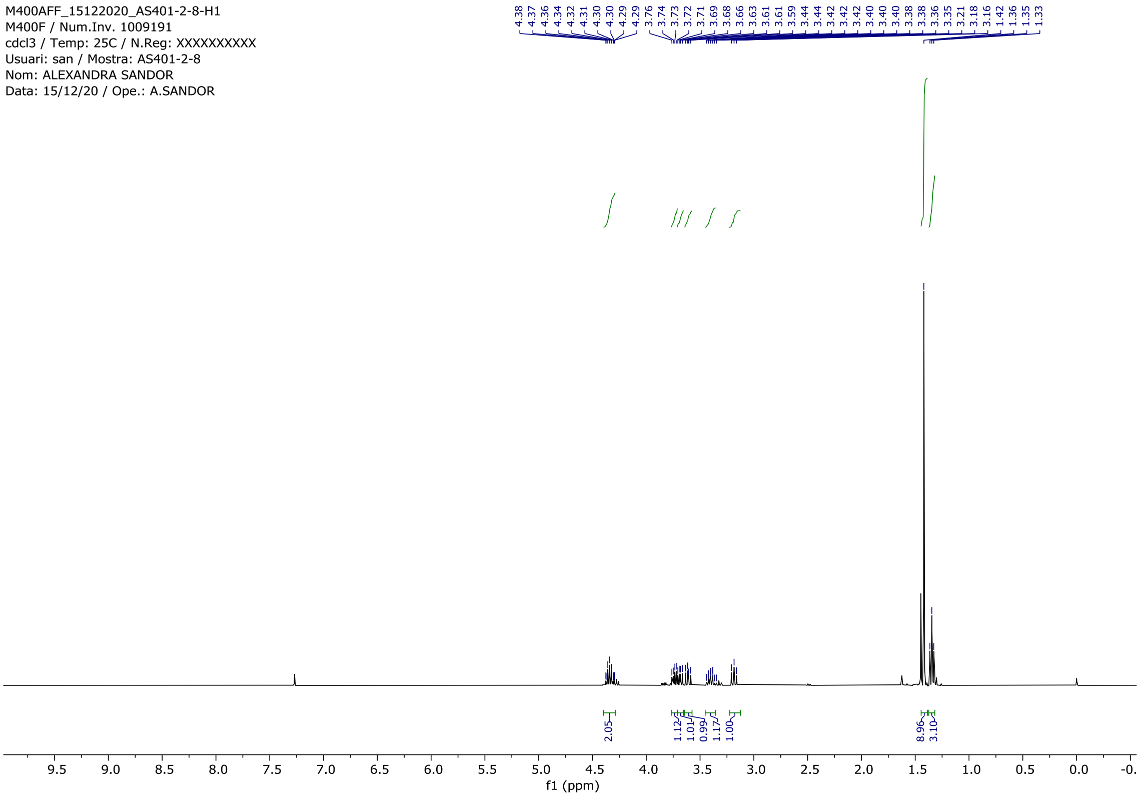

VNMRS400A_17122020_as401-2-8-C13
VNMRS400F / Num.Inv. 205984
cdcl3 / Temp: 25C / N.Reg: XXXXXXXXXX
Usuari: san / Mostra: as401-2-8
Nom: ALEXANDRA SANDOR
Data: 17/12/20 / Ope.: A.SANDOR

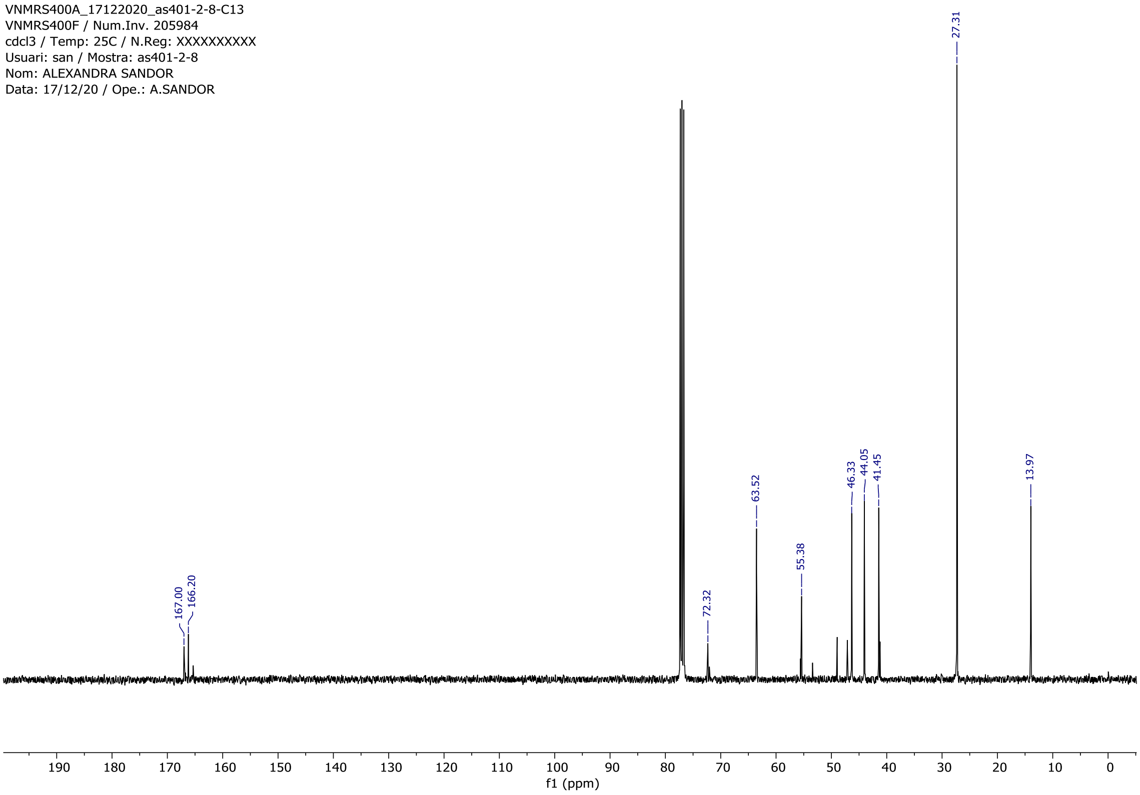

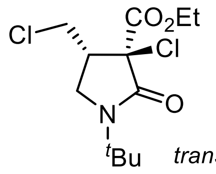

trans-**2n** (more polar)

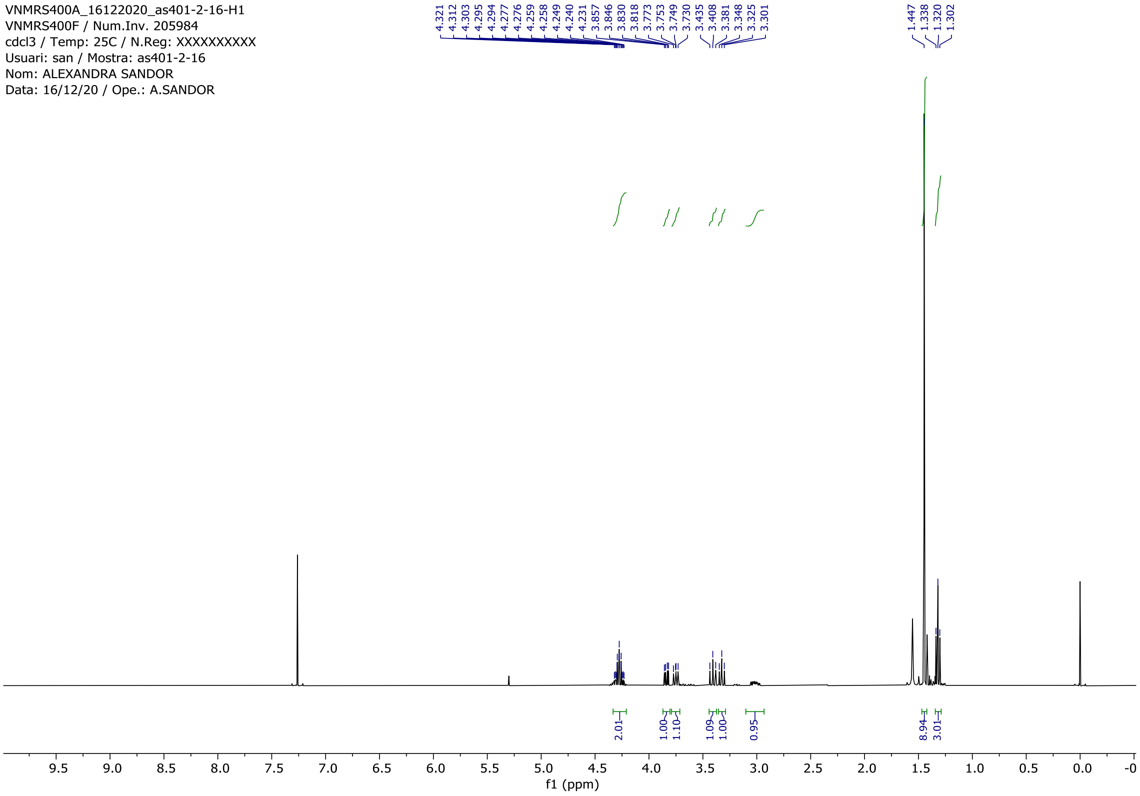

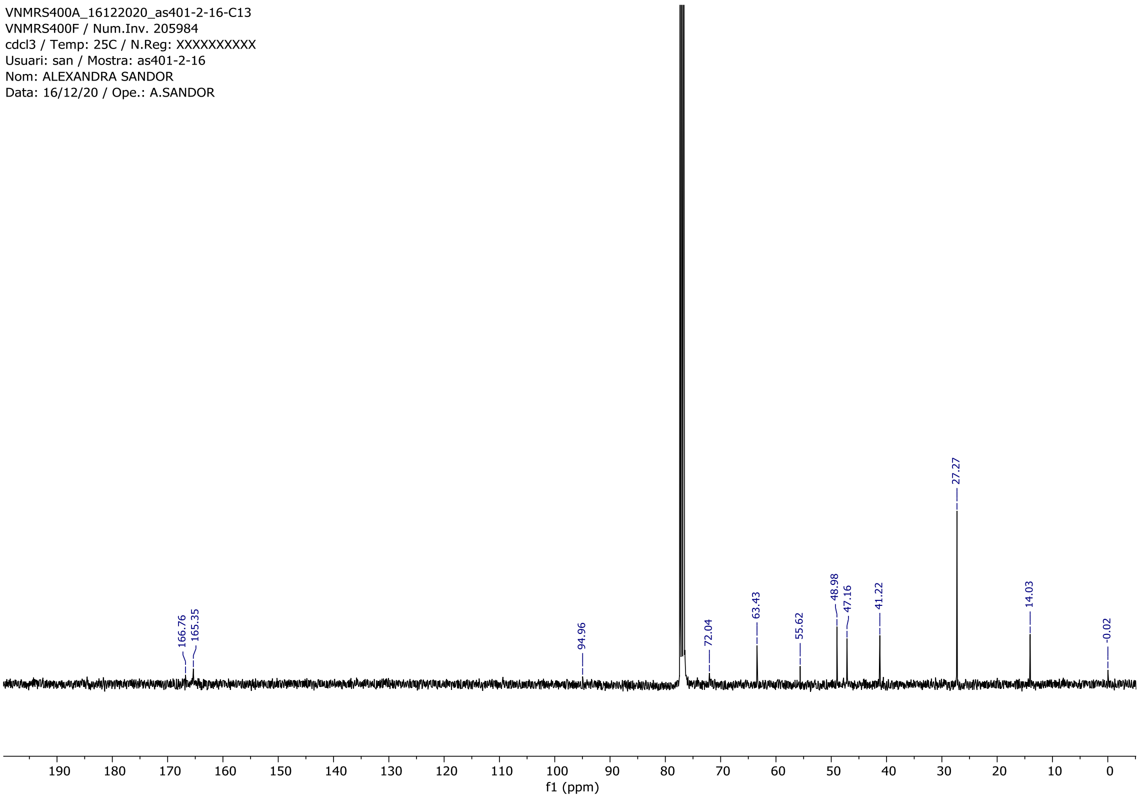

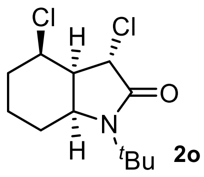
Cl
H
Cl
O
N
H
tBu

2o

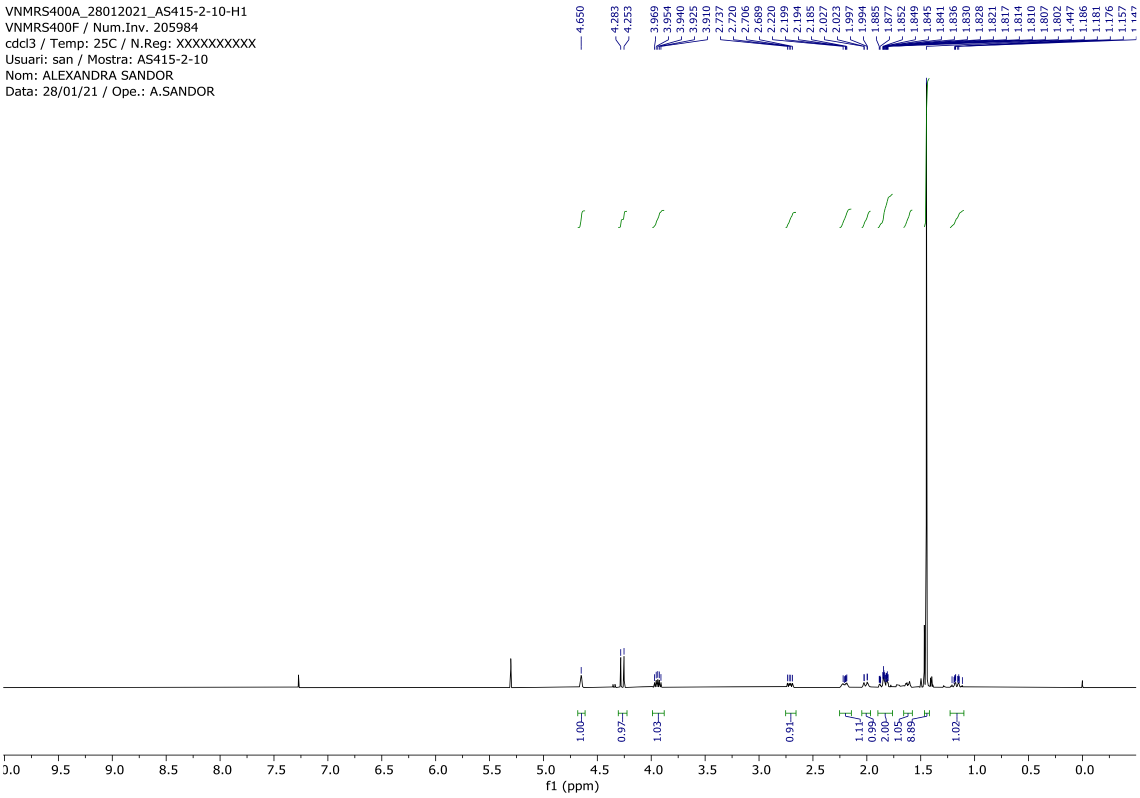
VNMRS400A_28012021_AS415-2-10-H1
VNMRS400F / Num.Inv. 205984
cdcl3 / Temp: 25C / N.Reg: XXXXXXXXXX
Usuari: san / Mostra: AS415-2-10
Nom: ALEXANDRA SANDOR
Data: 28/01/21 / Ope.: A.SANDOR
4.650
4.283
4.253
3.969
3.954
3.940
3.925
3.910
2.737
2.720
2.706
2.689
2.220
2.199
2.194
2.185
2.027
2.023
1.997
1.994
1.885
1.877
1.852
1.849
1.845
1.841
1.836
1.830
1.828
1.821
1.817
1.814
1.810
1.807
1.802
1.447
1.186
1.181
1.176
1.157
1.00
0.97
1.03
0.91
1.11
0.99
2.00
1.05
8.89
1.02
f1 (ppm)

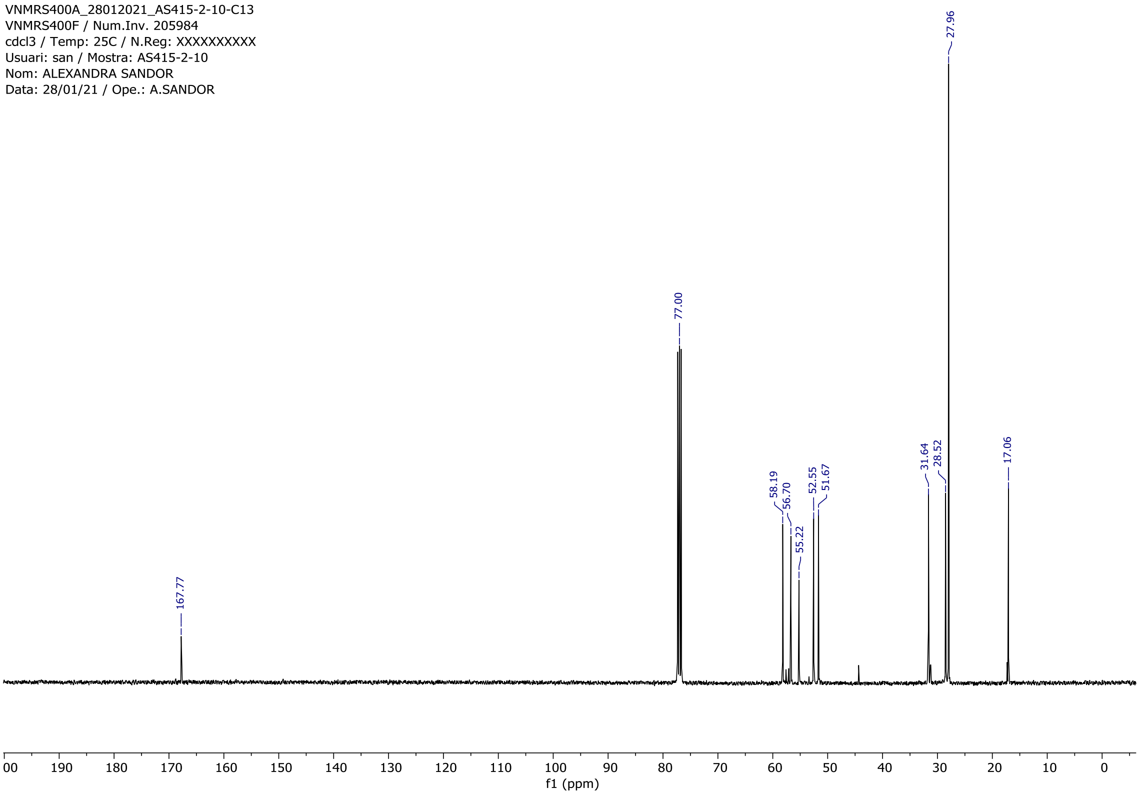
VNMRS400A_28012021_AS415-2-10-C13
VNMRS400F / Num.Inv. 205984
cdcl3 / Temp: 25C / N.Reg: XXXXXXXXXX
Usuari: san / Mostra: AS415-2-10
Nom: ALEXANDRA SANDOR
Data: 28/01/21 / Ope.: A.SANDOR
27.96
77.00
167.77
58.19
56.70
55.22
52.55
51.67
31.64
28.52
17.06
f1 (ppm)

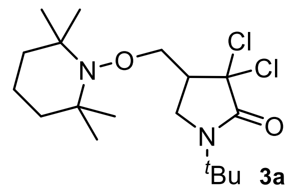

3a

M400AQUI_17062021_XGT323-12-18-H1
M400Q / Num.Inv. AF/004285
cdcl3 / Temp: 25C / N.Reg: XXXXXXXXXX
Usuari: san / Mostra: XGT323-12-18
Nom: GISELA TRENCHS MIR
Data: 17/06/21 / Ope.: G.TRENCHS

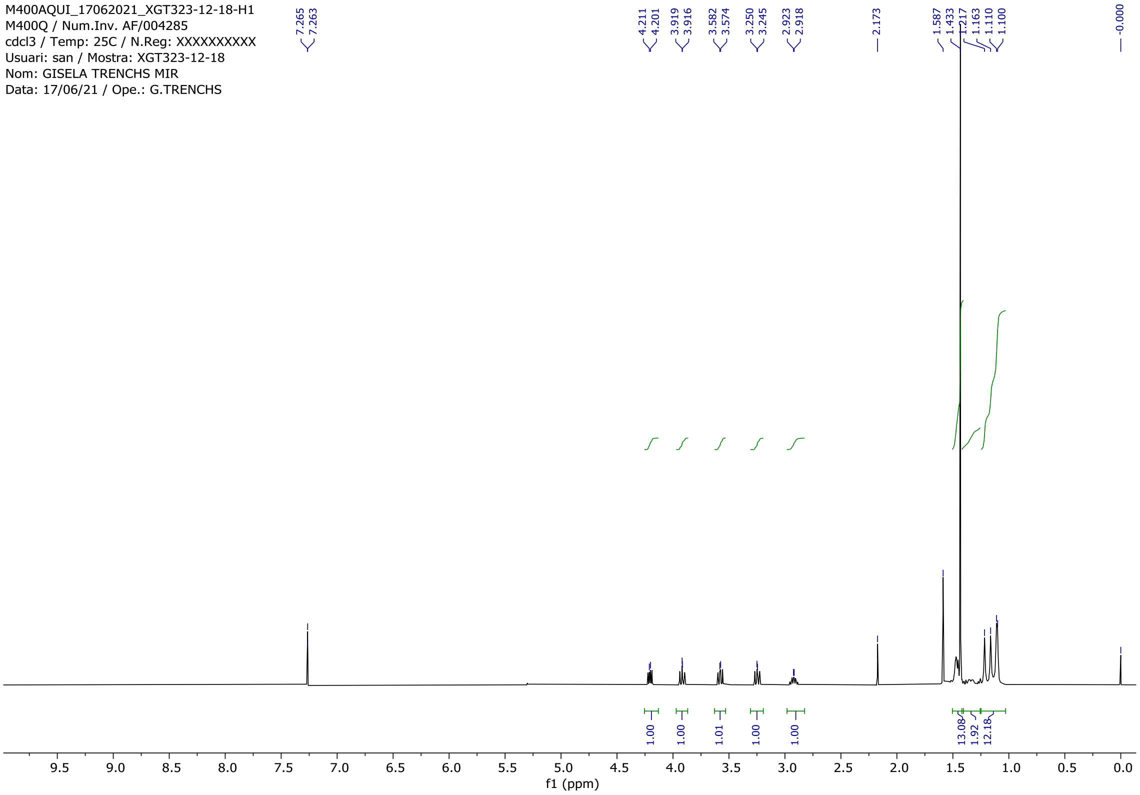

M400AQUI_17062021_XGT323-12-18-C13
M400Q / Num.Inv. AF/004285
cdcl3 / Temp: 25C / N.Reg: XXXXXXXXXX
Usuari: san / Mostra: XGT323-12-18
Nom: GISELA TRENCHS MIR
Data: 17/06/21 / Ope.: G.TRENCHS

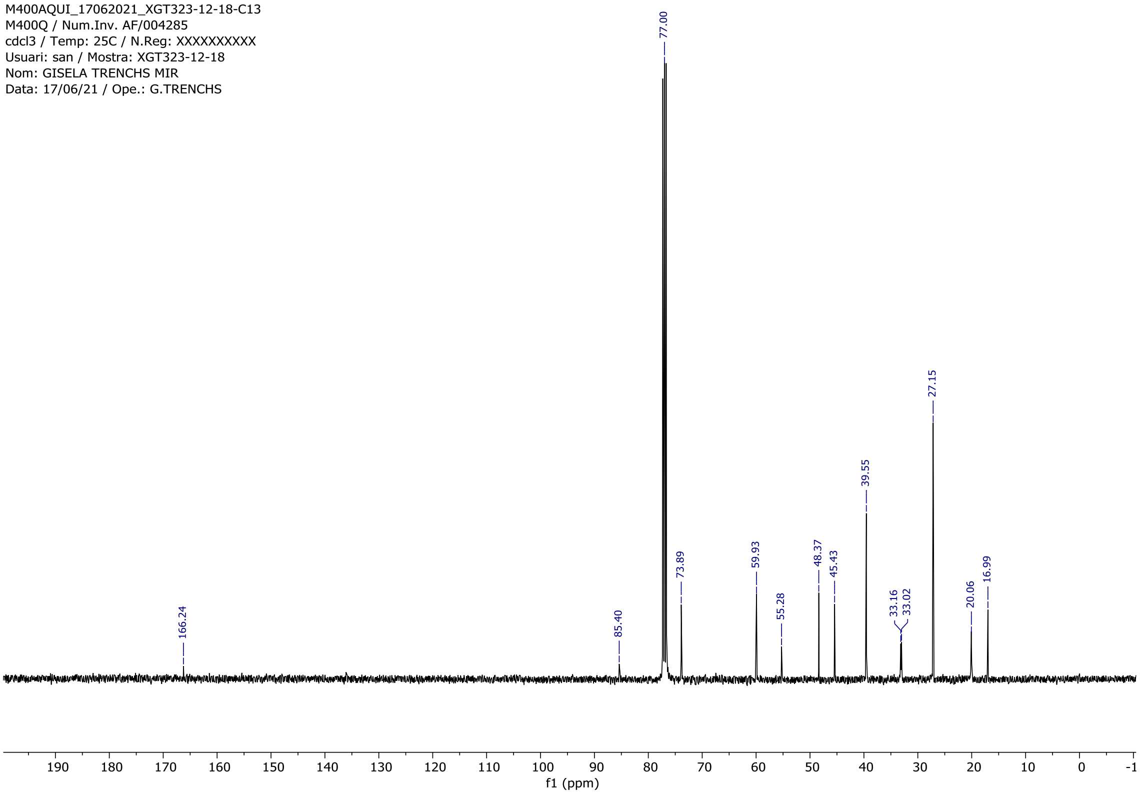

Supplement: Supplementary file 1 [file molecules-29-02035-s001.zip › molecules-2970609-supplementary.pdf]
